# Induction of New Aromatic Polyketides from the Marine Actinobacterium *Streptomyces griseorubiginosus* through an OSMAC Approach

**DOI:** 10.3390/md21100526

**Published:** 2023-10-03

**Authors:** Víctor Rodríguez Martín-Aragón, Francisco Romero Millán, Cristina Cuadrado, Antonio Hernández Daranas, Antonio Fernández Medarde, José M. Sánchez López

**Affiliations:** 1 Biomar Microbial Technologies, Parque Tecnológico de León, Parcela M-10.4, Armunia, 24009 León, Spain; v.rodriguez@biomarmt.com (V.R.M.-A.); f.romero@biomarmt.com (F.R.M.); a.fernandez@biomarmt.com (A.F.M.); 2 Instituto de Productos Naturales y Agrobiología, Consejo Superior de Investigaciones Científicas (IPNA-CSIC), 38206 La Laguna, Tenerife, Spain; ccg00051@red.ujaen.es

**Keywords:** aromatic polyketides, *Streptomyces griseorubiginosus*, OSMAC, quantum mechanical calculations

## Abstract

Using the OSMAC (One Strain Many Compounds) approach, the actinobacterium *Streptomyces griseorubiginosus*, derived from an unidentified cnidarian collected from a reef near Pointe de Bellevue in Réunion Island (France), was subjected to cultivation under diverse conditions. This endeavour yielded the isolation of a repertoire of 23 secondary metabolites (**1**–**23**), wherein five compounds were unprecedented as natural products (**19**–**23**). Specifically, compounds **19** and **20** showcased novel anthrone backbones, while compound **23** displayed a distinctive tetralone structure. Additionally, compounds **21** and **22** presented an unusual naphtho [2,3-*c*]furan-4(9*H*)-one chromophore. Interestingly, the detection of all these novel compounds (**19**–**23**) was exclusively achieved when the bacterium was cultured in FA-1 liquid medium supplemented with the epigenetic modifier γ-butyrolactone. The elucidation of the structural features of the newfound compounds was accomplished through a combination of HRESIMS, 1D and 2D NMR spectroscopy, as well as QM-NMR (Quantum Mechanical—Nuclear Magnetic Resonance) methods and by comparison with existing literature. Moreover, the determination of the relative configuration of compound **23** was facilitated by employing the mix-*J*-DP4 computational approach.

## 1. Introduction

Natural products of microbial origin, especially those from actinobacteria, have been proven to be successful drug leads [1,2,3]. However, standard culture conditions often fail to reveal their full biosynthetic potential and leads to re-isolation of already known metabolites. Strategies to unlock silent biosynthetic gene clusters that are not expressed using conventional fermentation methods include the so-called OSMAC (One Strain Many Compounds) approach [4,5,6,7,8,9]. The OSMAC approach has been shown as a simple and powerful tool to promote the production of different secondary metabolites, potentially bioactive, by altering simple culture parameters, such as variations in medium composition or the addition of epigenetic modifiers (chemical elicitors). The fungus *Sphaeropsidales* sp. was known to produce cladospirone bisepoxide, a spirobisnaphthalene compound. However, Bode et al. [10], reported that culturing the same strain in altered cultivation conditions, different media, and cultivation vessels led to the isolation of fourteen additional compounds, including eight new cladospirones B–I. Notably, the highest number of metabolites was produced when this strain was subjected to solid-phase cultivation in P-flasks with wet oat grains as a single substrate.

As part of our ongoing research on marine microorganisms, we investigated the actinobacterium *Streptomyces griseorubiginosus*, derived from an unidentified cnidarian collected at a reef close to Pointe de Bellevue in Réunion Island (France), following an OSMAC approach. *Streptomyces griseorubiginosus* has been previously reported to produce several antibiotic secondary metabolites, such as the angucyclinone-type rubiginones A1, B1, C1, and C2 [11]; the 15-membered cyclic peptides biphenomycins A-C [12]; the glycosilated anthracyclines cinerubins A and B [13]; and the alkaloid reductiomycin [14].

In this study, the most noteworthy effects with regard to an alteration of the bacterial metabolite pattern were detected following the addition of γ-butyrolactone to the FA-1 liquid medium, compared to the standard culture lacking this chemical elicitor. The investigation of the bacterial extract obtained from fermentation of *Streptomyces griseorubiginosus* under these conditions led to the isolation of five new aromatic polyketides (**19**–**23**), in addition to several known compounds, all of which were not detected when the fungus was grown without this activator. Herein, we report the isolation and the structure elucidation of the new metabolites discovered.

## 2. Results and Discussion

### 2.1. Isolation of Secondary Metabolites from S. griseorubiginosus through OSMAC

**Figure 1:**
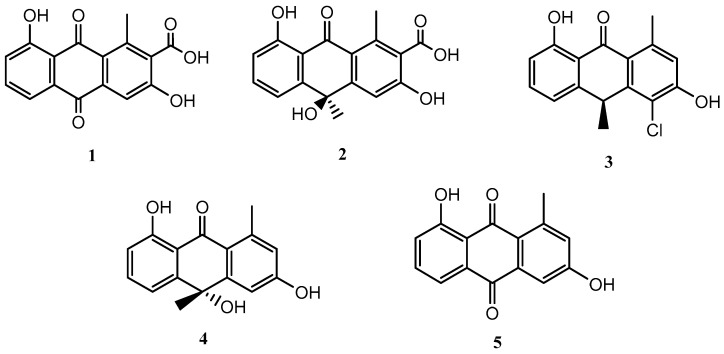
Secondary metabolites isolated from *Streptomyces griseorubiginosus* (FA-1 medium culture).

The fermentation of the microorganism *Streptomyces griseorubiginosus* was carried out under standard conditions (FA-1 medium), and five natural products (**1**–**5**) were isolated and identified; all of them were anthraquinone and anthrone derivatives (Figure 1).

Compounds **1**–**5** were identified as DMAC (**1**) [15,16], (–)-oryzanthrone A (**2**), (+)-chlororyzanthrone B (**3**), (+)-oryzanthrone B (**4**) [17], and aloesaponarin II (**5**) [18], by comparing HRESIMS, 1D and 2D NMR spectra, and their optical rotations with those described in the literature.

*Streptomyces griseorubiginosus* was then cultured on SAF medium, yielding five further secondary metabolites (**6**–**10**), which were not detected from the standard condition culture.

**Figure 2:**
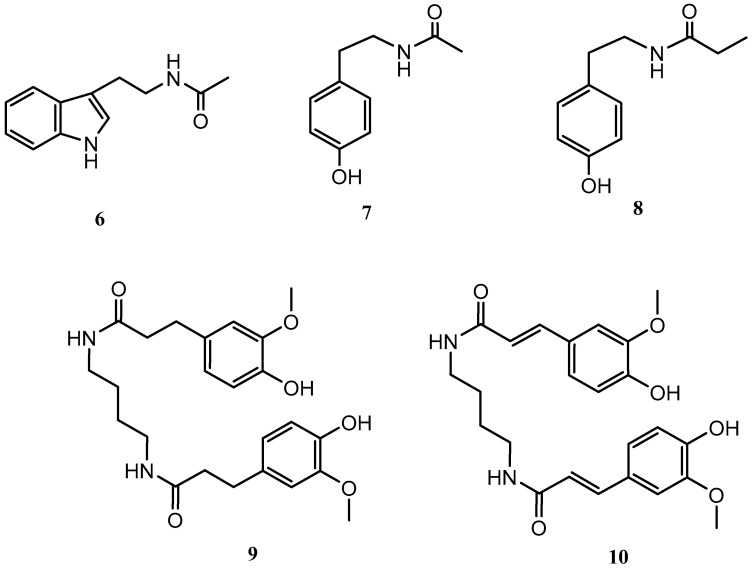
Secondary metabolites isolated from *Streptomyces griseorubiginosus* (SAF medium culture).

Compounds **6**–**10** were identified as *N*-acetyltryptamine (**6**), *N*-acetyltyramine (**7**), *N*-(4-hydroxyphenethyl)propionamide (**8**) [19], JBIR-94 (**9**) [20] and terrestribisamide (**10**) [21] by comparison with the data described in the literature (Figure 2).

**Figure 3:**
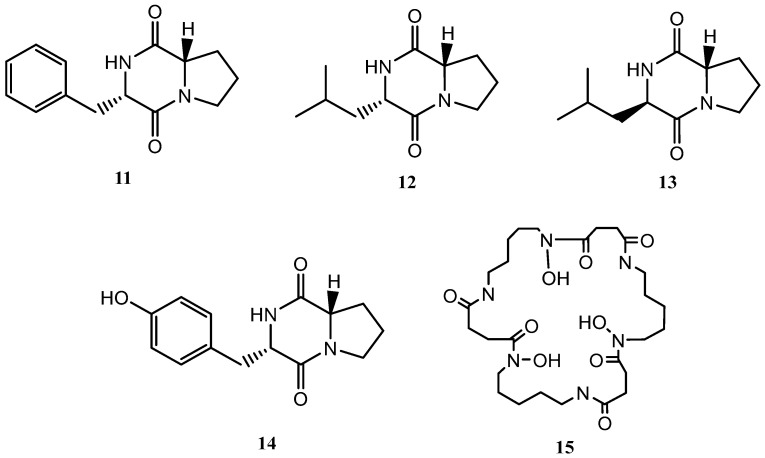
Secondary metabolites isolated from *Streptomyces griseorubiginosus* (SymPV-21 solid medium culture).

Following the OSMAC approach, the microorganism was fermented in a solid medium (SymPV-21). An analysis of the bacterial culture led to the isolation of the known compounds cyclo(L-prolyl-L-phenylalanine) (**11**) [22], cyclo(L-leucyl-L-prolyl) (**12**) [23], cyclo(D-leucyl-L-prolyl) (**13**) [24], maculosin (**14**) [24], and nocardamine (**15**) [25], which were not produced under the previous culture conditions (Figure 3).

The addition of epigenetic modifiers to the culture medium was also studied. These molecules can trigger a new metabolic profile, producing new metabolites or increasing their yield.

**Figure 4:**
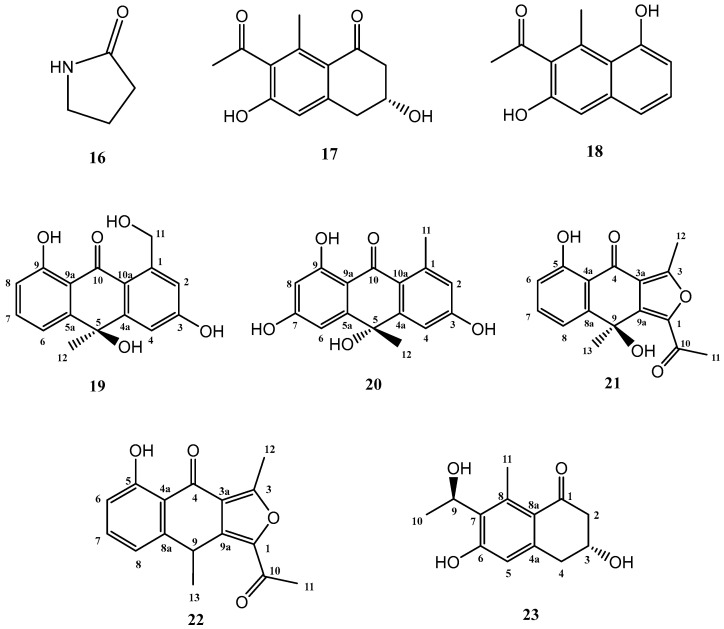
Secondary metabolites isolated from *Streptomyces griseorubiginosus* (FA-1 medium culture + γ-butyrolactone).

The addition of γ-butyrolactone to the standard culture medium (FA-1), led to the isolation of three known compounds (**16**–**18**) and five further secondary metabolites, shown in Figure 4, not previously described in the literature (**19**–**23**).

Known compounds **16**–**18** were identified as γ-butyrolactam (**16**) [26], GTRI-02 (**17**) [27,28], and 6-dehydroxy-GTRI-02 (**18**) [28] after extensive spectroscopic analysis and comparison with data reported in the literature.

### 2.2. Structure Elucidation of Novel Compounds

**Figure 5:**
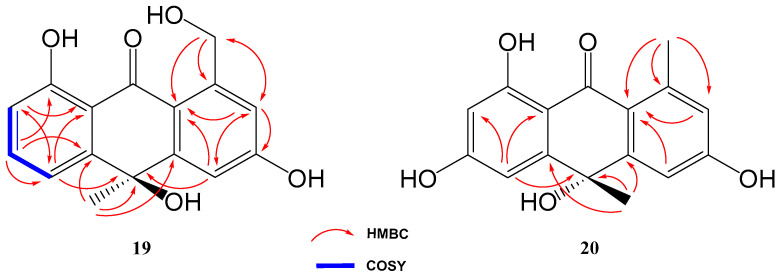
Key COSY and HMBC correlations of compounds **19** and **20**.

Compound **19** was isolated and purified to obtain a yellowish amorphous solid. Analysis by HRESIMS and NMR data determined its molecular formula to be C_16_H_14_O_5_ (ten degrees of unsaturation). 1D NMR spectra were very similar to those of previously isolated anthrone-derived molecules. In comparison with (+)-Oryzanthrone B (**4**), the presence of an oxygenated methylene (δ_H_ 4.66, 4.92, δ_C_ 65.9) was observed instead of the methyl (δ_H_ 2.68, δ_C_ 24.3) attached to the aromatic ring. The gHMBC correlations observed from H_2_-11 to C-1, C-2 and C-10a confirmed the replacement of the methyl group by the oxygenated methylene (Figure 5). Further 2D NMR analysis confirmed that the rest of the molecule was identical to **4**, determining the structure of **19** as shown in Figure 4.

Based on the opposite sign of the optical rotation of compound **19** ([α]^20^_D_ = −133.9°) compared to that published for (+)-Oryzanthrone B ([α]^25^_D_ = +10.7°), we tentatively assign an absolute configuration *R* to carbon 5 in this compound.

Compound **20** was a yellowish amorphous solid with the molecular formula C_16_H_14_O_5_ determined by HRESIMS and NMR data (10 degrees of unsaturation). An initial study of the 1D NMR spectra of **20** (Table 1) revealed that this compound was a new anthrone derivative. Comparison of the ^1^H and ^13^C NMR spectra with those of (+)-Oryzanthrone B (**4**) showed the similarity between these two molecules, the only difference being the presence of a hydroxy group attached to C-7. The molecular mass of **20**, 16 amu higher than that of **4**, supported this suggestion. ^1^H-NMR and gCOSY experiments suggested the presence of an anthrone scaffold, with two tetrasubstituted aromatic rings with two protons in meta each (*J*_2–4_ = 2.5 Hz; *J*_6–8_ = 2.4 Hz). These data together with the observed correlations in gHMBC experiments from H-2 and H-4 to C-10a and from H-6 to C-5, C-8 and C-9a confirmed the structure of compound **20** as shown in Figure 4 and Figure 5.

Moreover, compound **20** showed an optical rotation ([α]^20^_D_ = +2.2°) similar in sign and magnitude to that of (+)-Oryzanthrone B ([α]^25^_D_ = +10.7°), in contrast to the negative optical rotation of compound **19** ([α]^20^_D_ = −133.9°), so we tentatively assign absolute configuration *S* to carbon 5 in compound **20**.

The third novel natural product produced by *Streptomyces griseorubiginosus* using γ-butyrolactone as an epigenetic modifier on FA-1 medium was a yellowish amorphous solid (**21**) with the molecular formula C_16_H_14_O_5_, established by HRESIMS and NMR data (10 degrees of unsaturation).

**Figure 6:**
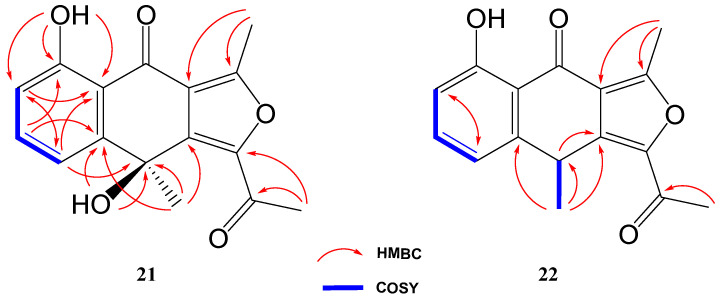
Key COSY and HMBC correlations of compounds **21** and **22**.

The ^1^H NMR spectrum of compound **21** was very similar to that of previously elucidated anthrone-derived molecules; however, more detailed analysis of ^13^C and 2D NMR experiments suggested some differences in the chemical scaffold of **21**. The gHMBC correlations (see Table 2) and chemical shifts of H-6, H-7, H-8, and H_3_-13 confirmed that the naphtho moiety was identical to that of compounds **2**, **4,** and **19**. On the other side, gHMBC correlations from H_3_-12 to C-3 (δ_C_ 161.6) and C-3a (δ_C_ 117.5) and from H_3_-11 to C-1 (δ_C_ 145.6) and C-10 (δ_C_ 190.4), shown in Figure 6, in conjunction with molecular weight information, suggested that compound **21** was 1-acetyl-5,9-dihydroxy-3,9-dimethylnaphtho[2,3-*c*]furan-4(9*H*)-one. The structure of **21** was subsequently confirmed by comparison with the data described in the literature for other polyketides with the naphtho[2,3-*c*]furan-4(9*H*)-one backbone [29,30].

**Figure 7:**
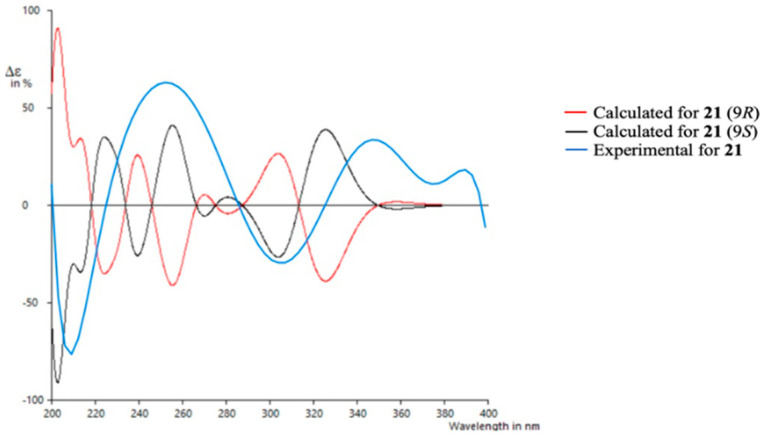
Experimental and calculated ECD spectra for compound **21**.

To determine the absolute configuration of **21**, TDDFT/ECD (time-dependent density functional theory/electronic circular dichroism) calculations were performed at the PCM/B3LYP/6-31G(d) level of theory using structures previously optimized at the same level. It has to be noted that due to the rigidity of the molecule only one suitable structure was found in a molecular mechanics conformational search within a threshold of 21 kJ/mol. As observed in Figure 7, the experimental spectrum showed negative Cotton effects at ~210 nm and ~305 nm and positive Cotton effects at ~250 nm and ~350 nm. The calculated spectrum for the 9*S* enantiomer clearly matched better with the experimental data, allowing us to assign the absolute configuration of **21**. Similar results were obtained using a different level of theory (ωB97XD) (see Supporting Information).

Compound **22** was isolated as a whitish amorphous solid with the molecular formula C_16_H_14_O_4_ determined based on HRESIMS and NMR data (10 degrees of unsaturation). ^1^H NMR spectrum of compound **22** suggested a very similar structure to that of **21**, the only difference being a quadruplet methine group (δ_H_ 4.58, δ_C_ 32.2) coupled to H_3_-13 methyl group (*J* = 7.1 Hz) in **22**, instead of the quaternary carbon (δ_C_ 67.7) at C-9 in **21**. This suggested the loss of the hydroxy group attached to C-9 in **21**, which was also evident from the molecular mass of **22** being 16 amu less than that of **21** (C_16_H_14_O_5_), establishing compound **22** as 1-acetyl-5-hydroxy-3,9-dimethylnaphtho[2,3-*c*]furan-4(9*H*)-one (Figure 4).

**Figure 8:**
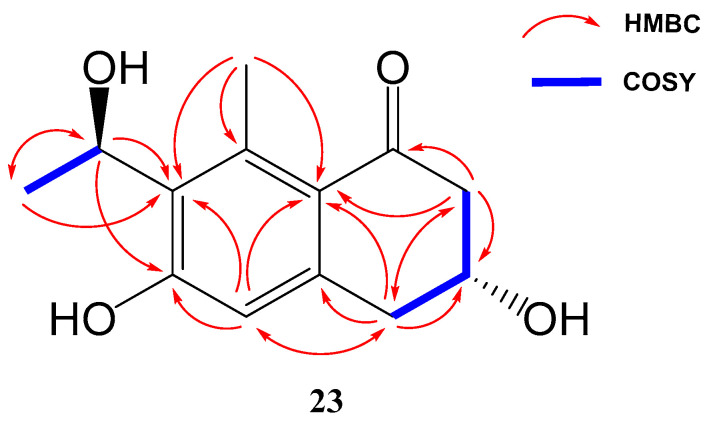
Key COSY and HMBC correlations of compounds **23**.

Compound **23** (yellowish amorphous solid) has the molecular formula C_13_H_16_O_4_ (six degrees of unsaturation) based on HRESIMS and NMR data. NMR data (Table 3) were very similar to those of GTRI-02 (**17**)**.** The presence of a new oxygenated quadruplet methine group (δ_H_ 5.31, δ_C_ 68.0) coupled to H_3_-10 methyl group (*J* = 6.7 Hz) in **23**, instead of the ketone group at C-9 in GTRI-02, suggested the reduction of the ketone to alcohol, which was also evident from the molecular formula of **23** (C_13_H_16_O_4_). Further 2D NMR analysis confirmed the structure of **23** as shown in Figure 4 and Figure 8.

Therefore, two remote chiral centres are present in **23**, making it very difficult to determine their relative configurations. The use of quantum mechanical calculations to unravel the information encoded in NMR chemical shifts, also known as the QM-NMR method, is currently one of the most popular strategies for this purpose. There are several methods for this task; however, those based on Bayes’ theorem, such as the original DP4 probability or the more elaborated DP4+ and *J*-DP4 methods, stand out from the rest [31,32,33,34]. Previous experience with these approaches [35,36,37,38,39,40,41,42] led us to select mix-*J*-DP4 [43] as the most appropriate method in this case, as it yields high confidence results at modest computational costs by incorporating proton–proton NMR coupling constants. Thereby, a conformational search was achieved using the mixed torsional/low-mode protocol in gas phase by means of MMFF force field; later, the conformers were reoptimized with an AMBER and MM3 force field. The energy cut-off of 12 kJ/mol and geometric criteria of MAD 0.5 Å were used to eliminate duplicate structures; after that, NMR calculations were performed at the *J*-DP4 recommended level (B3LYP/6-31G**) and ^3^*J*_HH_ used only the Fermi Contact term contribution. Finally, a comparison of calculated and experimental NMR data was undertaken as described for the *J*-DP4 protocol, obtaining an overall probability of >99% for isomer 3R*,9R*. Moreover, the R^2^s obtained with each force field are: AMBER 0.999 and 0.987, MM3 0.999 and 0.993, MMFF 0.999 and 0.987, for carbon and proton, respectively, and the ^13^C CMAE value for AMBER and MM3 is 1.5 ppm and for MMFF 1.8 ppm. The ^1^H CMAE value obtained for all force fields is 0.1 ppm.

**Figure 9:**
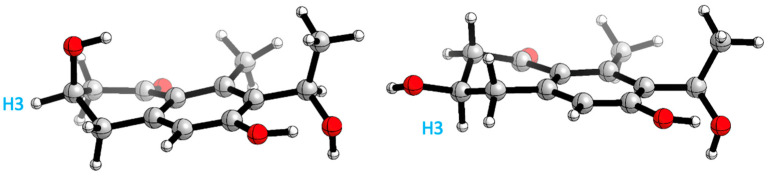
Main conformations H3 axial (**right**) and H3 equatorial (**left**) position.

A conformational search analysis shows that there exist two major conformations (Figure 9): one conformer where H3 is in an axial position with a contribution of 57–65% (depending on the force field used), and the other conformer which represents 35–43% of the total population that has the same proton in an equatorial position. This hints that there exists a conformational equilibrium between both conformations, which agrees with the experimental results obtained in ^3^*J*_H3_ (8.1 and 4.0 Hz).

All isolated compounds were subjected to antibacterial and cytotoxic (against different tumour cell lines) assays, but none of them showed any remarkable bioactivity.

Attempts to obtain more available material of those compounds are currently ongoing, in order to screen them in some relevant crop-protection assays.

In conclusion, the fermentation of *Streptomyces griseorubiginosus* on FA-1 medium afforded five anthraquinone and anthrone-derivative natural products (**1**–**5**). Changing from FA-1 to SAF medium resulted in the production of five further secondary metabolites (**6**–**10**), with structures totally different from those observed under the previous conditions. A switch from liquid media to solid SymPV-21 medium yielded five additional compounds (**11**–**15**), four cyclodipeptides and the hydroxamate siderophore Nocardamine (**15**). Finally, the addition of γ-butyrolactone to FA-1 medium caused the accumulation of eight additional metabolites (**16**–**23**); five of them (**19**–**23**) were novel natural products not previously reported in the literature.

## 3. Materials and Methods

### 3.1. General Experimental Procedures

Optical rotations were measured on a JASCO P-2000 polarimeter using MeOH as solvent. CD spectra were recorded on a JASCO J-810 spectropolarimeter. IR data were recorded using a BRUKER VECTOR 22 spectrophotometer. ^1^H NMR and ^13^C NMR data were obtained on a Varian “Mercury 400” spectrometer at 400 and 100 MHz, respectively. Chemical shifts are reported in ppm relative to solvent (CDCl_3_: δ = 7.26/77.16 ppm; [D_4_] MeOH: δ= 3.31/49.0 ppm). HPLC-MS experiments were carried out with an Agilent UHPLC 1290 Infinity II coupled to an Agilent TOF 6230. HPLC analysis were performed with a Zorbax Eclipse plus C18 column, using a linear gradient from 20% to 100% methanol in 8 min, a post time of 2 min, and a DAD analysis 200–600 nm range signal obtained at 220 nm. Mass spectrometer conditions in ESI-Positive were 100 V Fragmentor, mass range (*m*/*z*) = 100–3200. The analysis were performed with Masshunter suite software (Agilent Technologies) version B.08.00. An Agilent 1200 series liquid chromatograph with a photodiode array and evaporative light-scattering detectors was used for HPLC analysis and recording UV spectra and *t_R_* values, performed with a Zorbax Eclipse XDB-C18 column (4.6 × 50 mm, 1.8 µm) maintained at 20 °C with a mobile phase flow rate of 0.5 mL/min, using the following linear gradient of MeOH/H_2_O (+0.04% TFA): Min 0–15: 15–100% MeOH; min 15–20: 100% MeOH; min 20–30: 100–15% MeOH, post-run 5 min, and a DAD analysis 200–600 nm range signal obtained at 220 nm. Semipreparative HPLC purifications were conducted on an Agilent 1100 series (Kinetex 5 µm EVO C18 10 column, 100 × 21.2 mm).

### 3.2. Bacterial Material

#### 3.2.1. Isolation of the Strain

Strain AA-AR-H-B002 was isolated from an unidentified cnidarian collected at a reef close to Pointe de Bellevue in Réunion Island (France). The strain has been deposited in the Colección Española de Cultivos Tipo, Valencia, Spain, with the accession number CECT 30830.

#### 3.2.2. Identification of the Strain

The strain was identified as *Streptomyces griseorubiginosus* by partial sequencing of its 16S RNA, showing a 99.78% homology in a 913-nucleotide-length sequence. The DNA of the microorganism was obtained using a Qiagen Dneasy kit following the manufacturer’s instructions. The 16S rDNA gene was amplified using the universal primers 27f (AGAGTTTGATCMTGGCTCAG) and 1492r (TACGGYTACCTTGTTACGACTT). The PCR program used was as follows: 94 °C for 1 min, then 30 cycles of 98 °C for 10 s, 50 °C for 2 min, 172 °C for 10 s, and finally one step of 72 °C for 7.5 min. The amplified gen was purified using the Qiagen QiaQuick kit following the manufacturer’s directions. The purified amplicons were sent to an external service for sequencing. All the different steps were monitored using 1.2% agarose electrophoresis in half-strength TB buffer.

### 3.3. Fermentations

All the liquid fermentations were carried out in flasks, incubated at 28 °C and with an agitation at 240 rpm. All the fermentations started from a fresh, confluent culture of the actinomycete on 172 solid medium incubated for one week at 28 °C. The inoculum was developed in two stages. The first stage was carried out in Erlenmeyer flasks of 50 mL capacity and containing 10 mL of medium MIM. These flasks were seeded with 3 plugs taken from the lawn of the confluent solid culture. The flasks were incubated as described above for 48 h. The second stage was carried out in Erlenmeyer flasks of 250 mL capacity filled with 40 mL of MIM medium and seeded with 4 mL of the first stage. The flasks were incubated as described for 48 h. The production phase was performed using Erlenmeyer flasks of 2 L capacity and filled with 250 mL of the appropriate medium. These production flasks were seeded with 12 mL of the second-stage inoculum and incubated as described previously for 96 or 120 h depending on the experiment. The epigenetic factor γ-butyrolactone was added to the corresponding medium (FA-1) at a concentration of 50 µM.

All the solid fermentations were carried out in 2 L capacity Erlenmeyer flasks, containing 250 mL of solid medium SymPv-21. The seeded flasks were incubated at 28 ºC in aerobic conditions for 144 h.

#### Media Compositions

MIM medium per litre: beef extract 3 g, casein peptone 5 g, Hsoy flour 5 g, corn starch 24 g, yeast extract 5 g, glucose 1 g, Na_2_SO_4_ 7.5 g, NaCl 5.34 g, MgCl_2_.6H_2_O 2.4 g, KCl 0.2 g. pH was adjusted to 6.8 and 4 g calcium carbonate was added after adjustment.

FA-1 medium per litre: baker’s yeast 5 g, bacto peptone 1 g, glucidex-12 20 g, soy flour 3 g, glucose 5 g, K_2_HPO_4_ 0.5 g, MnSO_4_.H_2_O 0.0076 g, MgCl_2_.6H_2_O 2.4 g, Na_2_SO_4_ 7.5 g, NaCl 5.34 g, CoCl_2_.6H_2_O 0.0013 g, KCl 0.2 g. pH was adjusted to 7 and 4 g calcium carbonate was added after adjustment.

SAF medium per litre: mannitol 40 g, glucose 10 g, atomized corn steep 12.5 g, pharmamedia 16 g, MgSO_4_.7H_2_O 2 g, Na_2_SO_4_ 5.5 g, NH_4_Cl 2 g, KCl 2 g, NaCl 2.34 g, and MnSO_4_.H_2_O 0.0076 g. pH was adjusted to 6.8 and 10 g calcium carbonate was added after adjustment.

SymPv-21 medium per litre: glycerol 15 g, peptone proteose 25 g, pharmamedia 5 g, sodium gluconate 5 g, K_2_HPO_4_ 2 g, and MgSO_4_.7H_2_O 2 g. pH was adjusted to 7.2.

Finally, 172 medium per litre: glucose 10 g, glucidex-12 20 g, yeast extract 5 g, casein peptone 5 g, and agar 15 g. pH was adjusted to 6.8 and 1 g calcium carbonate was added after adjustment.

### 3.4. Extraction and Isolation

Fermentation broth (8 L) of *Streptomyces griseorubiginosus* under standard conditions (FA-1 liquid medium) was stirred with Amberlite XAD1180N™ resin (10% *v*/*v*) for 1 h. Then, culture broth was filtered through Celite^®^, and the mycelial cake and the resin were extracted with 4 L of a mixture of EtOAc/MeOH (3:1). The resultant suspension was filtered and partitioned between EtOAc and water. The organic layer was taken to dryness, and the crude extract (3.9 g) was fractionated by VFC (vacuum-flash chromatography) on silica gel, eluting with a stepwise gradient of hexane/EtOAc/MeOH to give 12 fractions (1F1–1F12). Fractions 1F11–12 were further fractionated by normal-phase liquid chromatography using a stepwise gradient of CH_2_Cl_2_ and MeOH to yield 12 subfractions (2F1–2F12). Subfractions 2F7–10 and 2F5 were purified by reversed-phase liquid chromatography using a stepwise gradient of MeOH/H_2_O to give **1** (3.7 mg, *t_R_* 14.2 min) and **2** (13.2 mg, *t_R_* 12.3 min), respectively. Compounds **3** (12.5 mg, *t_R_* 16.1 min) and **5** (27.4 mg, *t_R_* 15.8 min) were isolated from fraction 1F5 by normal-phase liquid chromatography with a gradient of hexane/EtOAc followed by a further step of normal-phase liquid chromatography with a gradient of hexane/ether, while compound **4** (74.5 mg, *t_R_* 13.9 min) was obtained from fractions 1F6–7, following a similar procedure, with the difference that the last step of purification was carried out by normal-phase liquid chromatography with a gradient of CH_2_Cl_2_/MeOH.

The same extraction procedure was followed for all liquid medium fermentations. The chromatographic work up of the OSMAC extracts followed the same procedure as described for the standard-condition culture. Silica gel vacuum-flash chromatography using a stepwise gradient of hexane/EtOAc/MeOH was used for separation of the crude extracts obtained from SAF medium (4.55 g) and FA-1 medium with the addition of the chemical elicitor γ-butyrolactone (4.94 g). A total of twelve fractions (SA1F1–SA1F12) were obtained from the SAF culture extract. Fractions SA1F9–10 were further fractionated by normal-phase liquid chromatography using a stepwise gradient of CH_2_Cl_2_/MeOH to yield 10 subfractions (SA2F1–SA2F10). Subfraction SA2F5 was purified by reversed-phase liquid chromatography using a stepwise gradient of MeOH/H_2_O to give **6** (8.4 mg, *t_R_* 9.0 min), while subfractions SA2F7 and SA2F8 were also purified by MeOH/H_2_O reversed-phase liquid chromatography, yielding **7** (15.1 mg, *t_R_* 5.8 min), **8** (5.6 mg, *t_R_* 7.3 min), **9** (4.6 mg, *t_R_* 9.7 min), and **10** (3 mg, *t_R_* 10.9 min). Fraction B1F11, obtained by VFC from FA-1 culture after the addition of γ-butyrolactone extract, was fractionated by normal-phase liquid chromatography using a stepwise gradient of CH_2_Cl_2_/MeOH to yield 12 subfractions (B2F1–B2F12). Compound **16** (13.9 mg, *t_R_* 1.2 min) was isolated from subfractions B2F5–7 after purification by reversed-phase liquid chromatography using a stepwise gradient of MeOH/H_2_O. Fractions B1F9–10 were also fractionated following the same procedure described for compound **16** to give **17** (46.9 mg, *t_R_* 10.9 min), **18** (4.6 mg, *t_R_* 14.2 min), and **23** (3.2 mg, *t_R_* 9.3 min). Compounds **19** (2 mg, *t_R_* 11.3 min), **20** (3 mg, *t_R_* 14.8 min), **21** (3.1 mg, *t_R_* 17.3 min), and **22** (1.1 mg, *t_R_* 12.6 min) were isolated from B1F5 (**21** and **22**), B1F7 (**19**), and B1F8 (**20**), respectively, after purification by normal-phase liquid chromatography using a stepwise gradient of hexane/EtOAc, followed by semi-preparative HPLC purification with a gradient of MeOH/H_2_O as an eluting system.

In the case of the solid-state fermentation (SymPV-21 medium), the contents of the flasks were collected and homogenized with the aid of an electric mixer, followed by extraction with 100% *v*/*v* of a mixture of EtOAc/MeOH (3:1). Twelve fractions were obtained from the SymPV-21 solid culture extract (4.26 g) after VFC (Sy1F1–Sy1F12). Compounds **11** (41.1 mg, *t_R_* 8.6 min), **12** (56 mg, *t_R_* 8.0 min), and **13** (7.8 mg, *t_R_* 7.7 min) were isolated from Sy1F10 after purification by normal-phase liquid chromatography using a stepwise gradient of CH_2_Cl_2_/MeOH, followed by a further step of normal-phase liquid chromatography with a gradient of CH_2_Cl_2_/acetone. Finally, fraction Sy1F11–12 was also fractionated by normal-phase liquid chromatography, using a gradient of CH_2_Cl_2_/MeOH as mobile phase, followed by reversed-phase liquid chromatography using a stepwise gradient of MeOH/H_2_O to afford **14** (27.5 mg, *t_R_* 5.0 min) and **15** (8.4 mg, *t_R_* 9.7 min).

### 3.5. Spectroscopic Data of Compounds **19**–**23**

**Compound 19.** Yellowish amorphous solid; [α]^20^_D_ = −133.9° (c 0.2, MeOH); UV (MeOH) λ_max_ (log *ε*) 210 (2.41), 244 (2.12), 270 (1.95), 320 (2.13), 340 (2.09) nm; IR (ATR) *ν*_max_ 3282 (br), 2977, 2928, 1624, 1594, 1458, 1332, 1269, 1242, 1172 cm^−1^; for ^1^H and ^13^C NMR data, see Table 1; HRESIMS *m*/*z* 287.0907 [M + H]^+^ (calcd for C_16_H_15_O_5_, 287.0919).

**Compound 20.** Yellowish amorphous solid; [α]^20^_D_ = +2.2° (c 0.136, MeOH); UV (MeOH) λ_max_ (log *ε*) 210 (2.16), 242 (1.89), 280 (1.73), 350 (1.98) nm; IR (ATR) *ν*_max_ 3397 (br), 2969, 2926, 1627, 1608, 1577, 1473, 1316, 1270, 1176 cm^−1^; for ^1^H and ^13^C NMR data, see Table 1; HRESIMS *m*/*z* 287.0911 [M + H]^+^ (calcd for C_16_H_15_O_5_, 287.0919).

**Compound 21.** Yellowish amorphous solid; [α]^20^_D_ = +0.35° (c 0.173, MeOH); UV (MeOH) λ_max_ (log *ε*) 210 (2.22), 232 (2.11), 276 (2.22), 308 (1.95), 344 (1.80) nm; IR (ATR) *ν*_max_ 3365 (br), 2918, 2850, 1650, 1635, 1580, 1542, 1456, 1418, 1345, 1248, 1175 cm^−1^; for ^1^H and ^13^C NMR data, see Table 2; HRESIMS *m*/*z* 287.0920 [M + H]^+^ (calcd for C_16_H_15_O_5_, 287.0919).

**Compound 22.** Whitish amorphous solid; UV (MeOH) λ_max_ (log *ε*) 218 (2.50), 234 (2.43), 274 (2.53), 308 (2.21) nm; IR (ATR) *ν*_max_ 3388 (br), 2917, 2849, 1653, 1635, 1588, 1541, 1456, 1354, 1244 cm^−1^; for ^1^H and ^13^C NMR data, see Table 2; HRESIMS *m*/*z* 271.0960 [M + H]^+^ (calcd for C_16_H_15_O_4_, 271.0970).

**Compound 23.** Yellowish amorphous solid; [α]^20^_D_ = −47.5° (c 0.226, MeOH); UV (MeOH) λ_max_ (log *ε*) 216 (2.28), 232 (2.21), 280 (2.20) nm; IR (ATR) *ν*_max_ 3382 (br), 2973, 2926, 1715, 1650, 1572, 1457, 1369, 1305, 1243, 1158, 1059 cm^−1^; for ^1^H and ^13^C NMR data, see Table 3; HRESIMS *m*/*z* 237.1140 [M + H]^+^ (calcd for C_13_H_17_O_4_, 237.1127).

### 3.6. Computational Methods

Calculations were performed following the general workflow described in the mix-*J*-DP4 method [43]. As previously described, all possible isomers were created, and conformational searches achieved using the mixed torsional/low-mode conformational sampling protocol in gas phase with the MMFF force field and reoptimizing the conformers with an AMBER and MM3 force field. NMR calculations were achieved using the conformations under 12 kJ/mol energy cut-off and MAD 0.5 Å. The DFT levels of theory as recommended for *J*-DP4 (B3LYP/6-31G**) in Gaussian 16 [44], were used to calculate ^3^*J*_HH_, employing exclusively the Fermi Contact term contribution and magnetic shielding constants (σ) by means of the gauge, including the atomic orbital method [45,46,47,48]. Unscaled chemical shifts (δ_u_) were calculated using TMS as reference standard according to the following expression: δ_u_ = σ_0_ − σ_x_, where σ_x_ is the Boltzmann-averaged shielding tensor (over all significantly populated conformations) and σ_0_ is the shielding tensor of TMS computed at the same level of theory used to calculate σ_x_. Boltzmann averaging was performed according to the following equation:

$$\sigma^{x}=\frac{\sum_{i}\sigma_{i}^{x}e^{\left(-\frac{E_{i}}{RT}\right)}}{\sum_{i}e^{\left(-\frac{E_{i}}{RT}\right)}}$$

where σ^x^_i_ is the shielding constant for nucleus x in conformer i, R is the molar gas constant (8.3145 J /(K mol)), T is the temperature used for the calculation (298 K), and E_i_ is the relative energy of conformer i (to the lowest energy conformer) obtained from a single-point NMR calculation at the corresponding level of theory. The scaled chemical shifts (δ_s_) were computed as δ_s_ = (δ_u_ − b)/m, where m and b are the slope and intercept, respectively, resulting from a linear regression calculation on a plot of δ_u_ against δ_exp_. All molecular models figures were performed using CYLview20 [49].

## Figures and Tables

**Table 1:** NMR data of compounds **19** and **20.**

	Compound 19 ^a^				Compound 20 ^b^			
C/H	*δ*_H_ (*J* in Hz)	*δ*_C_ *	gCOSY	gHMBC	*δ*_H_ (*J* in Hz)	*δ*_C_	gCOSY	gHMBC
1		147.9				145.6		
2	6.87, s	117.2		C3, 4, C10a, C11	6.62, d (2.5)	120.0	H4	C-10a
3		164.3				**		
4	7.19, s	112.3		C2, C5, C10a	7.21, d (2.5)	111.7	H2	C-10a
4a		156.3				155.8		
5		71.5				71.8		
5a		150.9				153.9		
6	7.25, d (8.2)	116.6	H7, H8	C5, C8, C9a	6.77, d (2.4)	105.7	H8	C-5, C-8, C-9a
7	7.38, t (8.2)	**	H6, H8	C5a, C6, C9		**		
8	6.75, d (8.2)	116.8	H6, H7	C6, C9a	6.16, d (2.4)	102.6	H6	
9		162.8				**		
9a		115.5				109.4		
10		**				189.3		
10a		119.8				120.6		
11	4.66, d (14.3)	65.9		C1, C2, C10a	2.70, s	24.6		C-1, C2, C10a
	4.92, d (14.3)							
12	1.47, s	39.5		C4a, C5, C5a	1.52, s	39.4		C-4a, C5, C5a

^a^ Measured in CDCl_3_ + MeOH-*d*_4_; ^b^ Measured in MeOH-*d*_4_; * Chemical shifts obtained from gHSQC and gHMBC 2D NMR spectra; ** Signal not observed.

**Table 2:** NMR data of compounds **21** and **22** in CDCl_3_.

	Compound 21				Compound 22			
C/H	*δ*_H_ (*J* in Hz)	*δ*_C_	gCOSY	gHMBC	*δ*_H_ (*J* in Hz)	*δ*_C_ **	gCOSY	gHMBC
1		145.6				***		
3		161.6				160.1		
3a		117.5 *				117.7		
4		186.2				***		
4a		114.4				***		
5		163.0				***		
6	6.95, dd (8.0, 1.0)	116.9	H7, H8	C-4a, C8a	6.87, d (8.0)	115.8	H7	C-8
7	7.57, t (8.0)	137.3	H6, H8	C-5, C8a	7.47, t (8.0)	***	H6, H8	
8	7.42, dd (8.0, 1.0)	117.5 *	H6, H7	C-4a, C6, C9	6.91, d (8.0)	115.7	H7	C-6
8a		148.9				148.1		
9		67.7			4.58, q (7.1)	32.2	H13	C-9a
9a		141.4				136.9		
10		190.4				187.9		
11	2.62, s	26.6		C-1, C10	2.54, s	26.7		C-10
12	2.82, s	14.7		C-3, C3a	2.82, s	***		C-3, C3a
13	1.69, s	35.4		C-8a, C9, C9a	1.53, d (7.1)	26.1	H9	C-8a, C9, C9a
5-OH	12.68, s			C-4a, C5, C6	12.82, s			
9-OH	6.05, s			C-8a, C9				

* Overlapped signals. ** Chemical shifts obtained from gHMBC correlations. *** Signal not observed.

**Table 3:** NMR data of compound **23** in CDCl_3_ + MeOH-*d*_4_.

C/H	*δ*_H_ (*J* in Hz)	*δ*_C_	gCOSY	gHMBC
1		198.7		
2	2.53, dd (16.4, 8.1) 2.81, dd (16.4, 8.1)	49.4	H3	C-1, C3, C4, C8a
3	4.21, tt (8.1, 4.0)	65.8	H2, H4	
4	2.83, dd (16.4, 4.0) 3.03, dd (16.4, 4.0)	39.4	H3	C-2, C3, C4a, C5, C8a
4a		143.3		
5	6.51, s	115.5		C-4, C6, C7, C8a
6		160.6		
7		127.5		
8		139.2		
8a		124.3		
9	5.31, q (6.7)	68.0	H10	C-6, C7, C10
10	1.42, d (6.7)	22.0	H9	C-7, C9
11	2.42, s	16.4		C-7, C8, C8a

## Data Availability

The data used to prepare this manuscript are contained within the article and its corresponding Supplementary Material.

## Supplementary Materials

The following supporting information can be downloaded at: https://www.mdpi.com/article/10.3390/md21100526/s1. Figures S1–S35: NMR spectra of known compounds **1**–**18**. Figures S36–S72: NMR, HRESIMS, UV and IR spectra of novel compounds **19**–**23**. Figures S73–S75: Correlation of compounds **23**; Figures S76 and S77: ECD spectra for compound **21**, Tables S1–S12: Computational section.
