# Supplementary material for: Induction of New Aromatic Polyketides from the Marine Actinobacterium Streptomyces griseorubiginosus through an OSMAC Approach
Source: Mar Drugs. 2023 Oct 3;21(10):526. doi: 10.3390/md21100526 (PMC10608293; doi:10.3390/md21100526)
Supplement: Supplementary file 1 [file marinedrugs-21-00526-s001.zip › marinedrugs-2554647-supplementary.pdf]

Supporting Information

Induction of new aromatic polyketides from the marine actinobacterium *Streptomyces griseorubiginosus* through an OSMAC approach.

Víctor Rodríguez Martín-Aragón [1], Francisco Romero Millán [1], Cristina Cuadrado [2], Antonio Hernández Daranas [2], Antonio Fernández Medarde [1] and José M. Sánchez López [1]**

[1] Biomar Microbial Technologies, Parque Tecnológico de León, Parcela M-10.4, 24009 Armunia, León. Spain

[2] Instituto de Productos Naturales y Agrobiología, Consejo Superior de Investigaciones Científicas (IPNA-CSIC), La Laguna, 38206 Tenerife, Spain

* Corresponding authors:

adaranas@ipna.csic.es (Antonio Hernández Daranas)

jm.sanchez@biomarmt.com (José M. Sánchez López)

Table of contents

1. NMR spectra of known compounds 1-18 4

Figure S1: ^{1}H NMR (400 MHz, DMSO-d_6) spectrum of compound **1** 4
Figure S2: ^{1}H NMR (400 MHz, DMSO-d_6) spectrum of compound **2** 4
Figure S3: ^{13}C NMR (100 MHz, DMSO-d_6) spectrum of compound **2** 5
Figure S4: ^{1}H NMR (400 MHz, $CDCl_3$) spectrum of compound **3** 5
Figure S5: ^{13}C NMR (100 MHz, $CDCl_3$) spectrum of compound **3** 6
Figure S6: ^{1}H NMR (400 MHz, $CDCl_3$ + MeOH-d_4) spectrum of compound **4** 6
Figure S7: ^{13}C NMR (100 MHz, $CDCl_3$ + MeOH-d_4) spectrum of compound **4** 7
Figure S8: ^{1}H NMR (400 MHz, DMSO-d_6) spectrum of compound **5** 8
Figure S9: ^{13}C NMR (100 MHz, DMSO-d_6) spectrum of compound **5** 8
Figure S10: ^{1}H NMR (400 MHz, $CDCl_3$ + MeOH-d_4) spectrum of compound **6** 9
Figure S11: ^{13}C NMR (100 MHz, $CDCl_3$ + MeOH-d_4) spectrum of compound **6** 9
Figure S12: ^{1}H NMR (400 MHz, MeOH-d_4) spectrum of compound **7** 10
Figure S13: ^{13}C NMR (100 MHz, MeOH-d_4) spectrum of compound **7** 10
Figure S14: ^{1}H NMR (400 MHz, MeOH-d_4) spectrum of compound **8** 11
Figure S15: ^{13}C NMR (100 MHz, MeOH-d_4) spectrum of compound **8** 11
Figure S16: ^{1}H NMR (400 MHz, $CDCl_3$ + MeOH-d_4) spectrum of compound **9** 12
Figure S17: ^{13}C NMR (100 MHz, $CDCl_3$ + MeOH-d_4) spectrum of compound **9** 12
Figure S18: ^{1}H NMR (400 MHz, $CDCl_3$ + MeOH-d_4) spectrum of compound **10** 13
Figure S19: ^{13}C NMR (100 MHz, $CDCl_3$ + MeOH-d_4) spectrum of compound **10** 13
Figure S20: ^{1}H NMR (400 MHz, $CDCl_3$) spectrum of compound **11** 14
Figure S21: ^{13}C NMR (100 MHz, $CDCl_3$) spectrum of compound **11** 14
Figure S22: ^{1}H NMR (400 MHz, $CDCl_3$) spectrum of compound **12** 15
Figure S23: ^{13}C NMR (100 MHz, $CDCl_3$) spectrum of compound **12** 15
Figure S24: ^{1}H NMR (400 MHz, $CDCl_3$) spectrum of compound **13** 16
Figure S25: ^{13}C NMR (100 MHz, $CDCl_3$) spectrum of compound **13** 16
Figure S26: ^{1}H NMR (400 MHz, MeOH-d_4) spectrum of compound **14** 17
Figure S27: ^{13}C NMR (100 MHz, MeOH-d_4) spectrum of compound **14** 17
Figure S28: ^{1}H NMR (400 MHz, MeOH-d_4) spectrum of compound **15** 18
Figure S29: ^{13}C NMR (100 MHz, MeOH-d_4) spectrum of compound **15** 18
Figure S30: ^{1}H NMR (400 MHz, MeOH-d_4) spectrum of compound **16** 19
Figure S31: ^{13}C NMR (100 MHz, MeOH-d_4) spectrum of compound **16** 19
Figure S32: ^{1}H NMR (400 MHz, MeOH-d_4) spectrum of compound **17** 20
Figure S33: ^{13}C NMR (100 MHz, MeOH-d_4) spectrum of compound **17** 20
Figure S34: ^{1}H NMR (400 MHz, $CDCl_3$) spectrum of compound **18** 21
Figure S35: ^{13}C NMR (100 MHz, $CDCl_3$) spectrum of compound **18** 21

2. Experimental spectra of novel compounds 19-23 22

Figure S36: HRESIMS spectrum of compound **19** 22
Figure S37: UV absorption spectrum of compound **19** 23
Figure S38: IR spectrum of compound **19** 23
Figure S39: ^{1}H NMR (400 MHz, $CDCl_3$ + MeOH-d_4) spectrum of compound **19** 23
Figure S40: ^{1}H-^{1}H COSY (400 MHz, $CDCl_3$ + MeOH-d_4) spectrum of compound **19** 24
Figure S41: gHSQC (400 MHz and 100MHz, $CDCl_3$ + MeOH-d_4) spectrum of compound **19** 25
Figure S42: gHMBC (400 MHz and 100MHz, $CDCl_3$ + MeOH-d_4) spectrum of compound **19** 25
Figure S43: HRESIMS spectrum of compound **20** 26
Figure S44: UV absorption spectrum of compound **20** 26
Figure S45: IR spectrum of compound **20** 26
Figure S46: ^{1}H NMR (400 MHz, MeOH-d_4) spectrum of compound **20** 27
Figure S47: ^{13}C NMR (100 MHz, MeOH-d_4) spectrum of compound **20** 27
Figure S48: ^{1}H-^{1}H COSY (400 MHz, MeOH-d_4) spectrum of compound **20** 28
Figure S49a: gHSQC (400 MHz and 100MHz, MeOH-d_4) spectrum of compound **20** 28
Figure S49b: gHSQC (400 MHz and 100MHz, MeOH-d_4) spectrum of compound **20** 29
Figure S50a: gHMBC (400 MHz and 100MHz, MeOH-d_4) spectrum of compound **20** 29
Figure S50b: gHMBC (400 MHz and 100MHz, MeOH-d_4) spectrum of compound **20** 30

Figure S51: HRESIMS spectrum of compound **21** 31
Figure S52: UV absorption spectrum of compound **21** 31
Figure S53: IR spectrum of compound **21** 31
Figure S54: ^{1}H NMR (400 MHz, $CDCl_3$) spectrum of compound **21** 32
Figure S55: ^{13}C NMR (100 MHz, $CDCl_3$) spectrum of compound **21** 32
Figure S56: ^{1}H-^{1}H COSY (400 MHz, $CDCl_3$) spectrum of compound **21** 33
Figure S57a: gHSQC (400 MHz and 100MHz, $CDCl_3$) spectrum of compound **21** 33
Figure S57b: gHSQC (400 MHz and 100MHz, $CDCl_3$) spectrum of compound **21** 34
Figure S58a: gHMBC (400 MHz and 100MHz, $CDCl_3$) spectrum of compound **21** 34
Figure S58b: gHMBC (400 MHz and 100MHz, $CDCl_3$) spectrum of compound **21** 35
Figure S58c: gHMBC (400 MHz and 100MHz, $CDCl_3$) spectrum of compound **21** 35
Figure S59: HRESIMS spectrum of compound **22** 36
Figure S60: UV absorption spectrum of compound **22** 36
Figure S61: IR spectrum of compound **22** 36
Figure S62: ^{1}H NMR (400 MHz, $CDCl_3$) spectrum of compound **22** 37
Figure S63: ^{1}H-^{1}H COSY (400 MHz, $CDCl_3$) spectrum of compound **22** 37
Figure S64a: gHMBC (400 MHz and 100MHz, $CDCl_3$) spectrum of compound **22** 37
Figure S64b: gHMBC (400 MHz and 100MHz, $CDCl_3$) spectrum of compound **22** 38
Figure S65: HRESIMS spectrum of compound **23** 39
Figure S66: UV absorption spectrum of compound **23** 39
Figure S67: IR spectrum of compound **23** 39
Figure S68: ^{1}H NMR (400 MHz, $CDCl_3$ + MeOH-d_4) spectrum of compound **23** 40
Figure S69: ^{13}C NMR (100 MHz, $CDCl_3$ + MeOH-d_4) spectrum of compound **23** 40
Figure S70: ^{1}H-^{1}H COSY (400 MHz, $CDCl_3$ + MeOH-d_4) spectrum of compound **23** 41
Figure S71: gHSQC (400 MHz and 100MHz, $CDCl_3$ + MeOH-d_4) spectrum of compound **23** 41
Figure S72: gHMBC (400 MHz and 100MHz, $CDCl_3$ + MeOH-d_4) spectrum of compound **23** 42

3. Computational section* *43

3.1. Computational details 43

3.2. Isomer studied for compound 23. 44

3.3. Experimental chemical shifts and isotropic magnetic shielding constants of studied isomers. 44

3.4. Mix-*J*-DP4 results 47

3.5. CMAE for ^{1}H and ^{13}C for the isomers of compounds 23 48

3.6. Correlation plots of compound 23. 50

3.7. SCF energies of compound 23. 52

3.8. Cartesian coordinates of conformers for compound 23. 54

4. Electronic Circular Dichroism* *59

4.1. Computational details 59

1. NMR spectra of known compounds 1-18

Figure S1: ^{1}H NMR (400 MHz, DMSO-d_6) spectrum of compound **1**

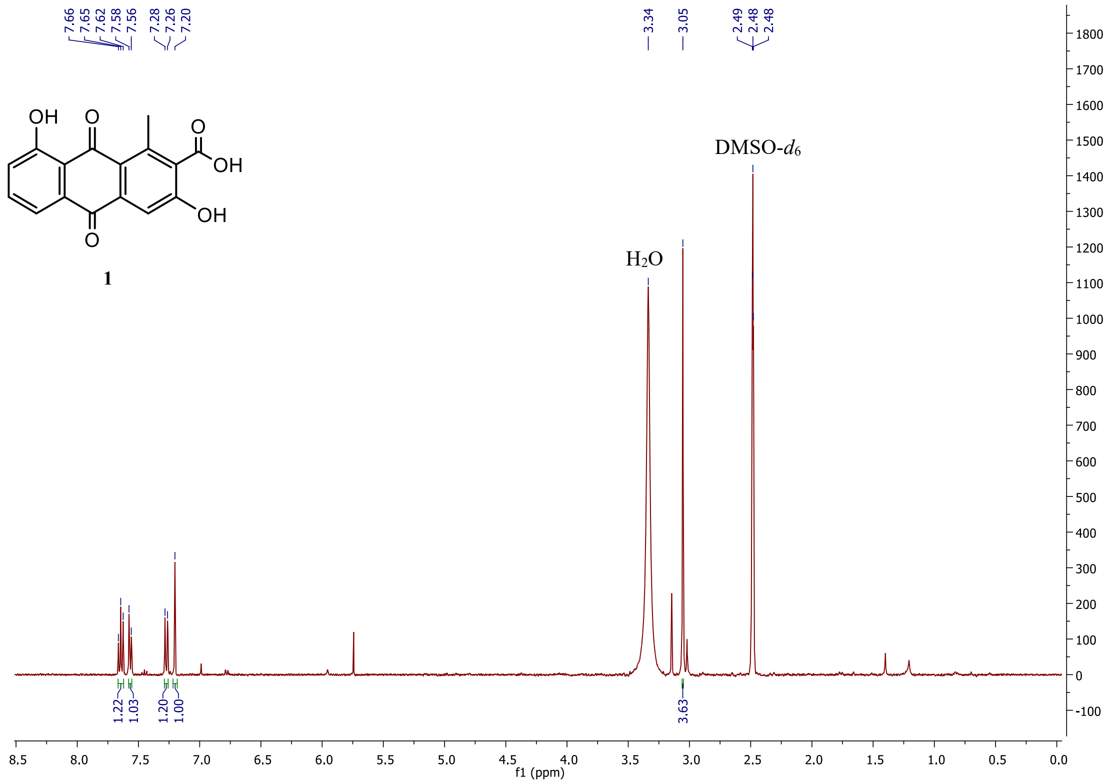

Figure S2: ^{1}H NMR (400 MHz, DMSO-d_6) spectrum of compound **2**

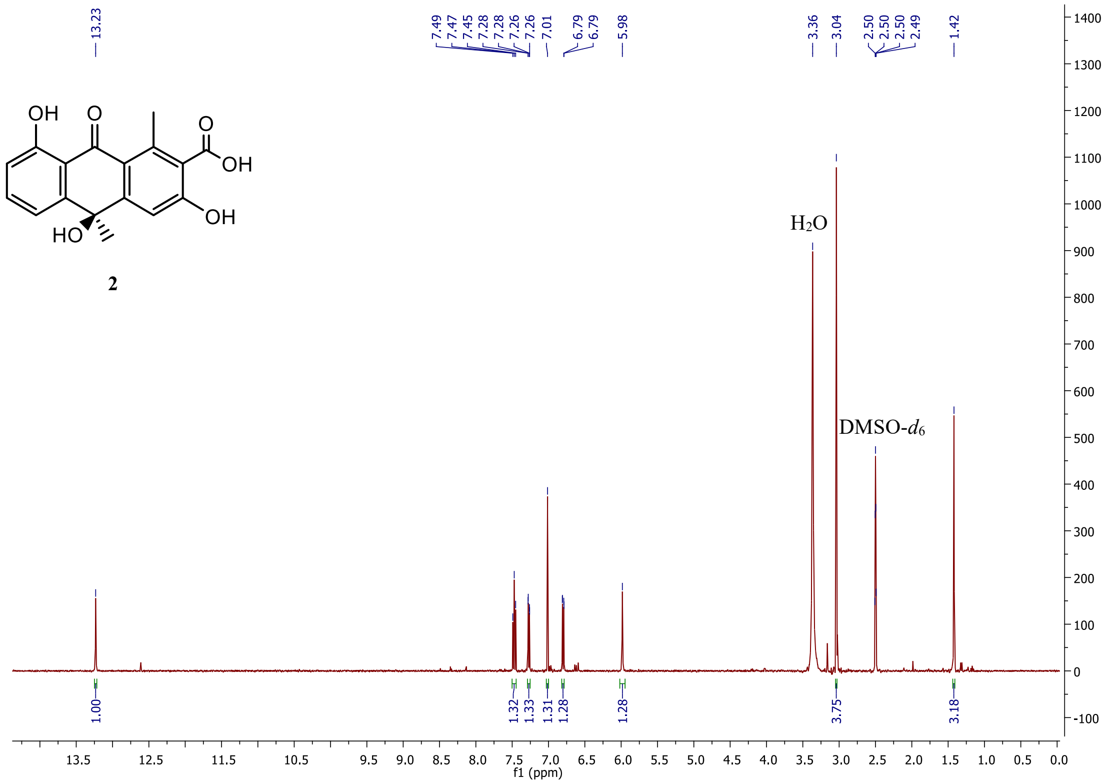

Figure S3: ^{13}C NMR (100 MHz, DMSO-d_6) spectrum of compound **2**

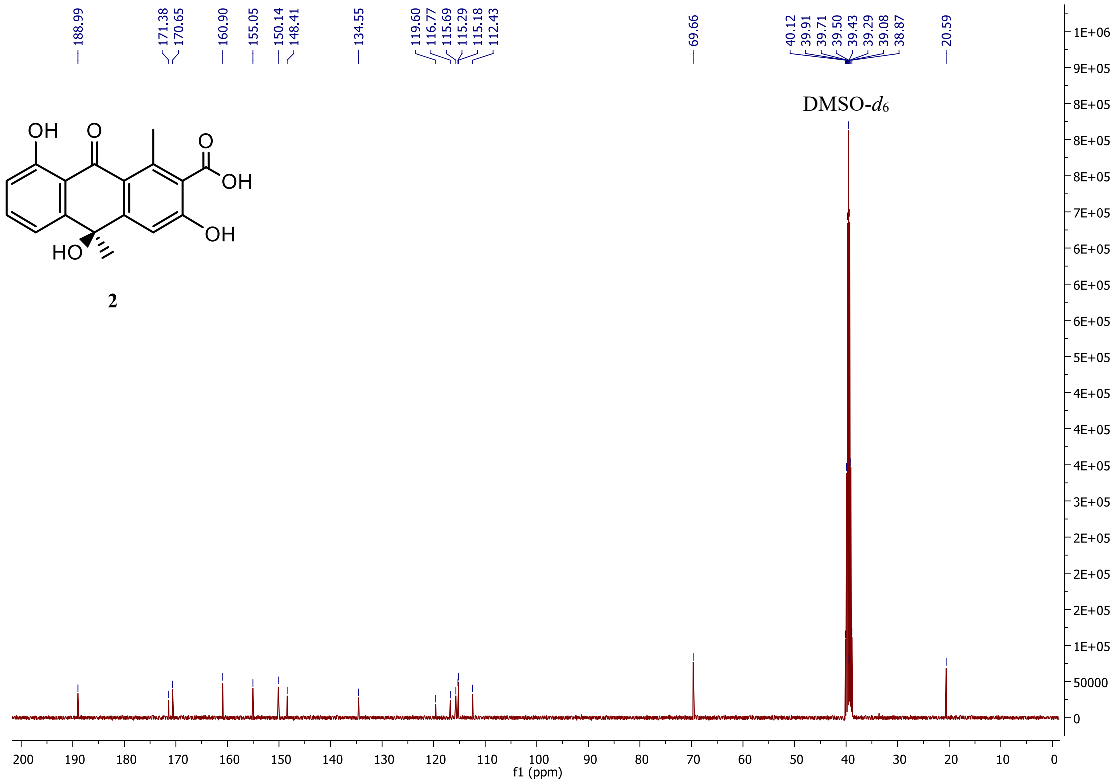

Figure S4: ^1H NMR (400 MHz, $CDCl_3$) spectrum of compound **3**

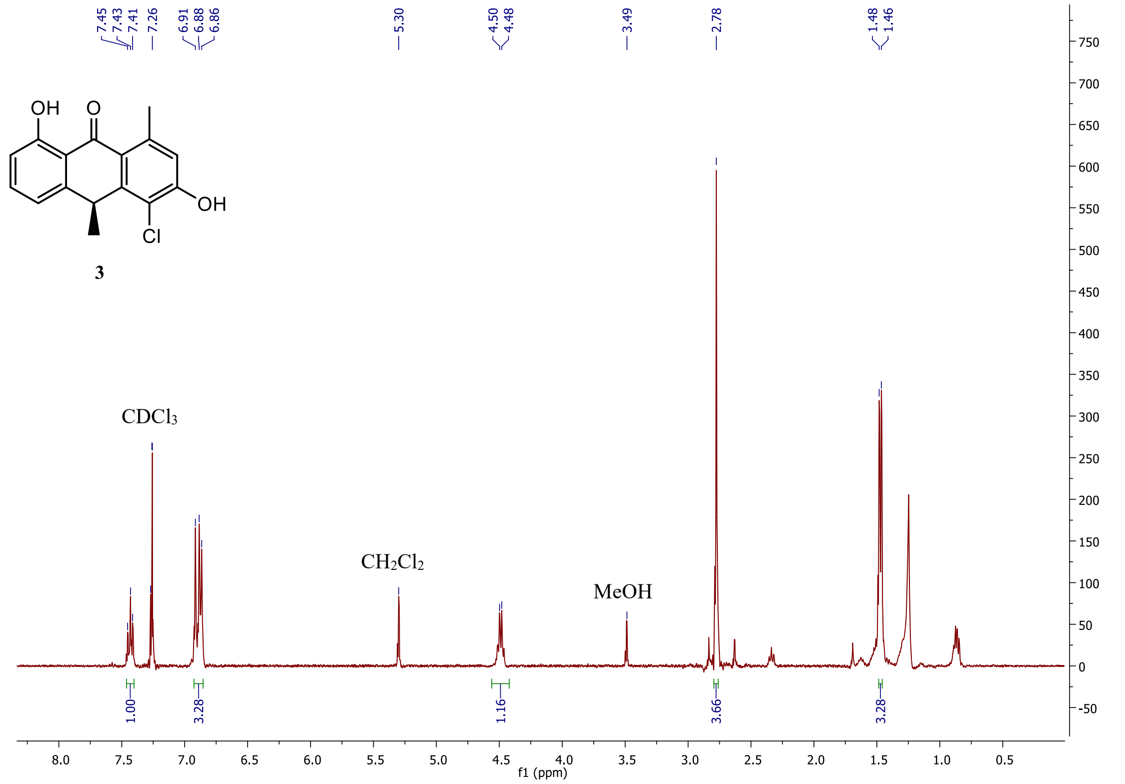

Figure S5: ^{13}C NMR (100 MHz, $CDCl_3$) spectrum of compound **3**

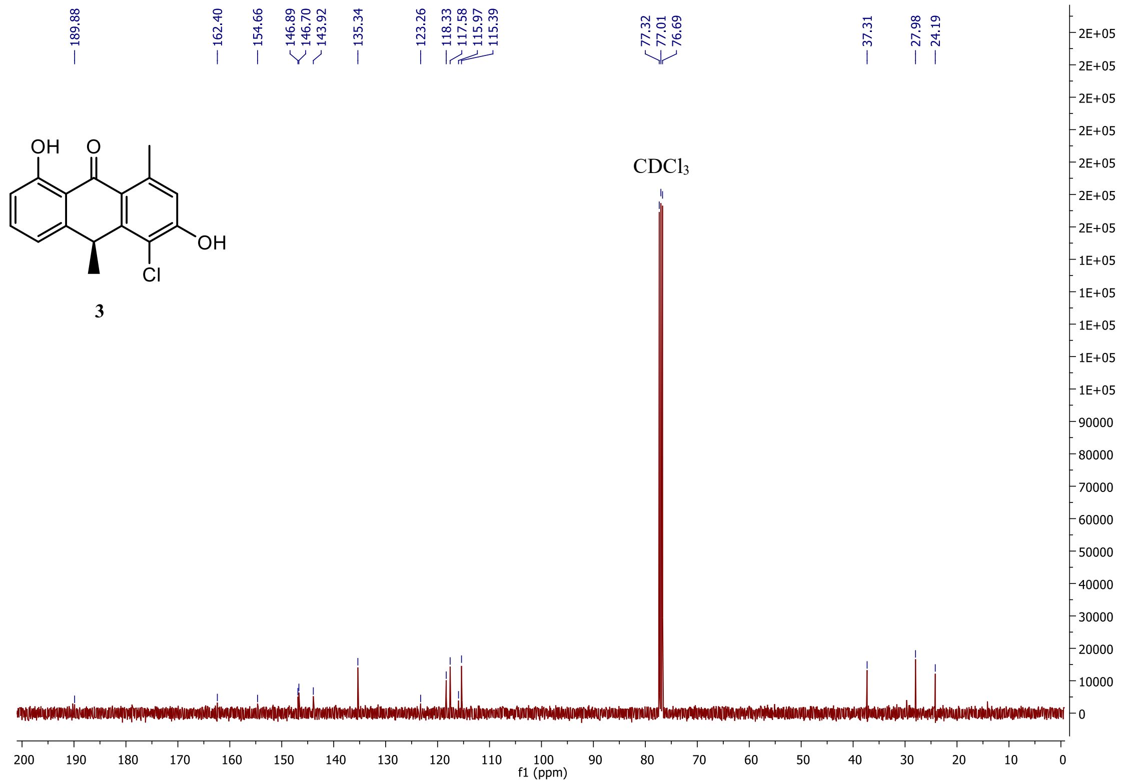

Figure S6: ^{1}H NMR (400 MHz, $CDCl_3$ + MeOH-d_4) spectrum of compound **4**

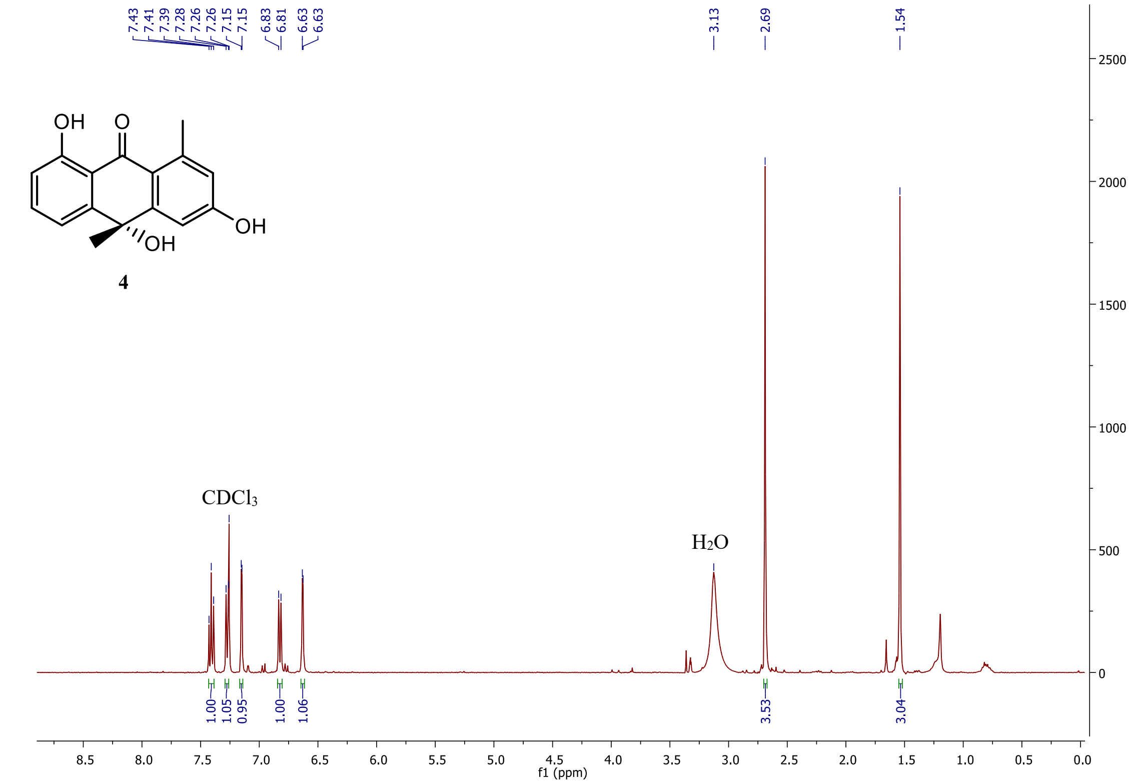

Figure S7: ^{13}C NMR (100 MHz, $CDCl_3$ + MeOH-d_4) spectrum of compound **4**

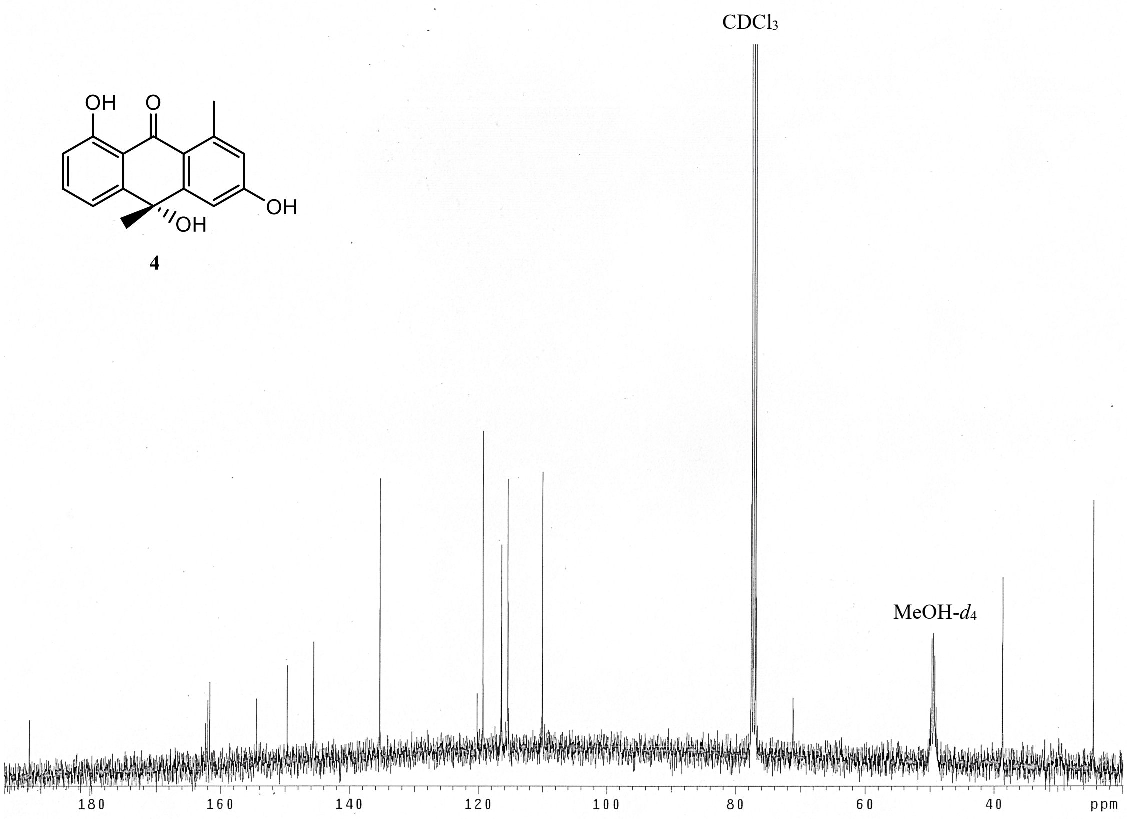

Figure S8: ^{1}H NMR (400 MHz, DMSO-d_6) spectrum of compound **5**

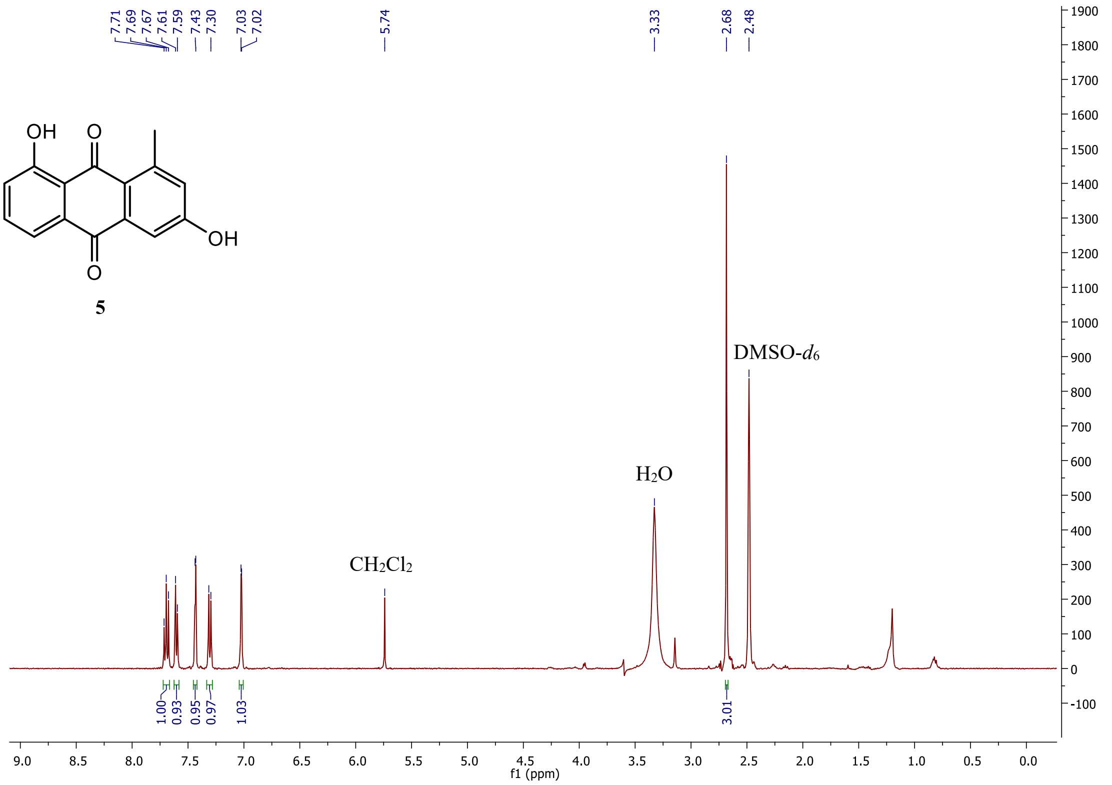

Figure S9: ^{13}C NMR (100 MHz, DMSO-d_6) spectrum of compound **5**

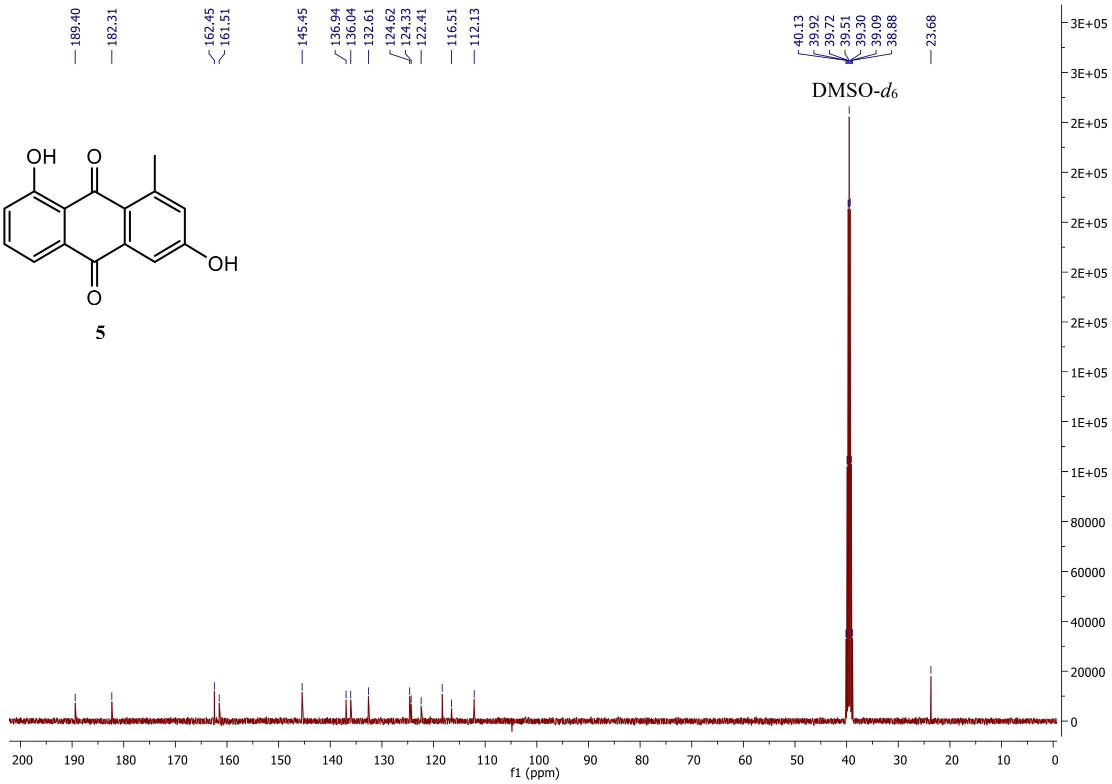

Figure S10: ^{1}H NMR (400 MHz, $CDCl_3$ + MeOH-d_4) spectrum of compound **6**

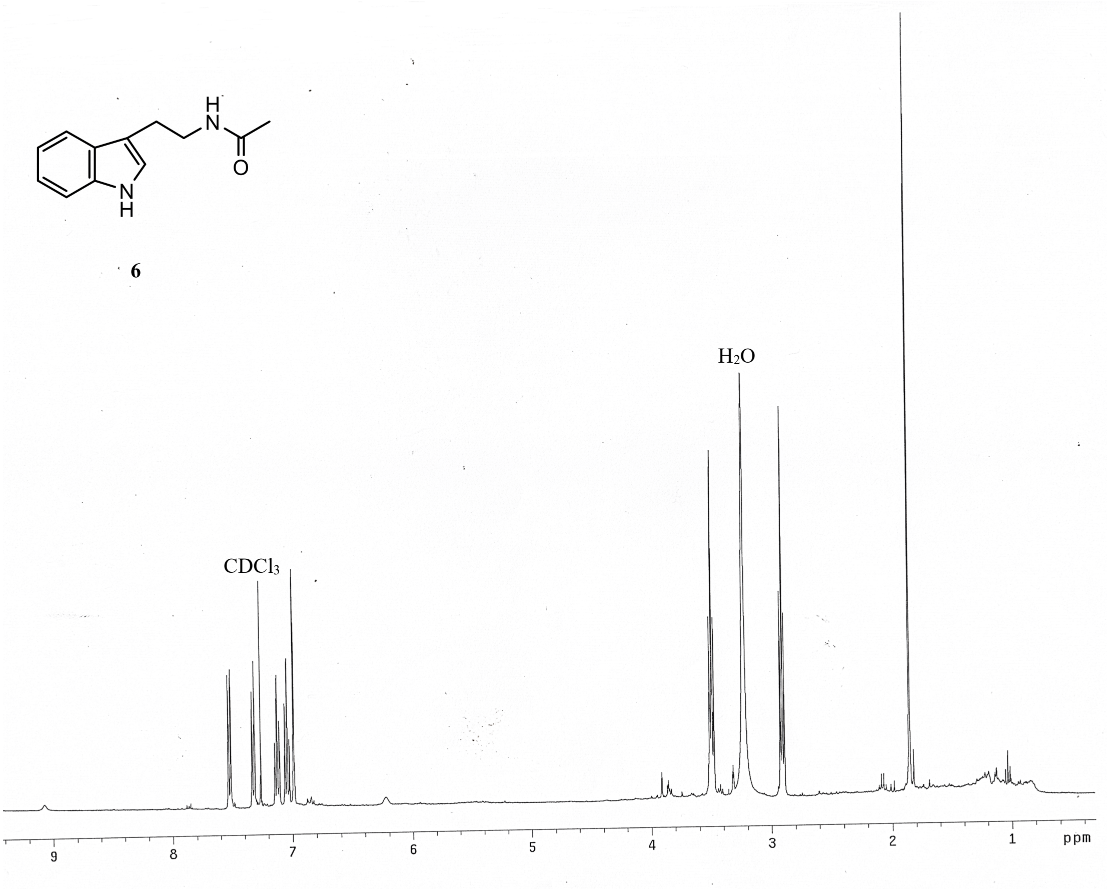

Figure S11: ^{13}C NMR (100 MHz, $CDCl_3$ + MeOH-d_4) spectrum of compound **6**

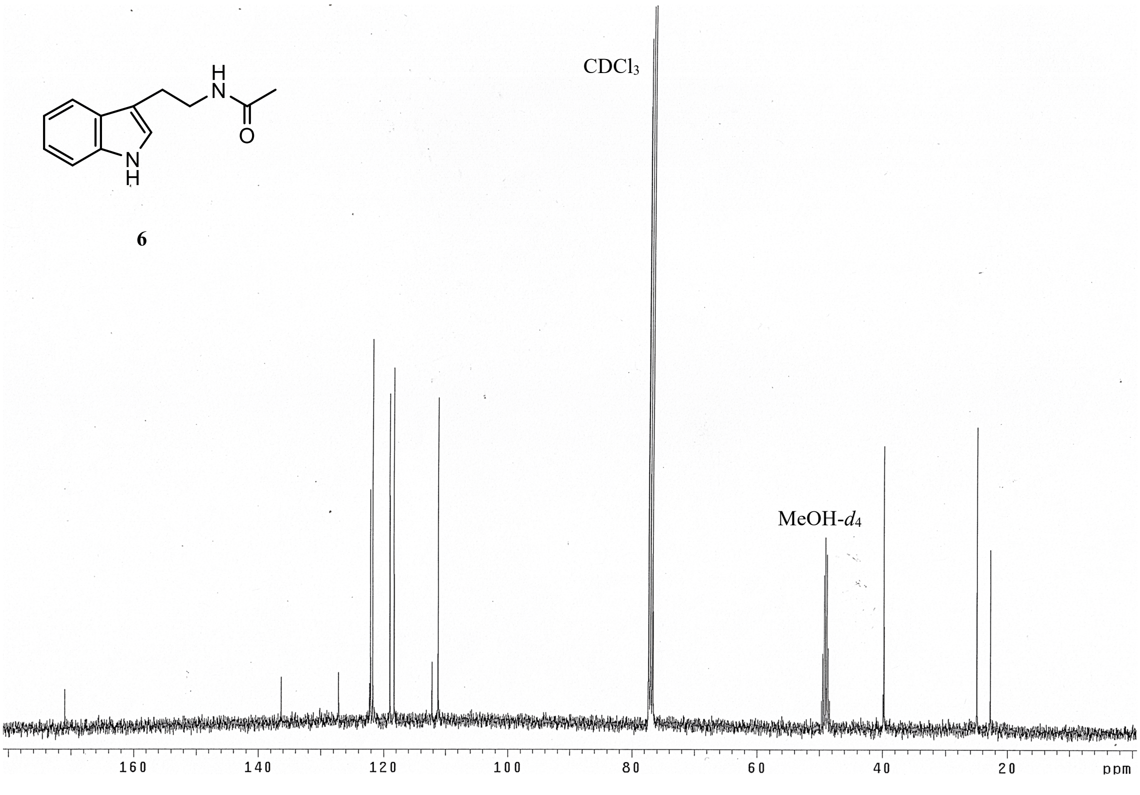

Figure S12: ^{1}H NMR (400 MHz, MeOH-d_4) spectrum of compound **7**

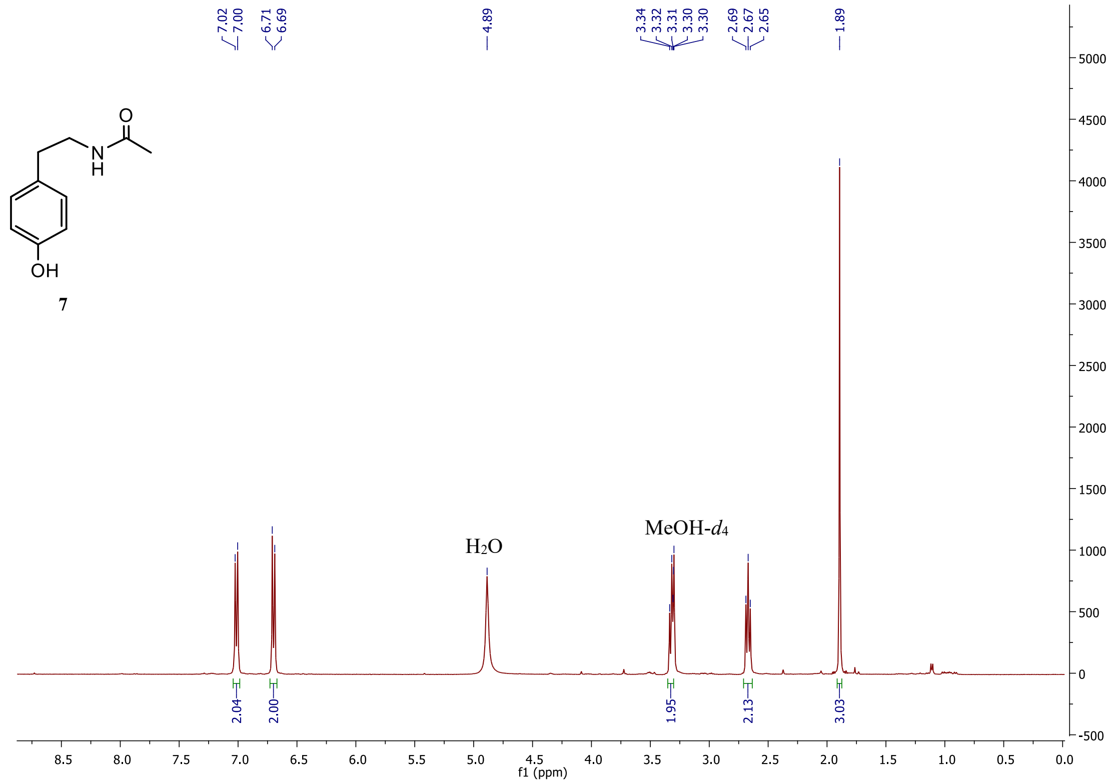

Figure S13: ^{13}C NMR (100 MHz, MeOH-d_4) spectrum of compound **7**

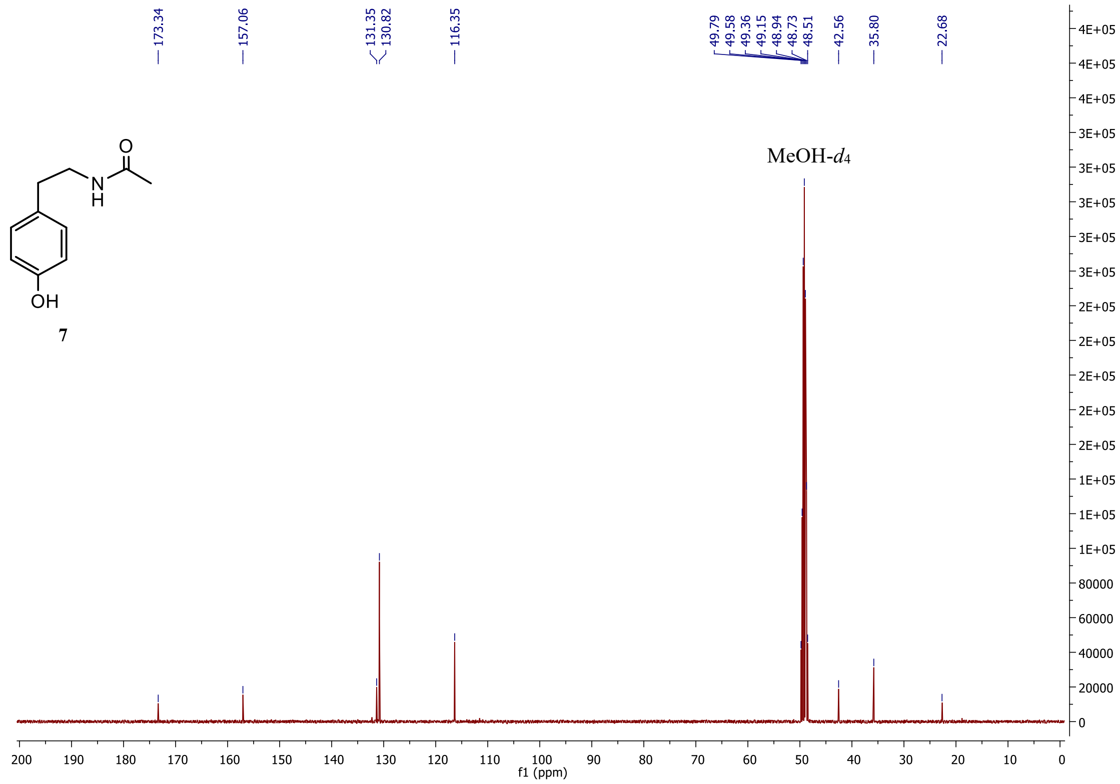

Figure S14: ^{1}H NMR (400 MHz, MeOH-d_4) spectrum of compound **8**

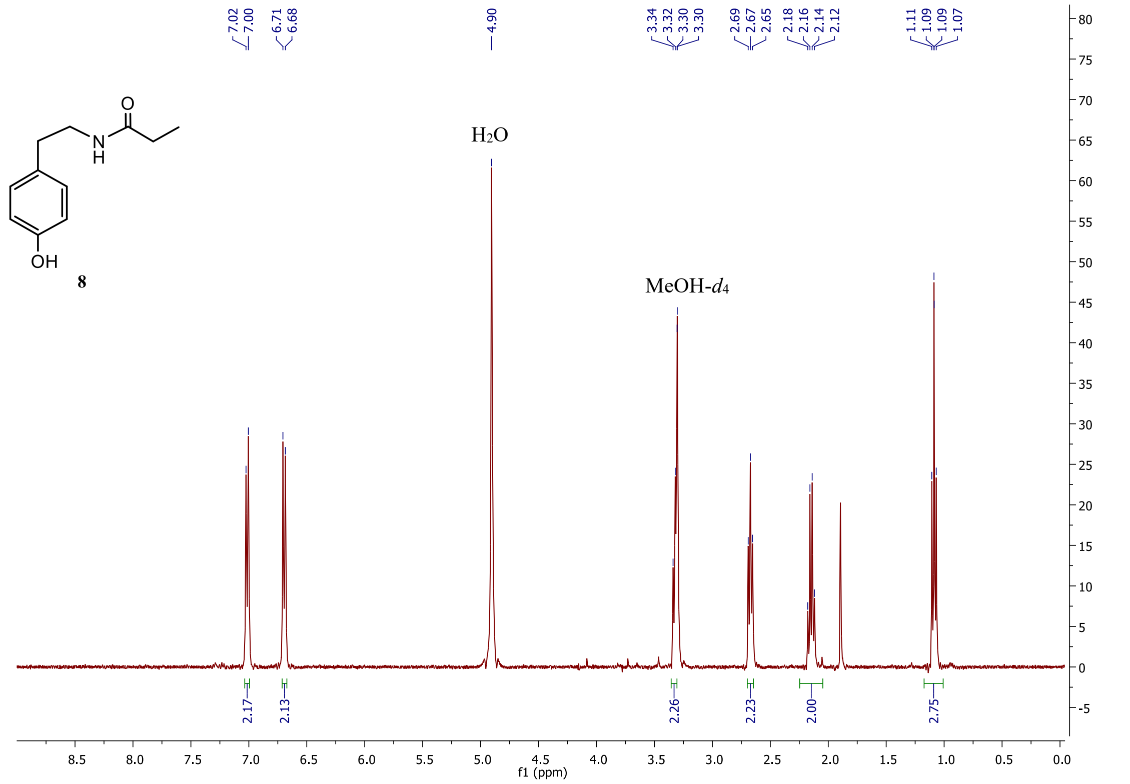

Figure S15: ^{13}C NMR (100 MHz, MeOH-d_4) spectrum of compound **8**

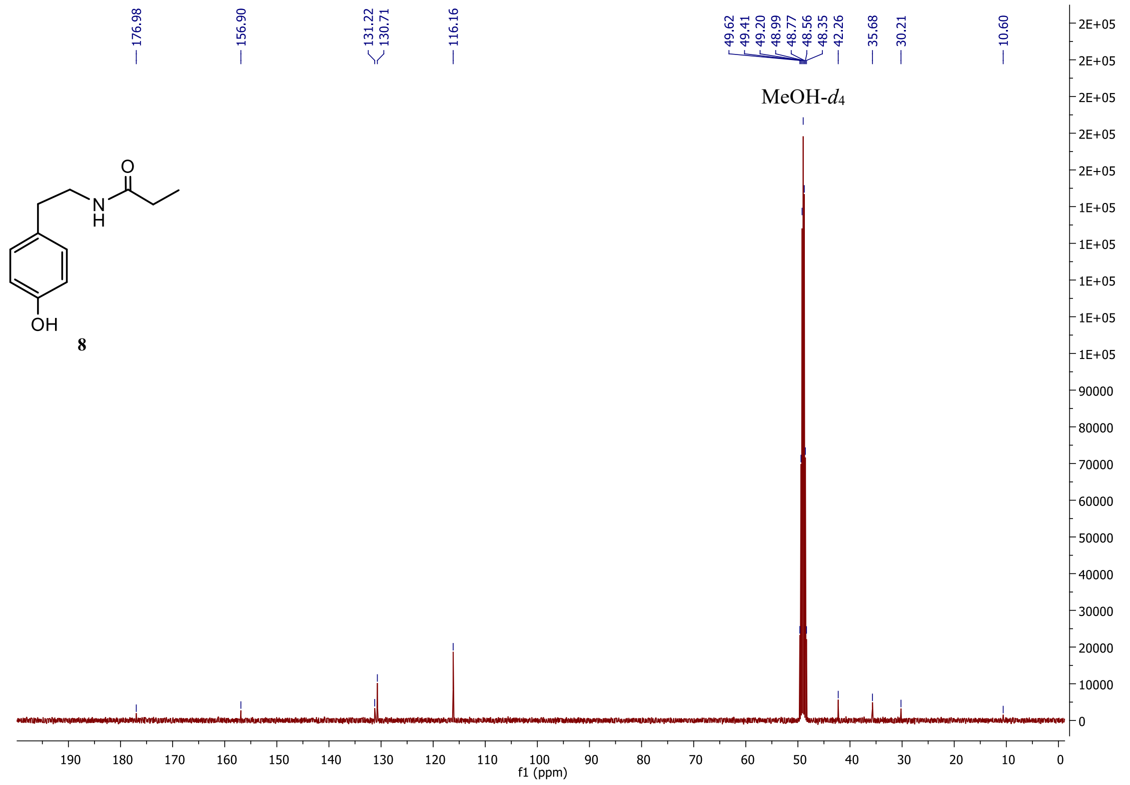

Figure S16: ^{1}H NMR (400 MHz, $CDCl_3$ + MeOH-d_4) spectrum of compound **9**

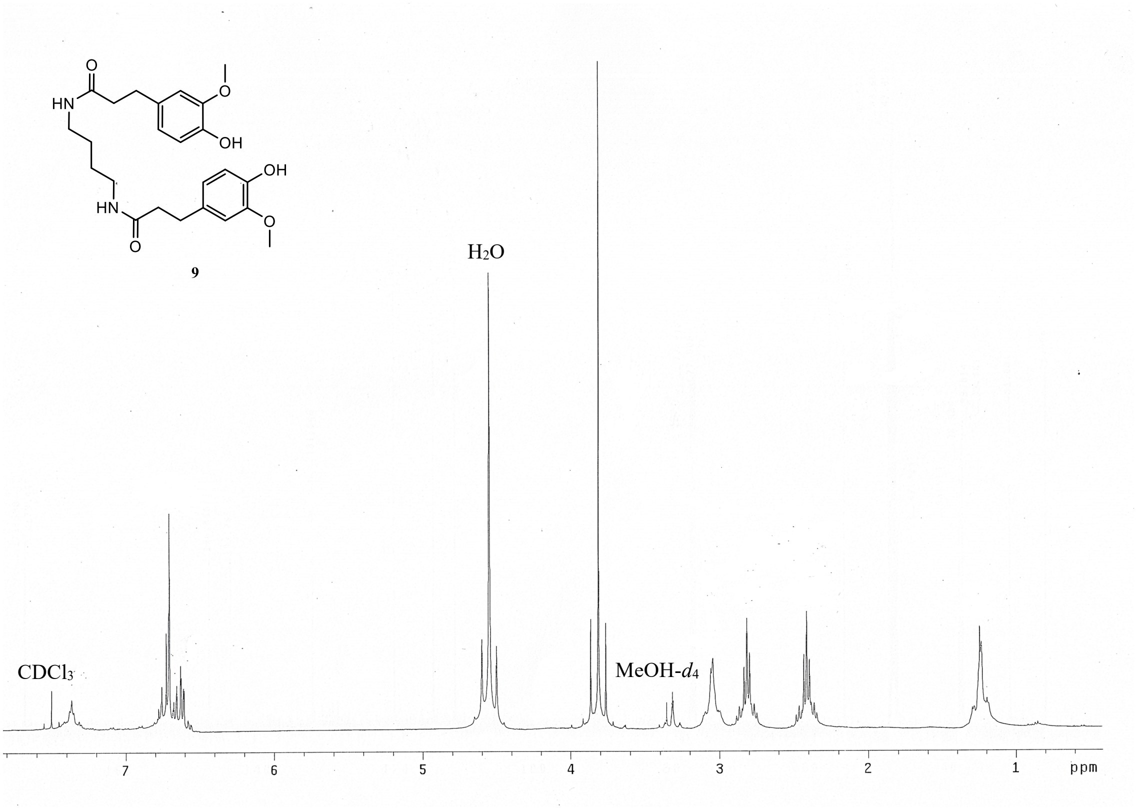

Figure S17: ^{13}C NMR (100 MHz, $CDCl_3$ + MeOH-d_4) spectrum of compound **9**

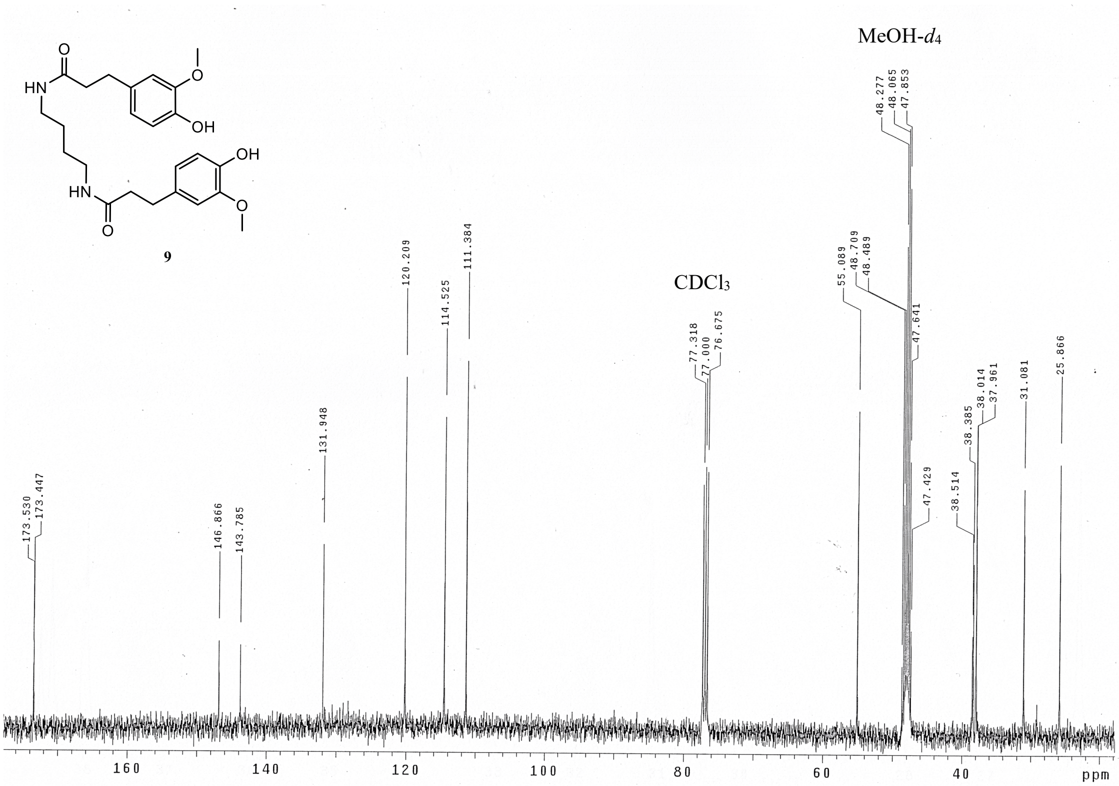

Figure S18: ^{1}H NMR (400 MHz, $CDCl_3$ + MeOH-d_4) spectrum of compound **10**

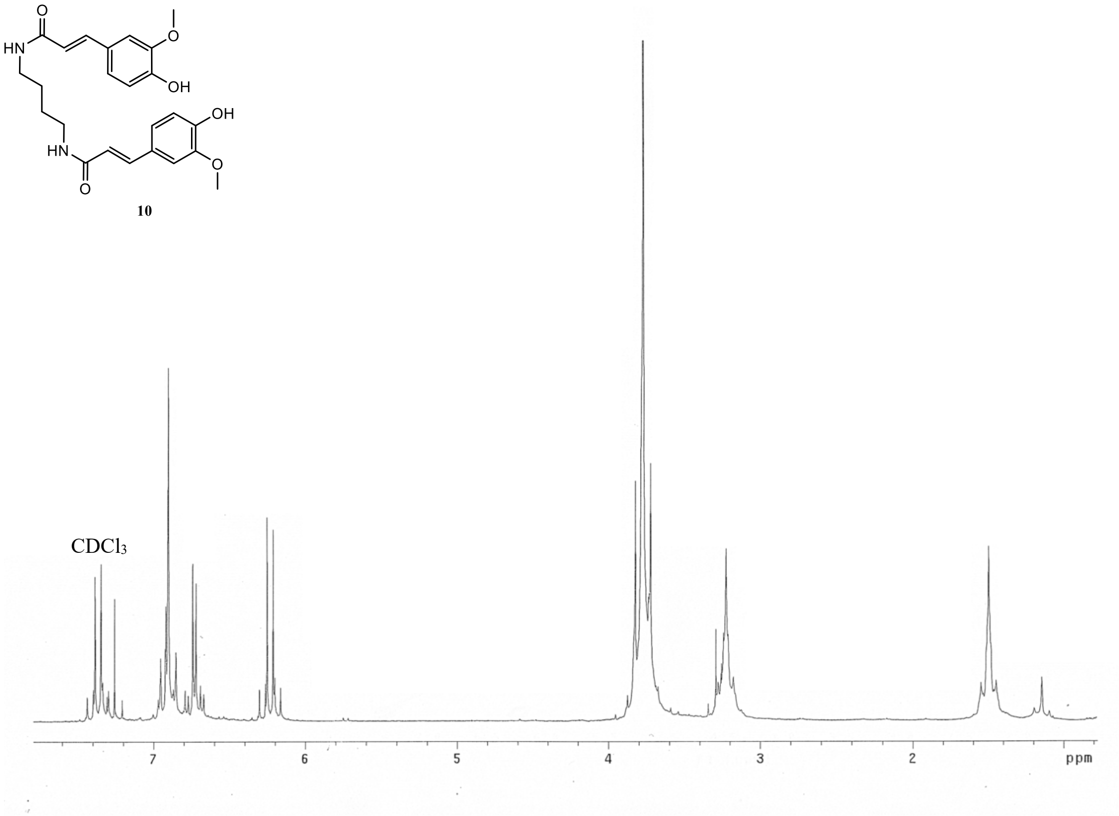

Figure S19: ^{13}C NMR (100 MHz, $CDCl_3$ + MeOH-d_4) spectrum of compound **10**

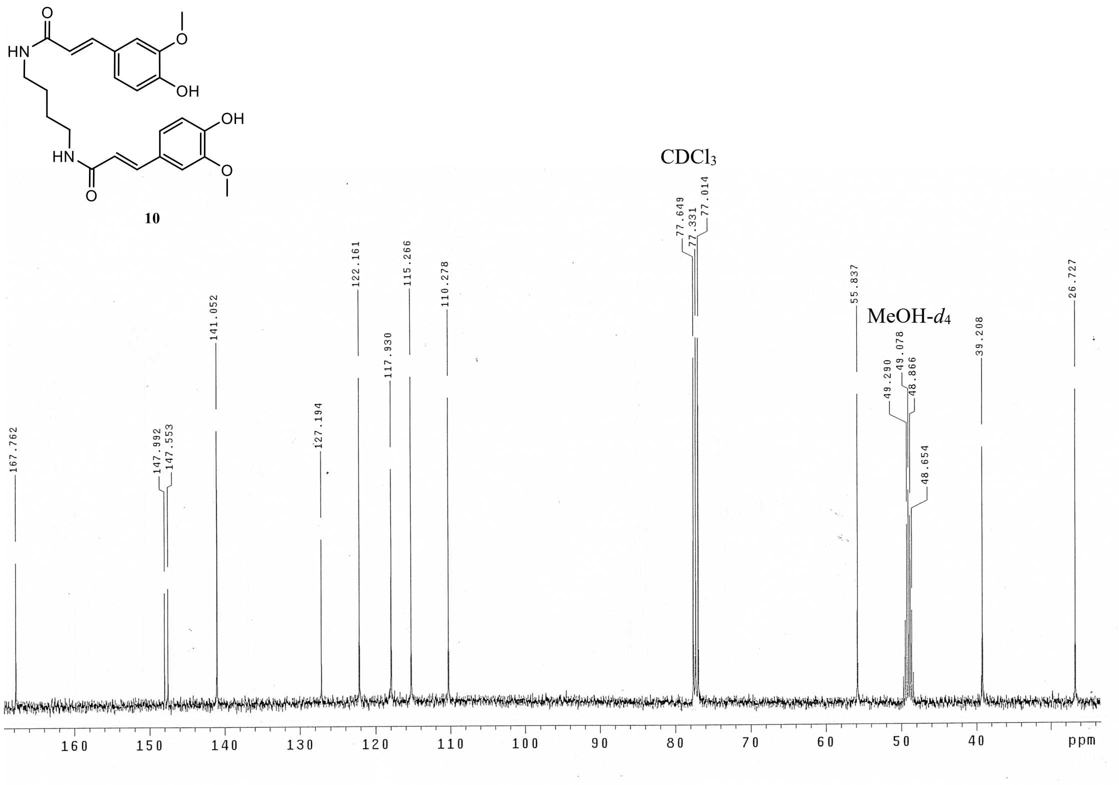

Figure S20: ^{1}H NMR (400 MHz, $CDCl_3$) spectrum of compound **11**

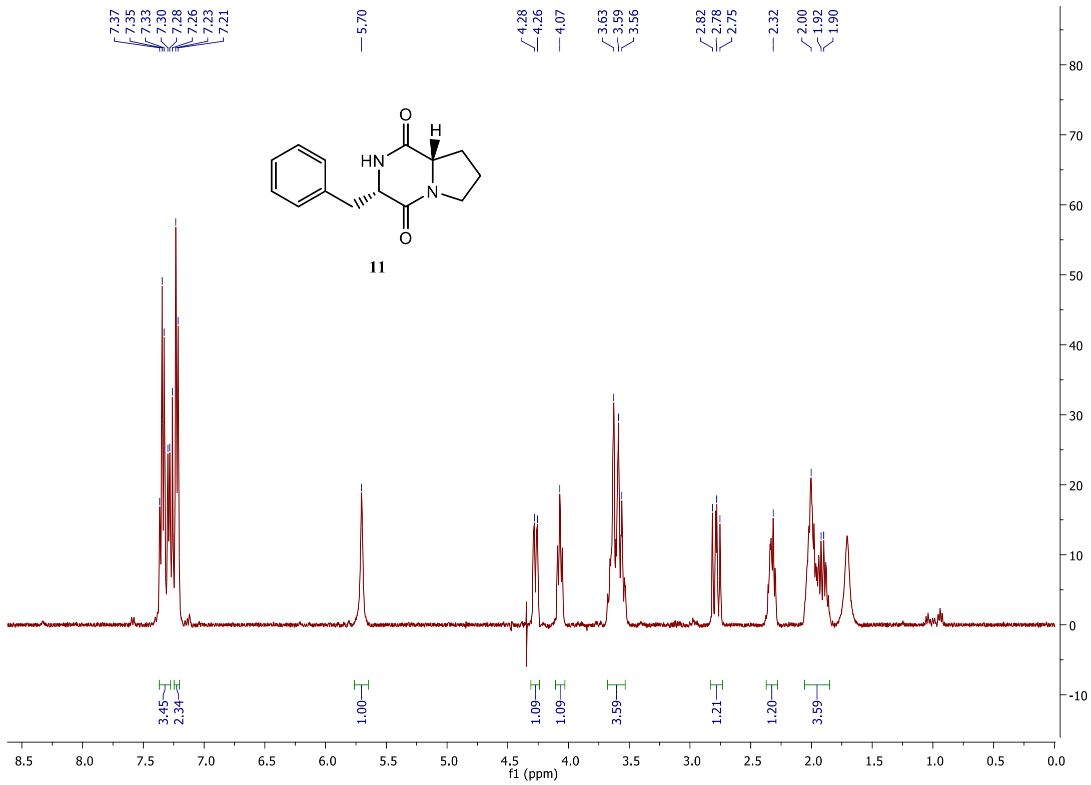

Figure S21: ^{13}C NMR (100 MHz, $CDCl_3$) spectrum of compound **11**

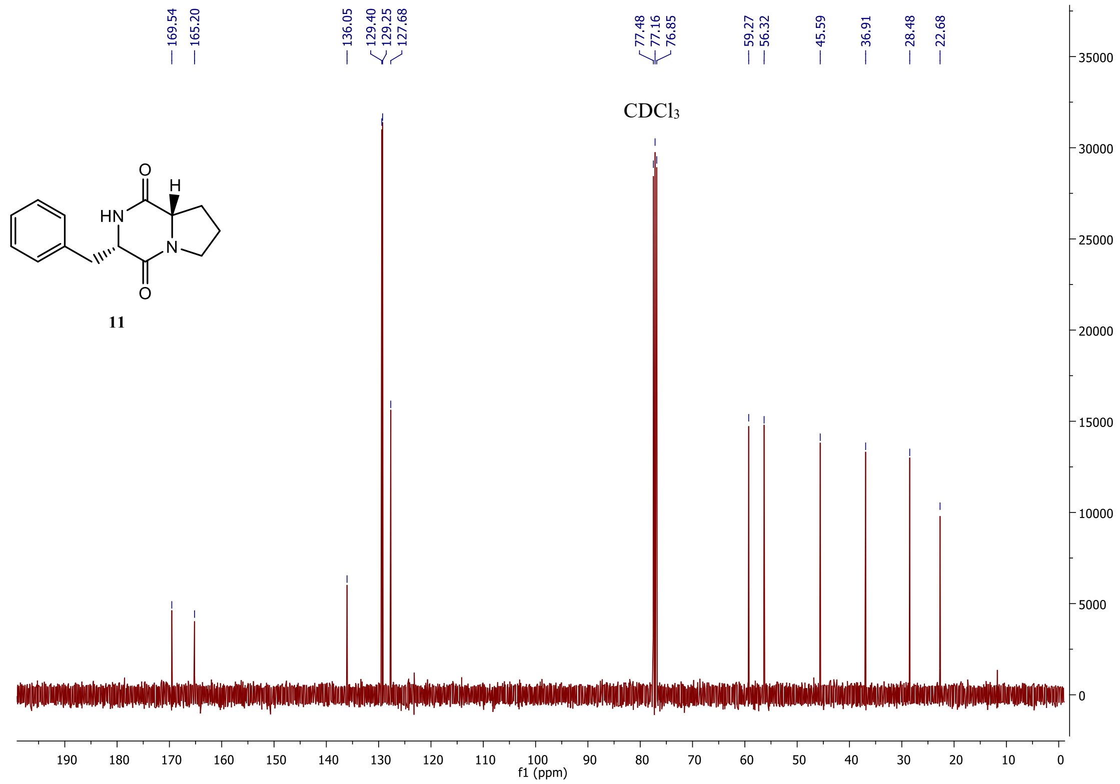

Figure S22: ^{1}H NMR (400 MHz, $CDCl_3$) spectrum of compound **12**

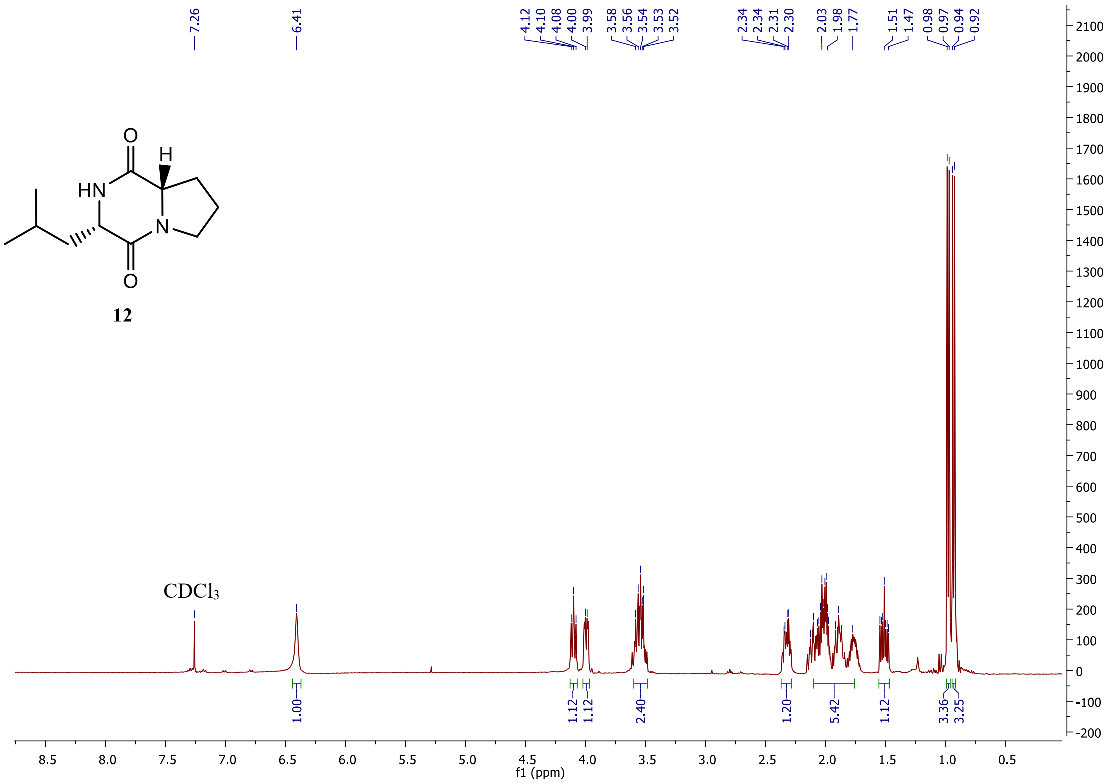

12

Figure S23: ^{13}C NMR (100 MHz, $CDCl_3$) spectrum of compound **12**

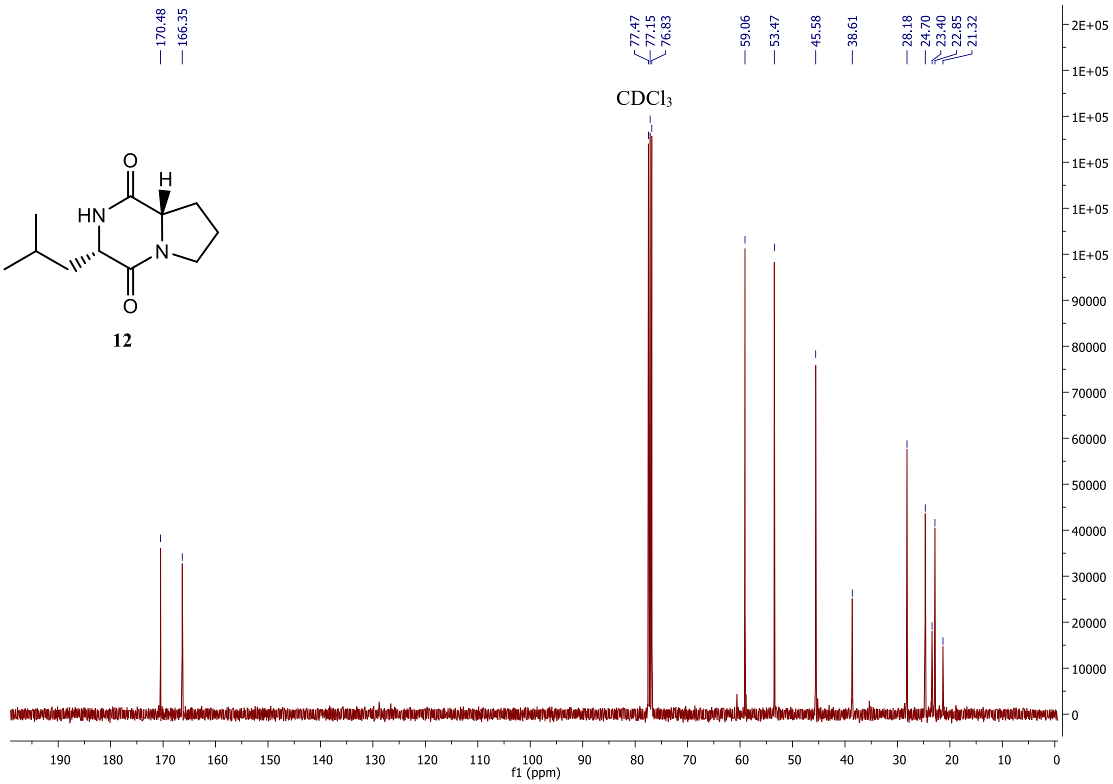

12

Figure S24: ^{1}H NMR (400 MHz, $CDCl_3$) spectrum of compound **13**

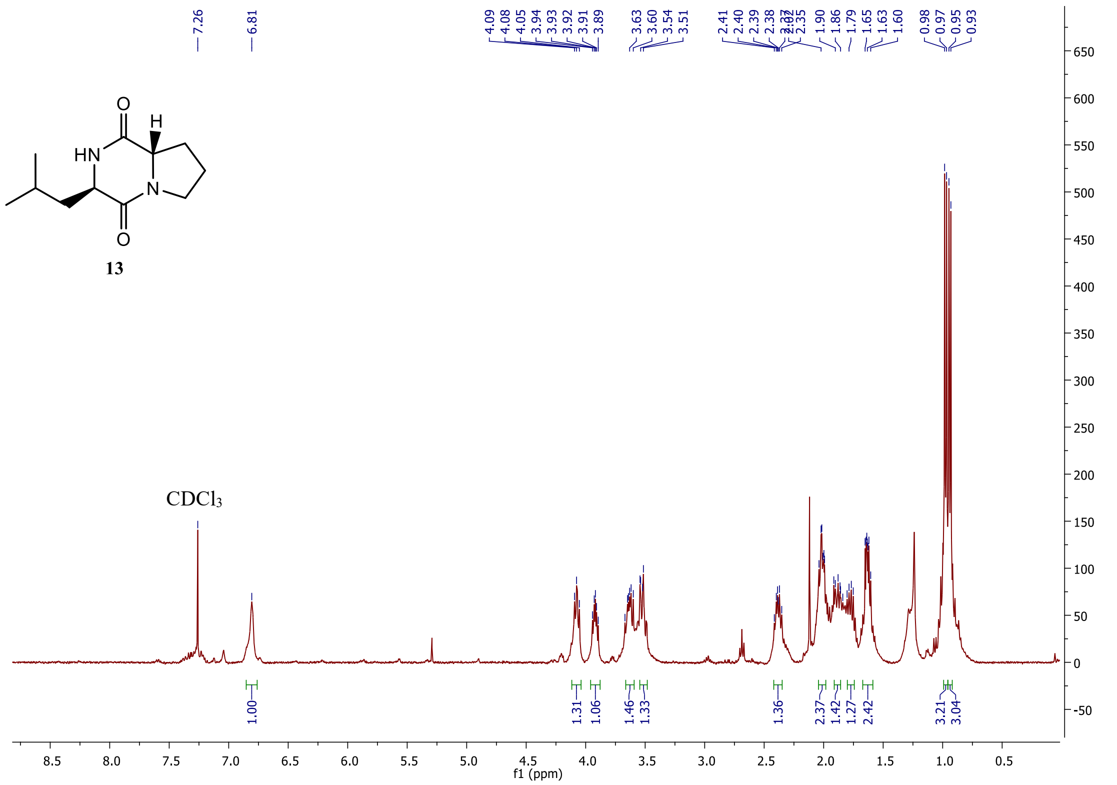

Figure S25: ^{13}C NMR (100 MHz, $CDCl_3$) spectrum of compound **13**

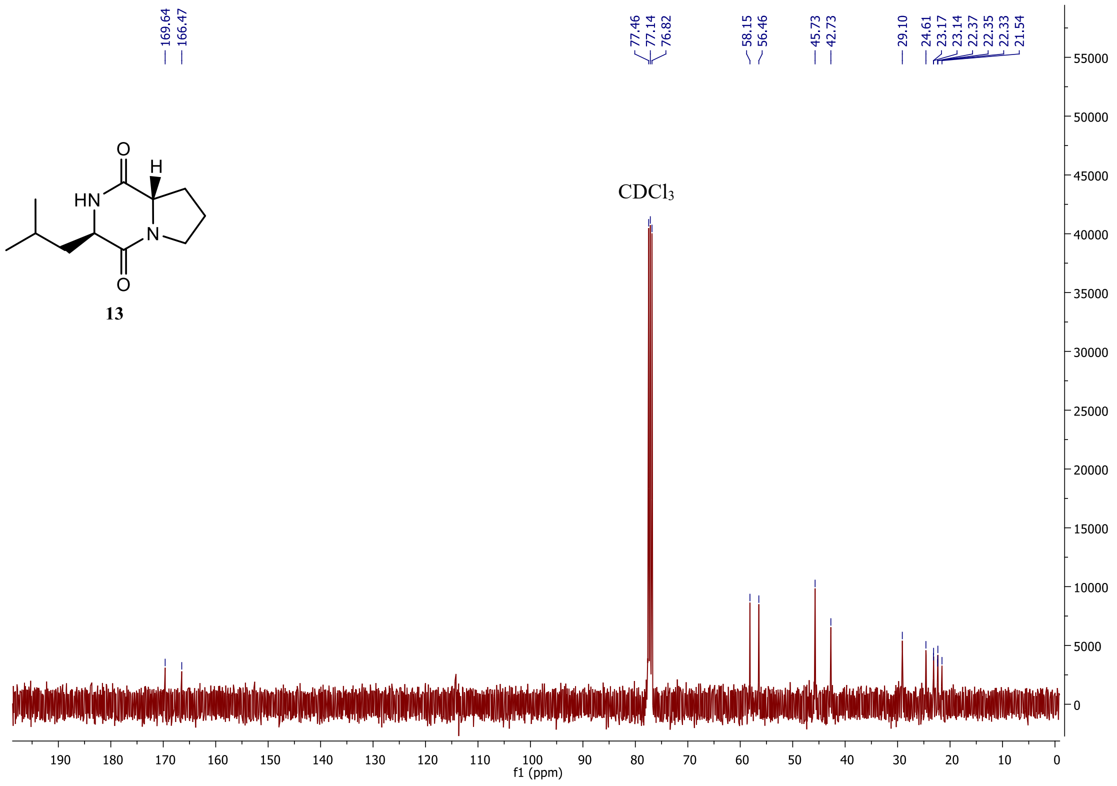

Figure S26: ^{1}H NMR (400 MHz, MeOH-d_4) spectrum of compound **14**

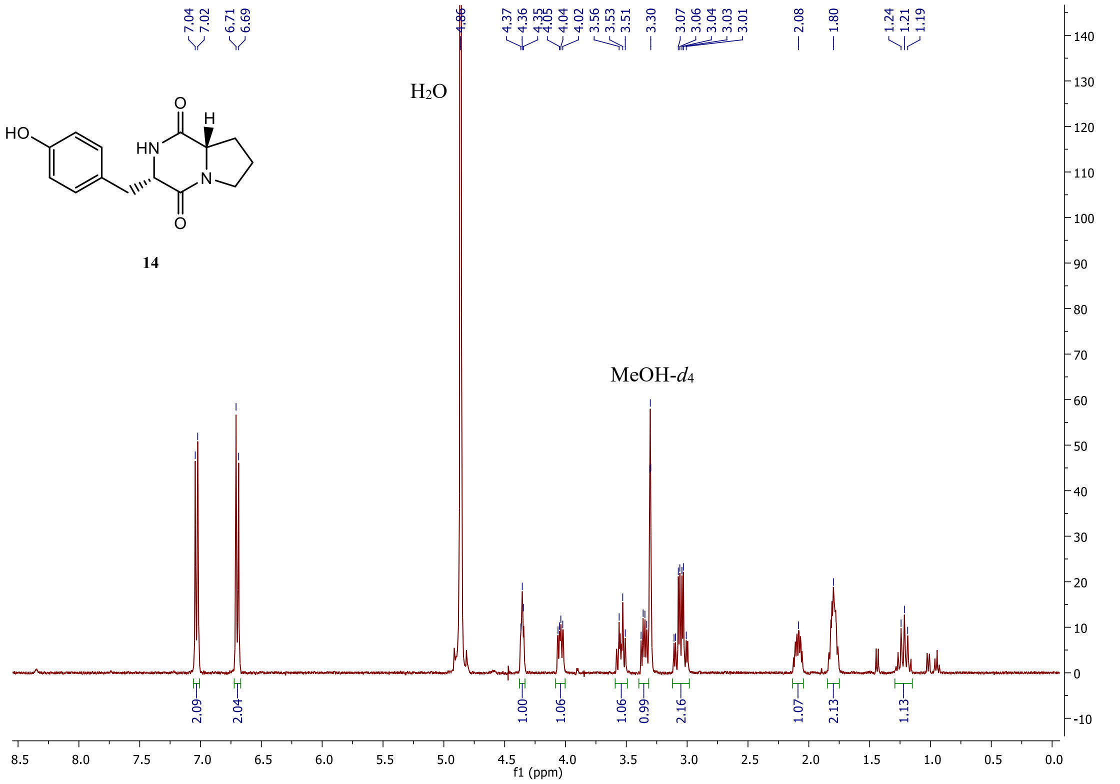

Figure S27: ^{13}C NMR (100 MHz, MeOH-d_4) spectrum of compound **14**

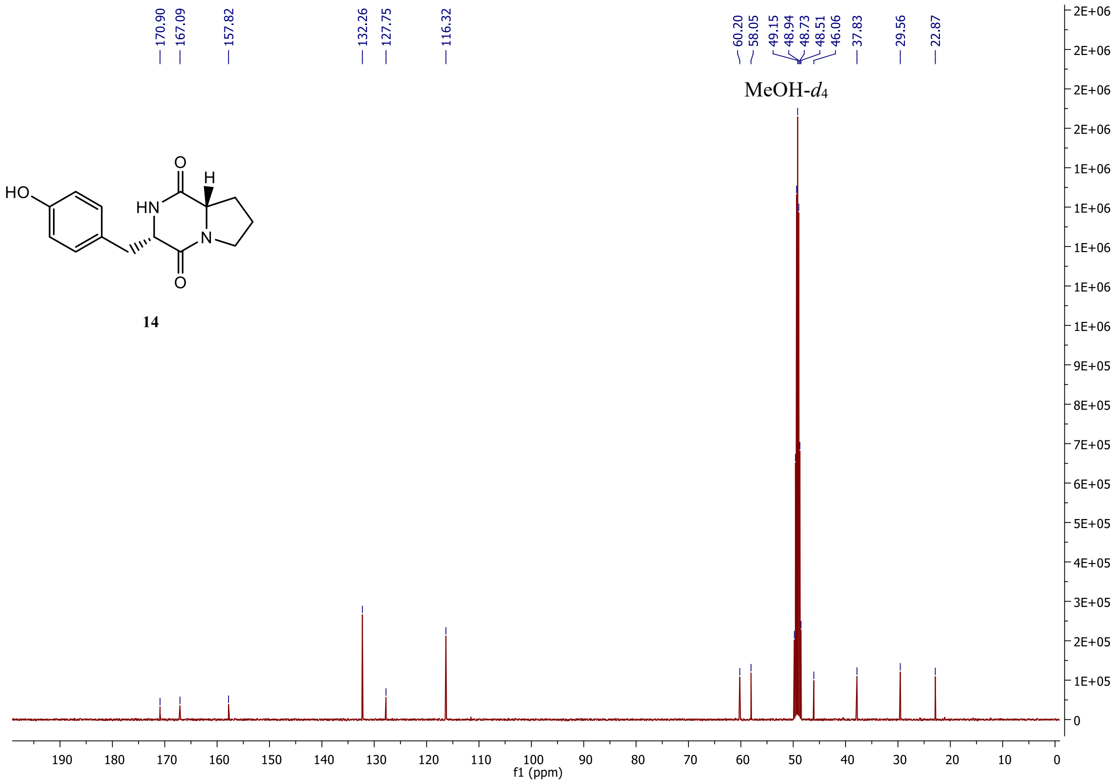

Figure S28: ^{1}H NMR (400 MHz, MeOH-d_4) spectrum of compound **15**

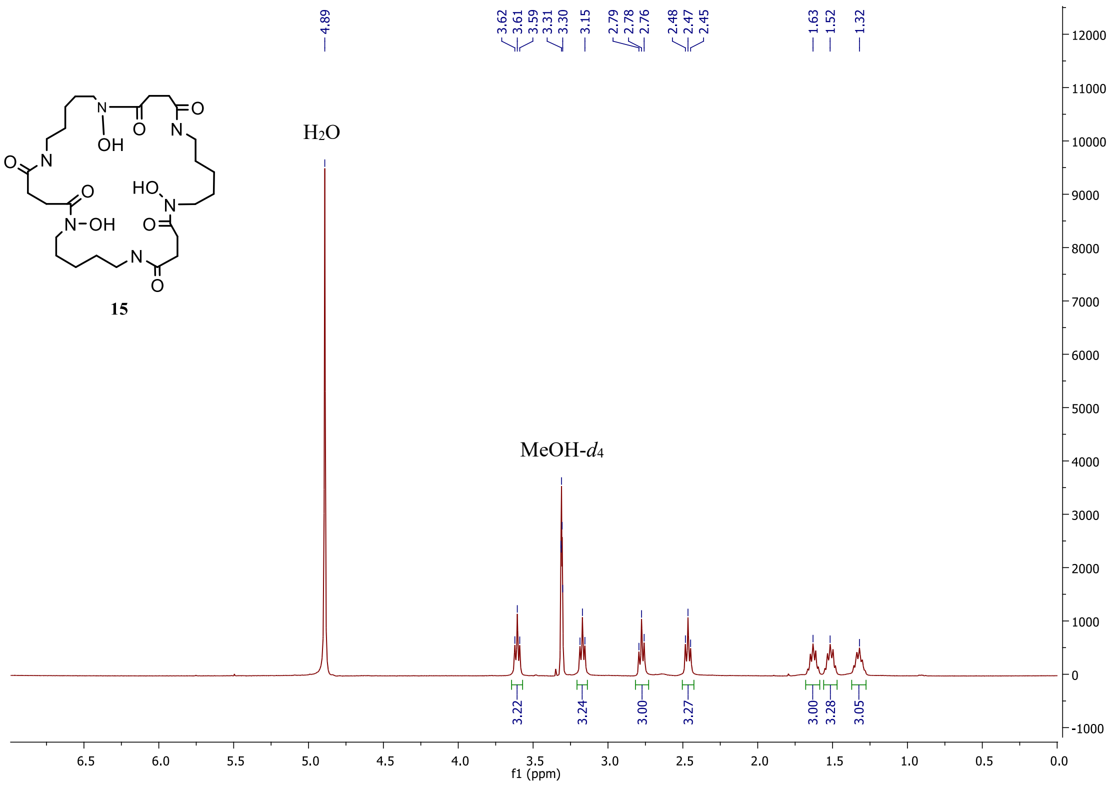

Figure S29: ^{13}C NMR (100 MHz, MeOH-d_4) spectrum of compound **15**

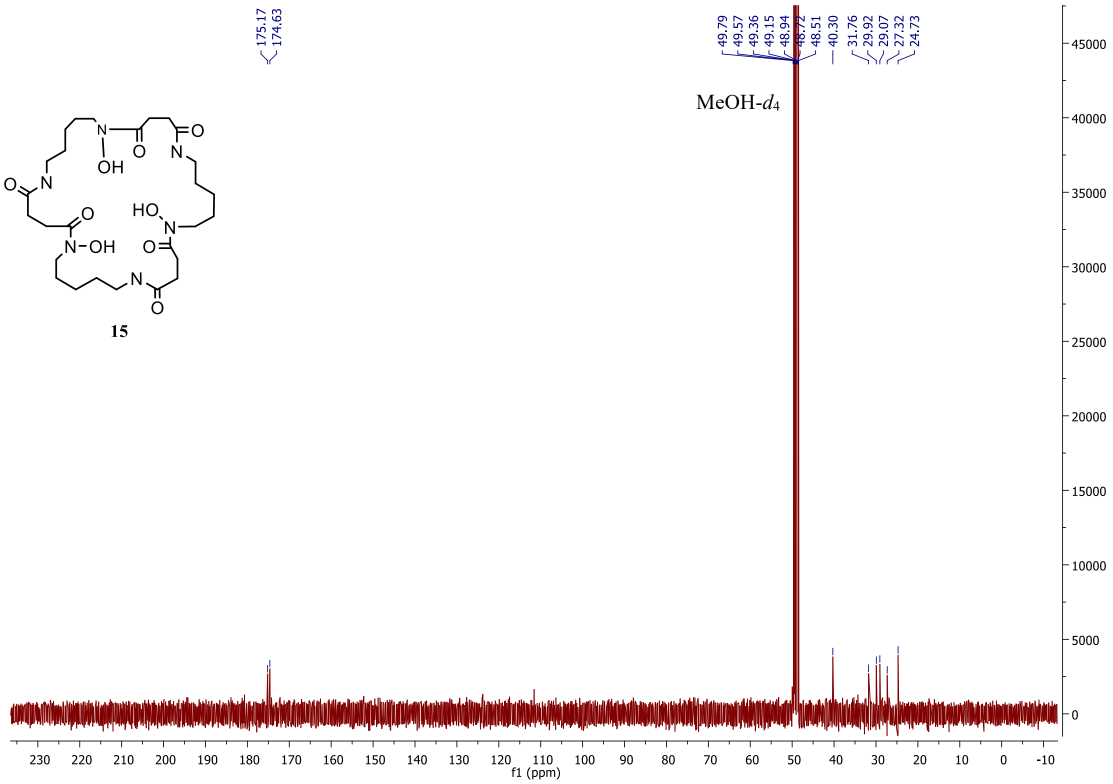

Figure S30: ^{1}H NMR (400 MHz, MeOH-d_4) spectrum of compound **16**

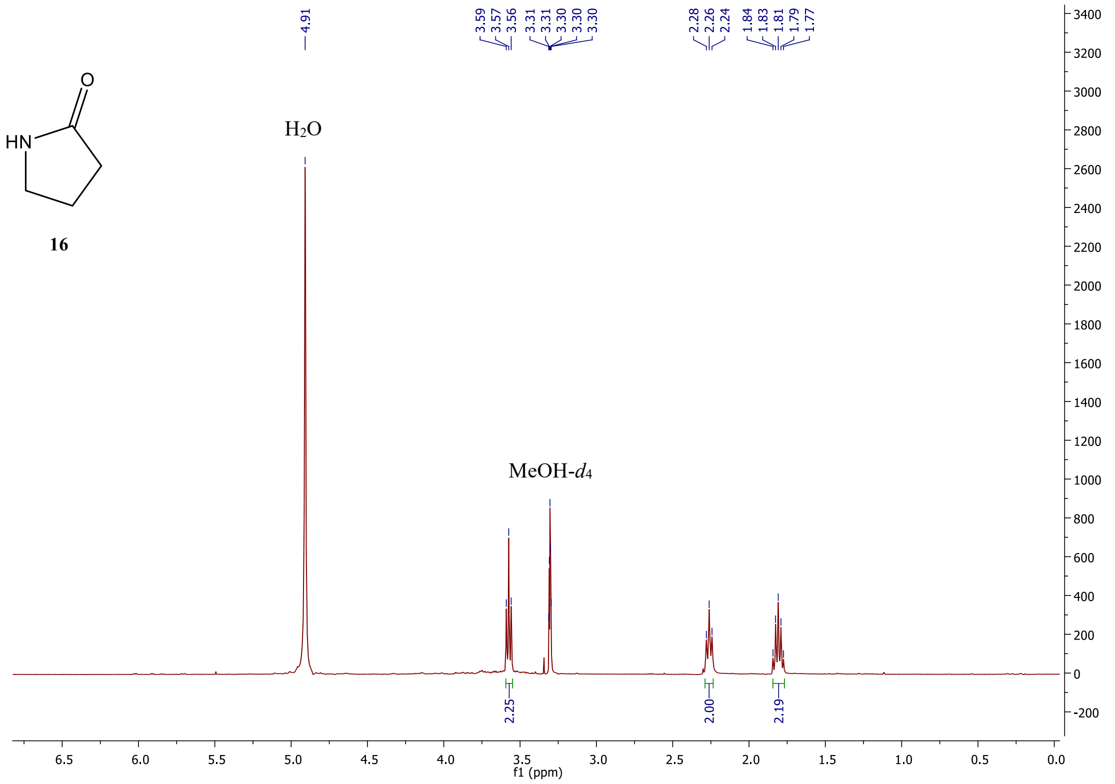

Figure S31: ^{13}C NMR (100 MHz, MeOH-d_4) spectrum of compound **16**

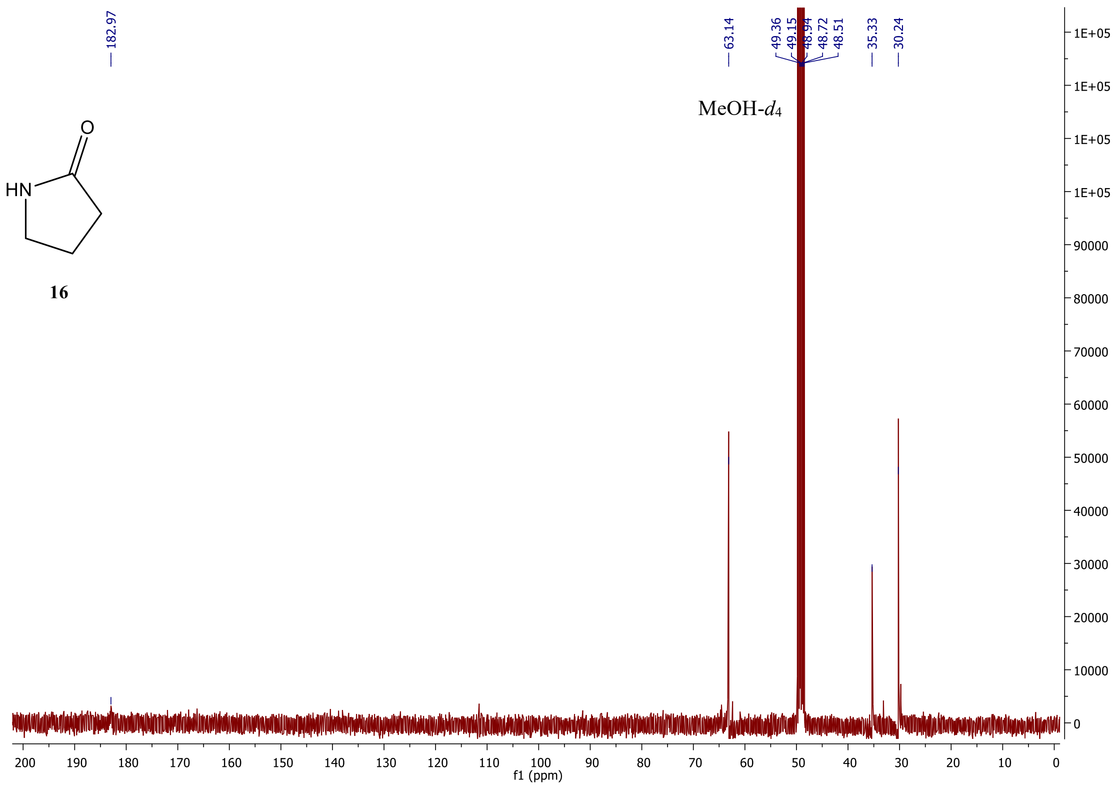

Figure S32: ^{1}H NMR (400 MHz, MeOH-d_4) spectrum of compound **17**

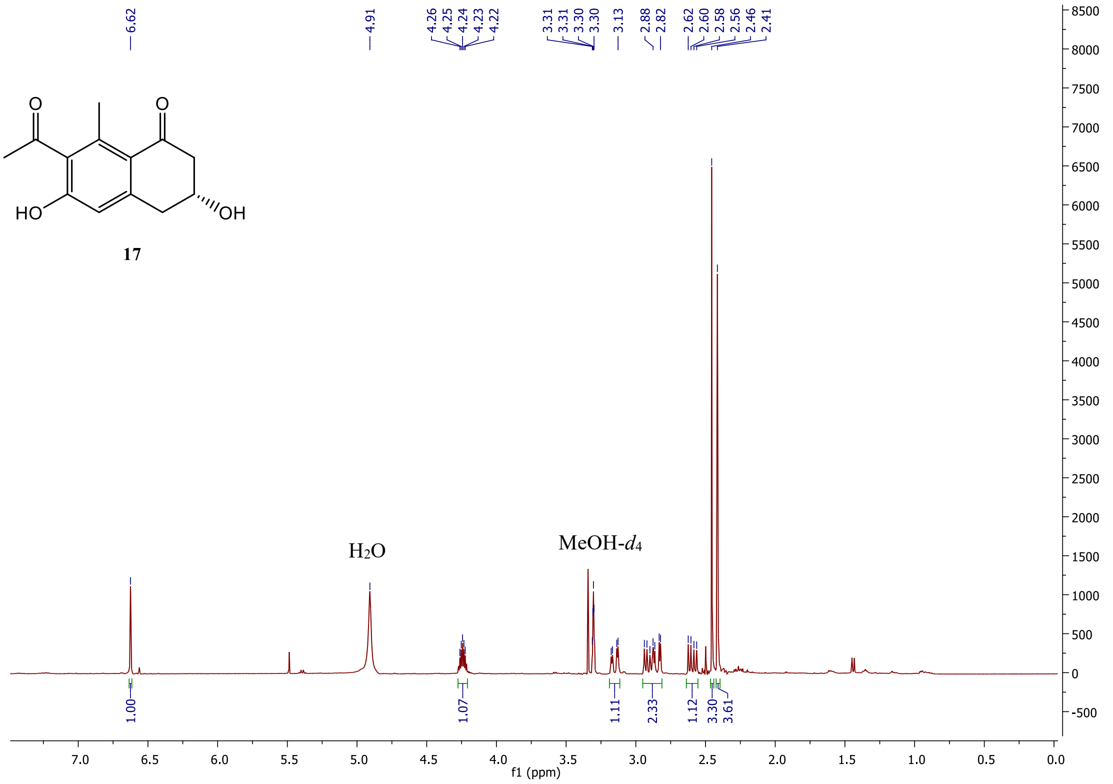

Figure S33: ^{13}C NMR (100 MHz, MeOH-d_4) spectrum of compound **17**

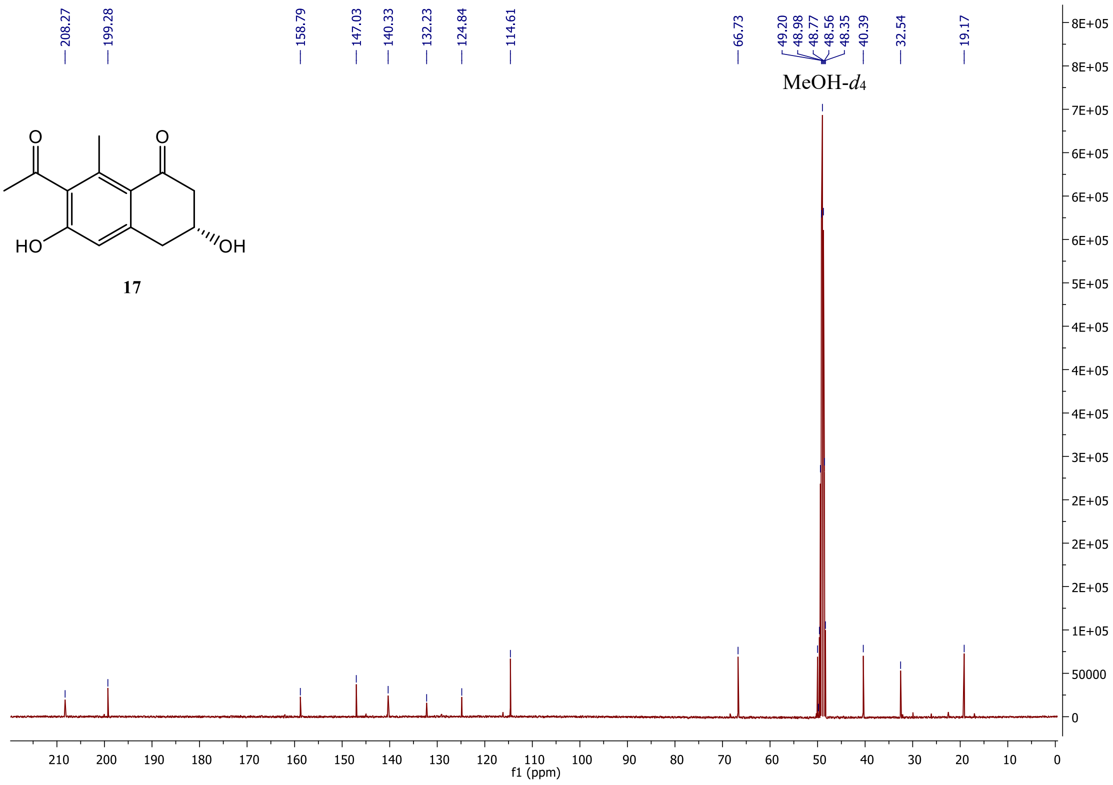

Figure S34: ^{1}H NMR (400 MHz, $CDCl_3$) spectrum of compound **18**

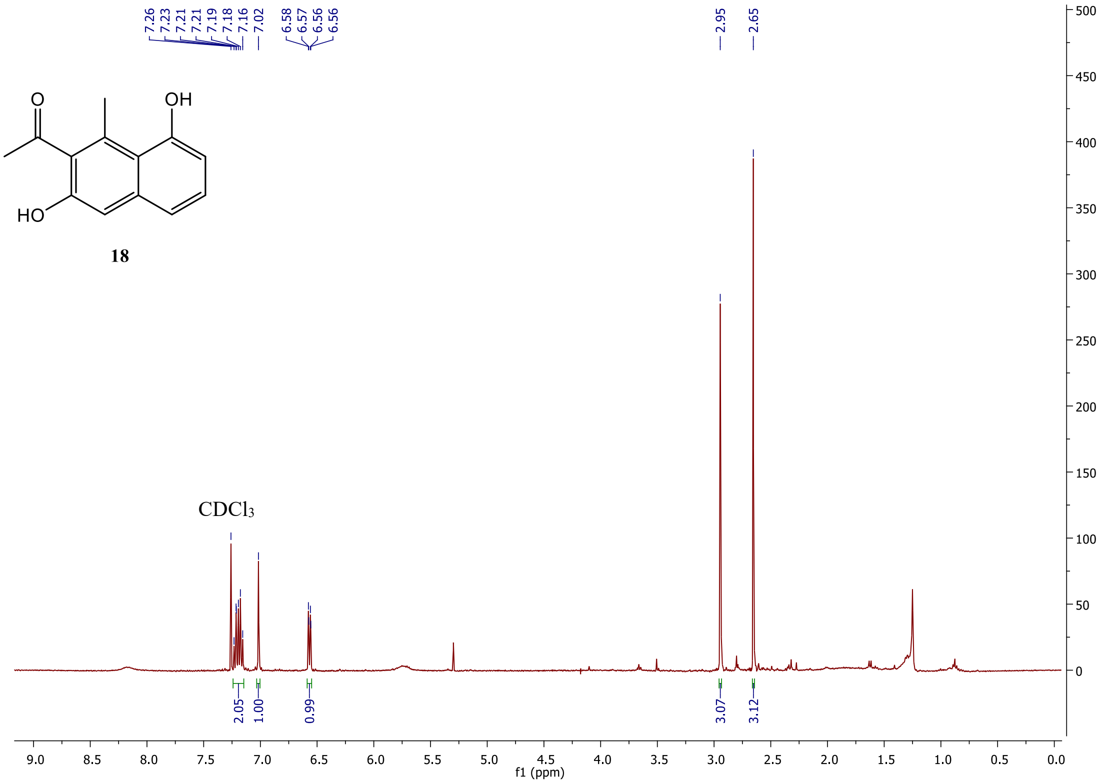

Figure S35: ^{13}C NMR (100 MHz, $CDCl_3$) spectrum of compound **18**

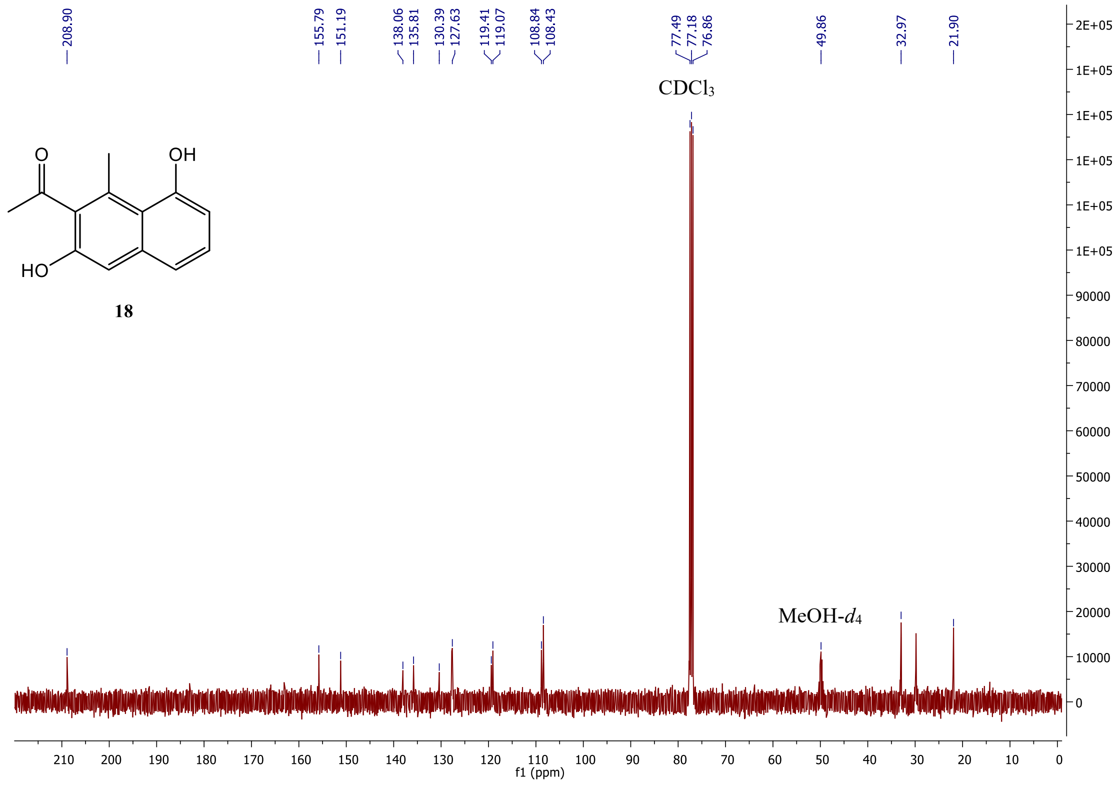

2. Experimental spectra of novel compounds 19-23

Figure S36: HRESIMS spectrum of compound **19**

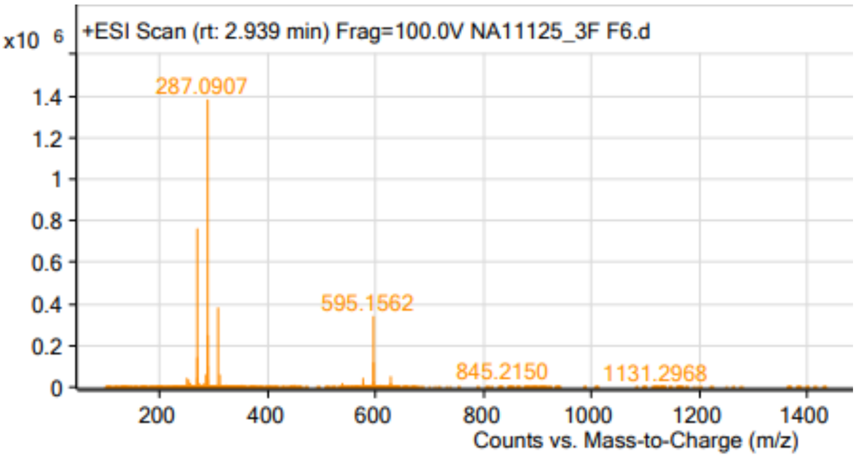

Figure S37: UV absorption spectrum of compound **19**

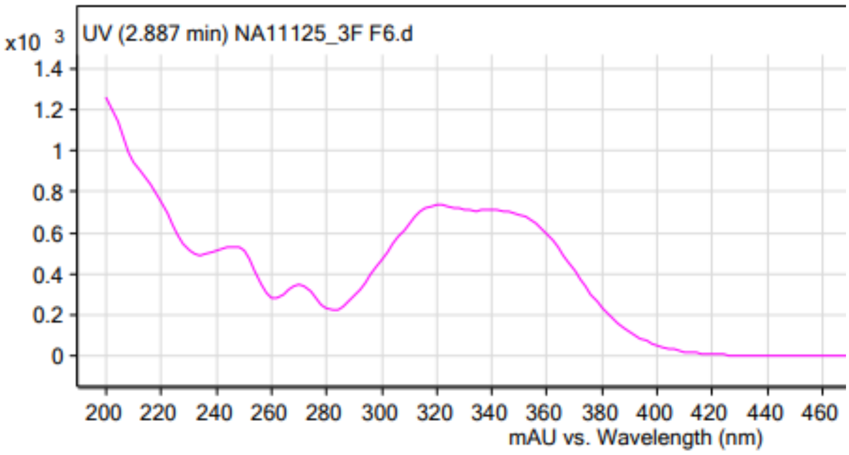

Figure S38: IR spectrum of compound **19**

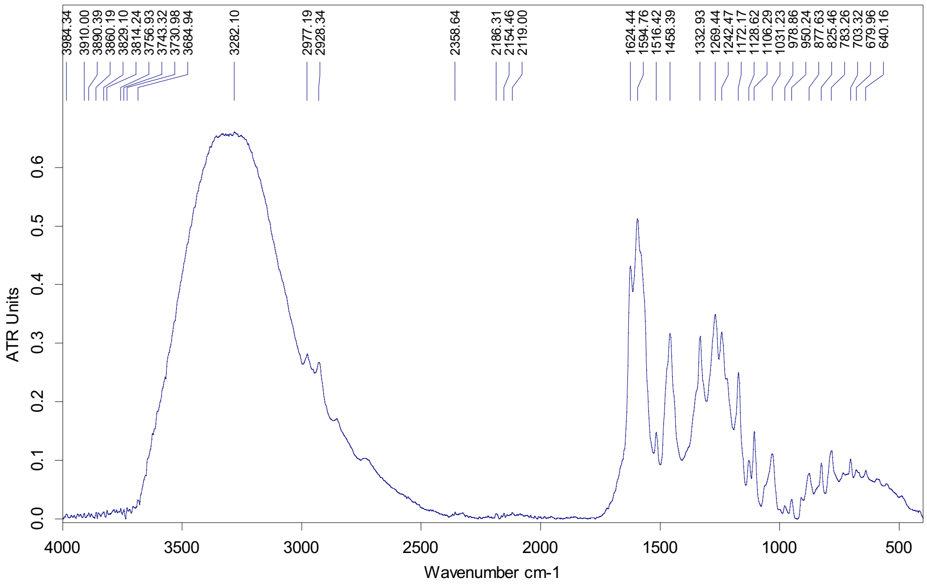

Figure S39: ^{1}H NMR (400 MHz, $CDCl_3$ + MeOH-d_4) spectrum of compound **19**

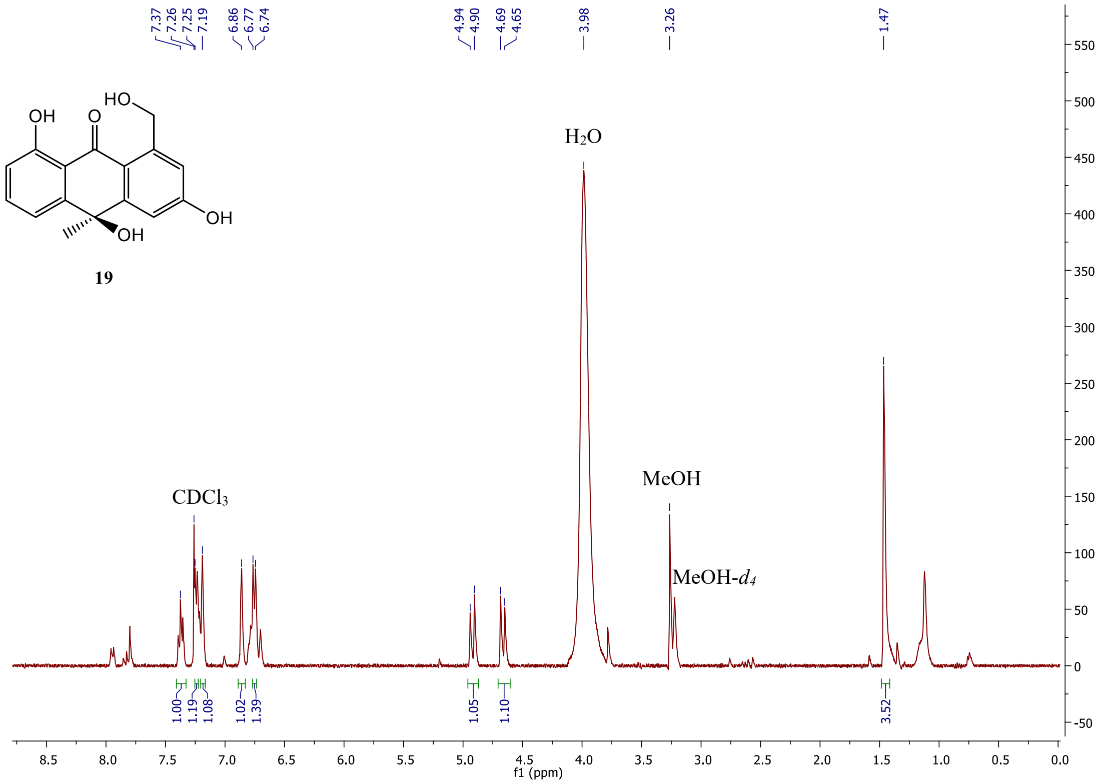

Figure S40: ^{1}H-^{1}H COSY (400 MHz, $CDCl_3$ + MeOH-d_4) spectrum of compound **19**

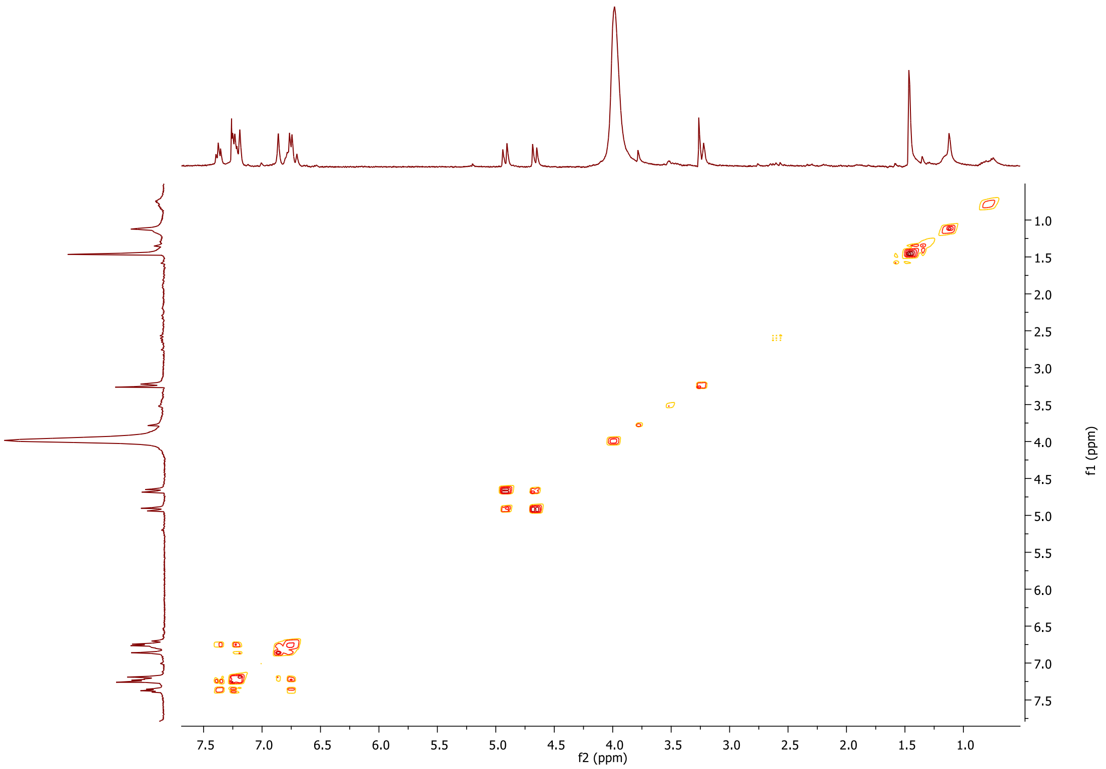

Figure S41: gHSQC (400 MHz and 100MHz, $CDCl_3$ + MeOH-d_4) spectrum of compound **19**

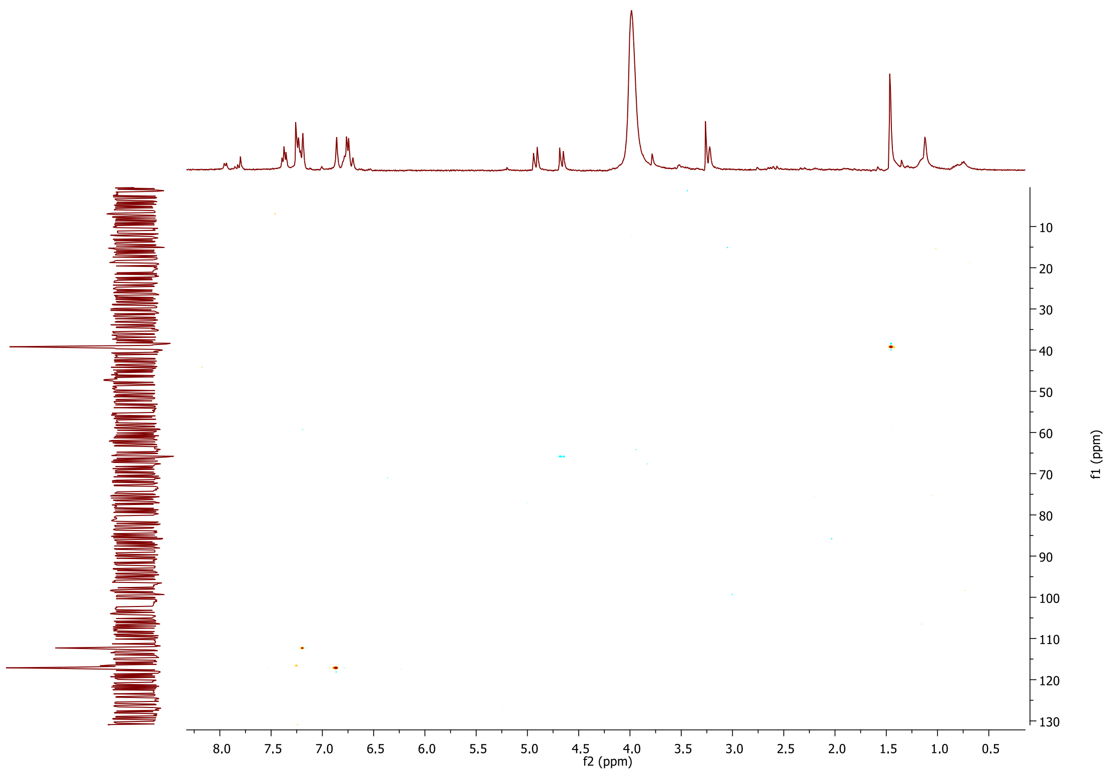

Figure S42: gHMBC (400 MHz and 100MHz, $CDCl_3$ + MeOH-d_4) spectrum of compound **19**

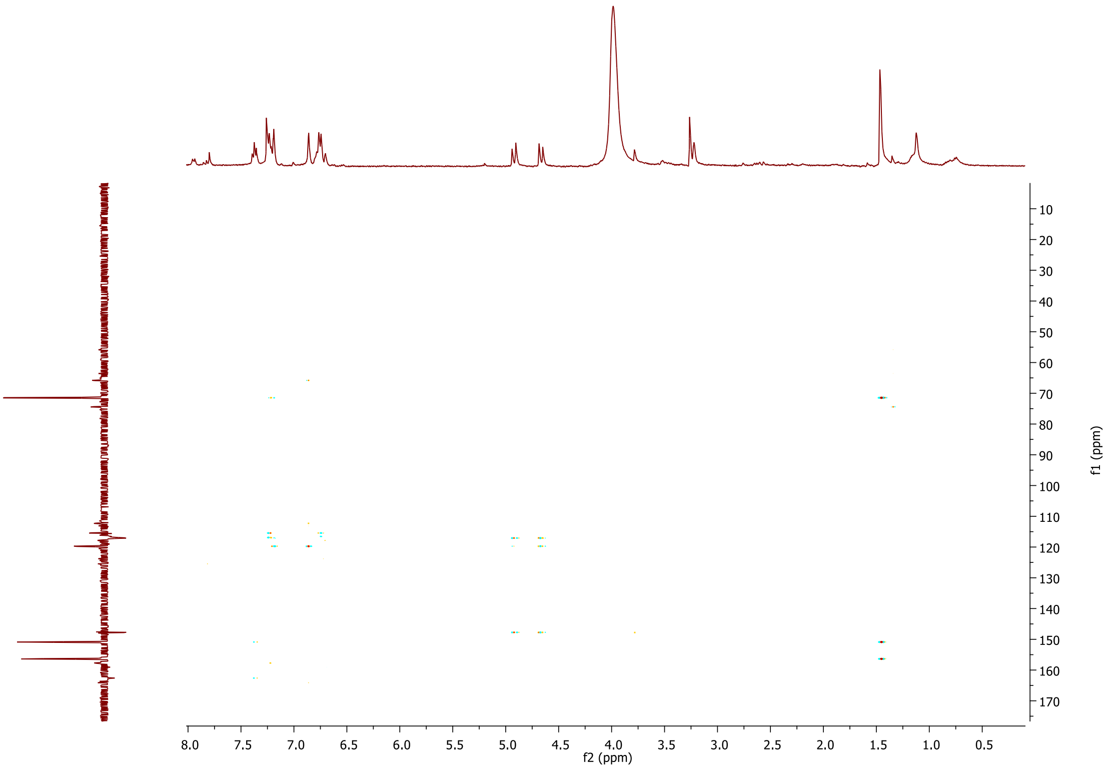

Figure S43: HRESIMS spectrum of compound **20**

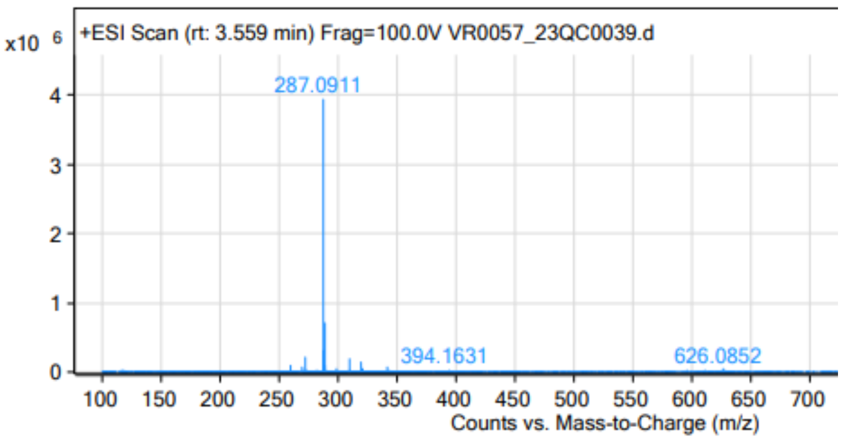

Figure S44: UV absorption spectrum of compound **20**

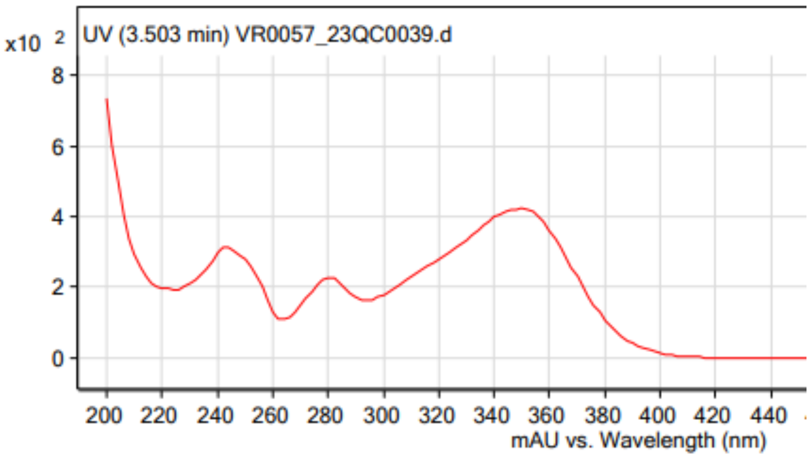

Figure S45: IR spectrum of compound **20**

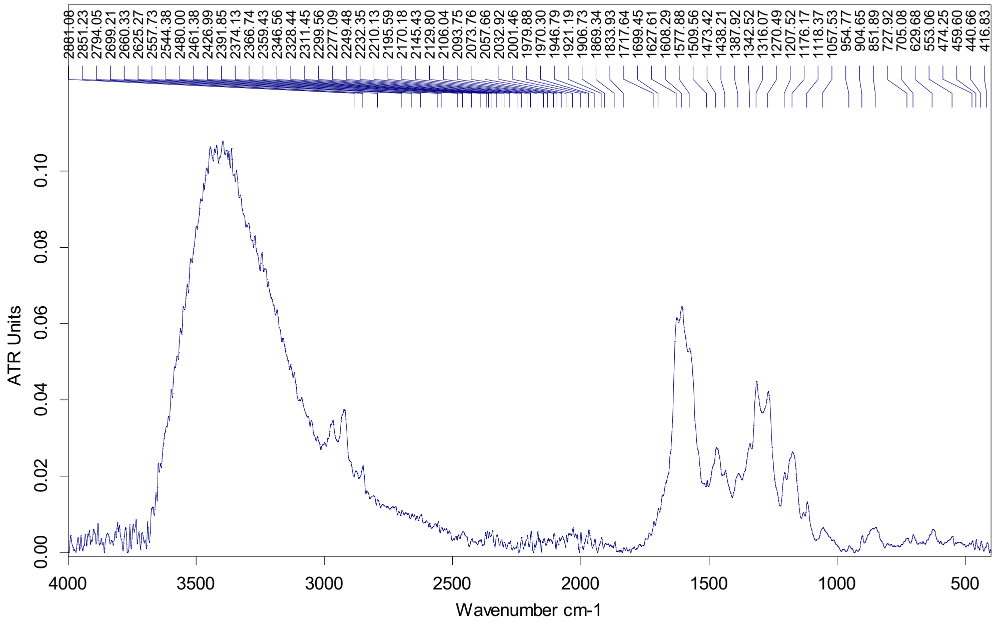

Figure S46: ^{1}H NMR (400 MHz, MeOH-d_4) spectrum of compound **20**

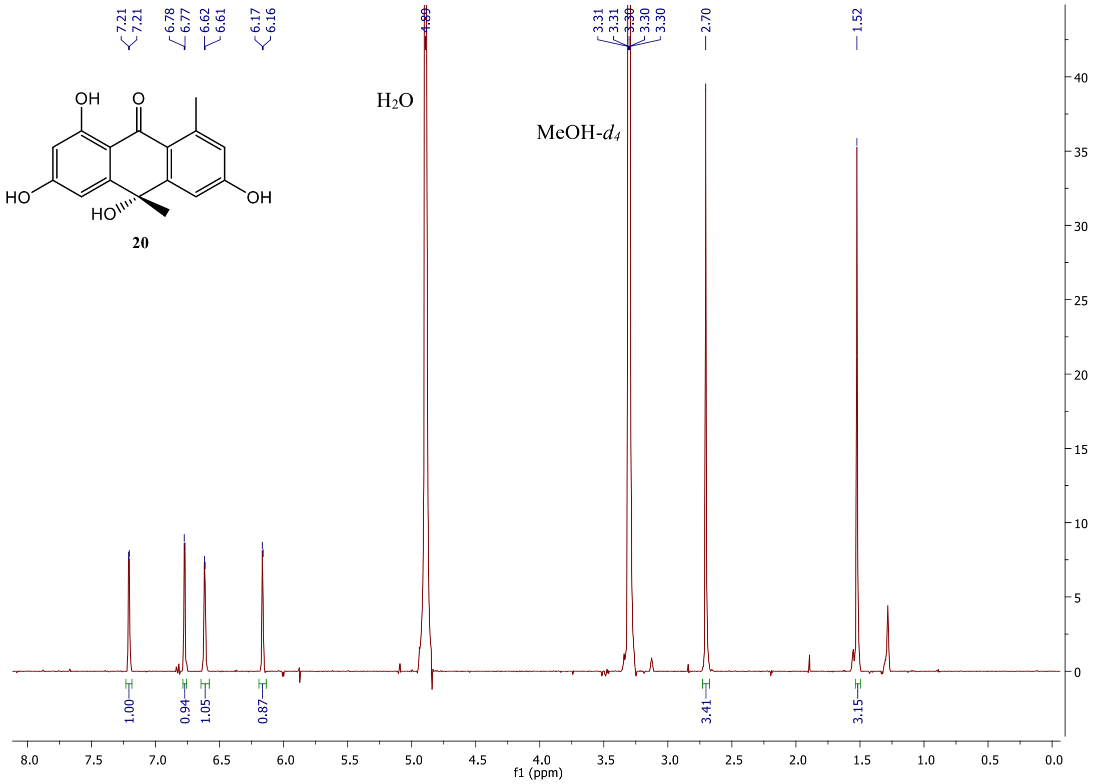

Figure S47: ^{13}C NMR (100 MHz, MeOH-d_4) spectrum of compound **20**

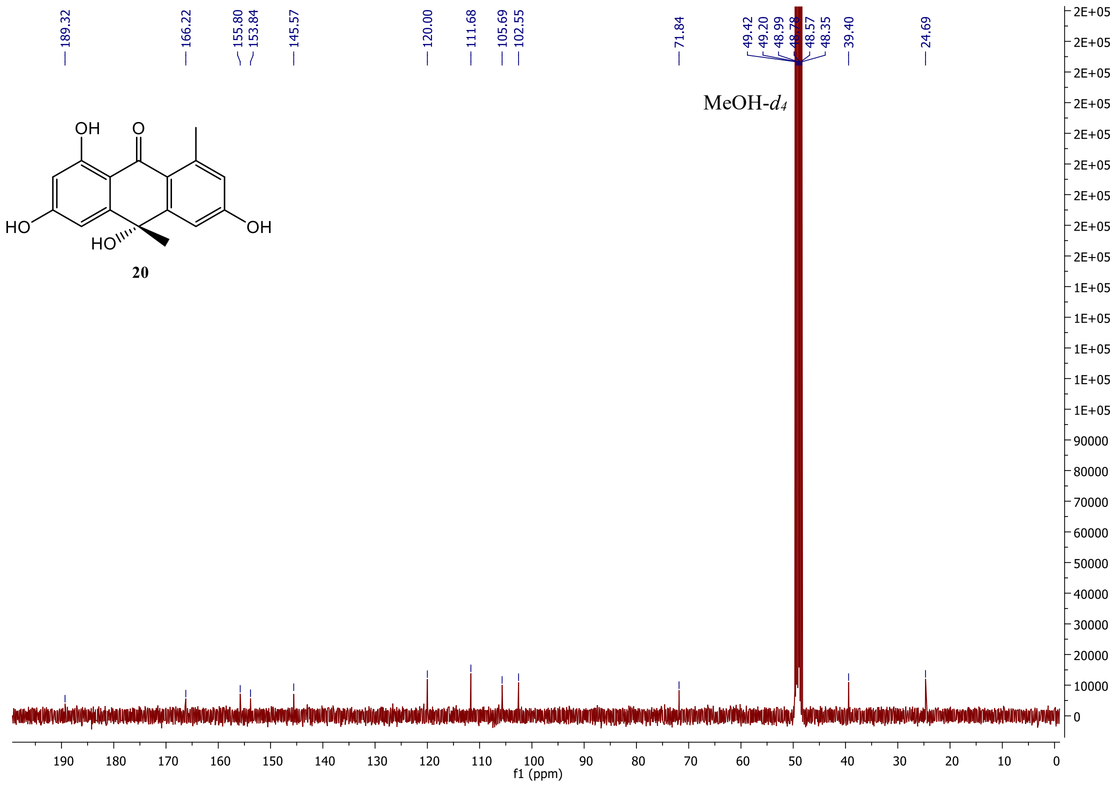

Figure S48: ^1H-^1H COSY (400 MHz, MeOH-d_4) spectrum of compound **20**

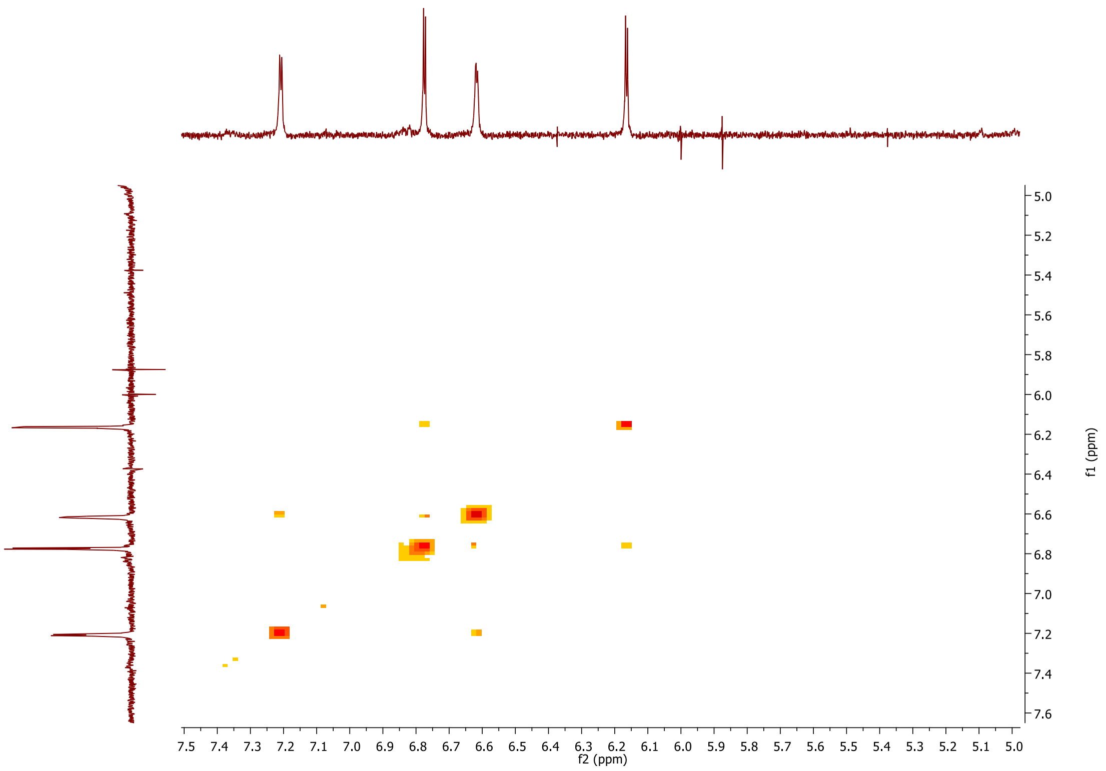

Figure S49a: gHSQC (400 MHz and 100MHz, MeOH-d_4) spectrum of compound **20**

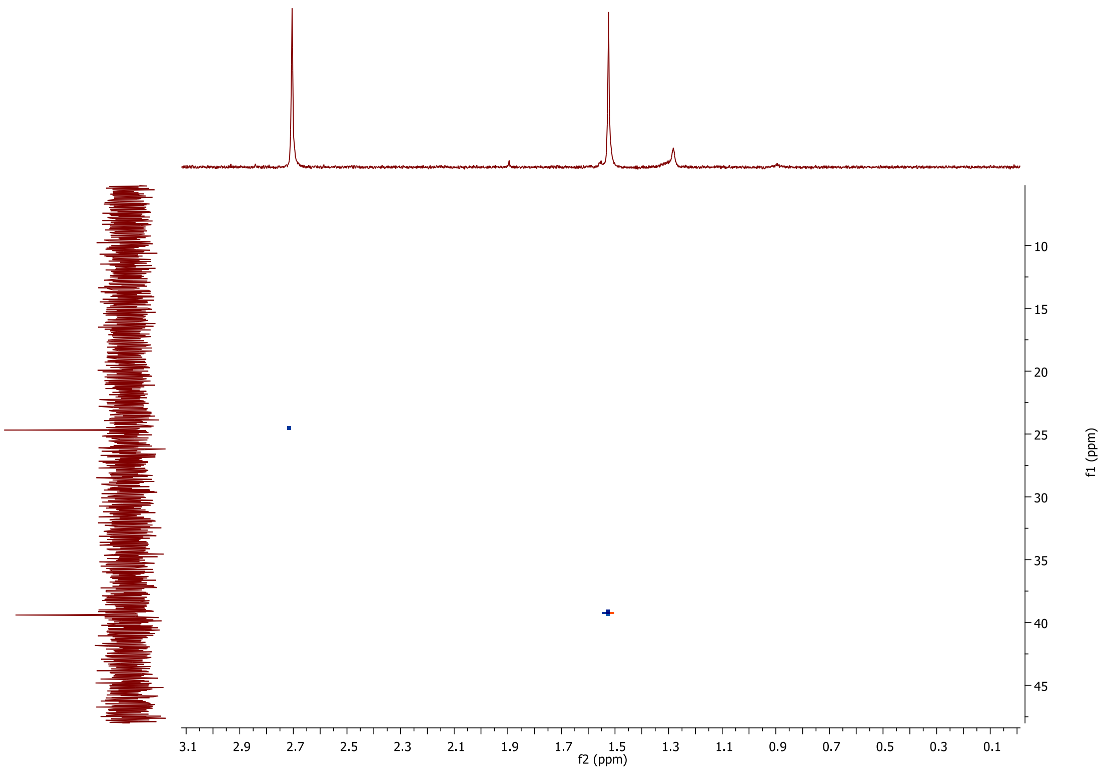

Figure S49b: gHSQC (400 MHz and 100MHz, MeOH-d_4) spectrum of compound **20**

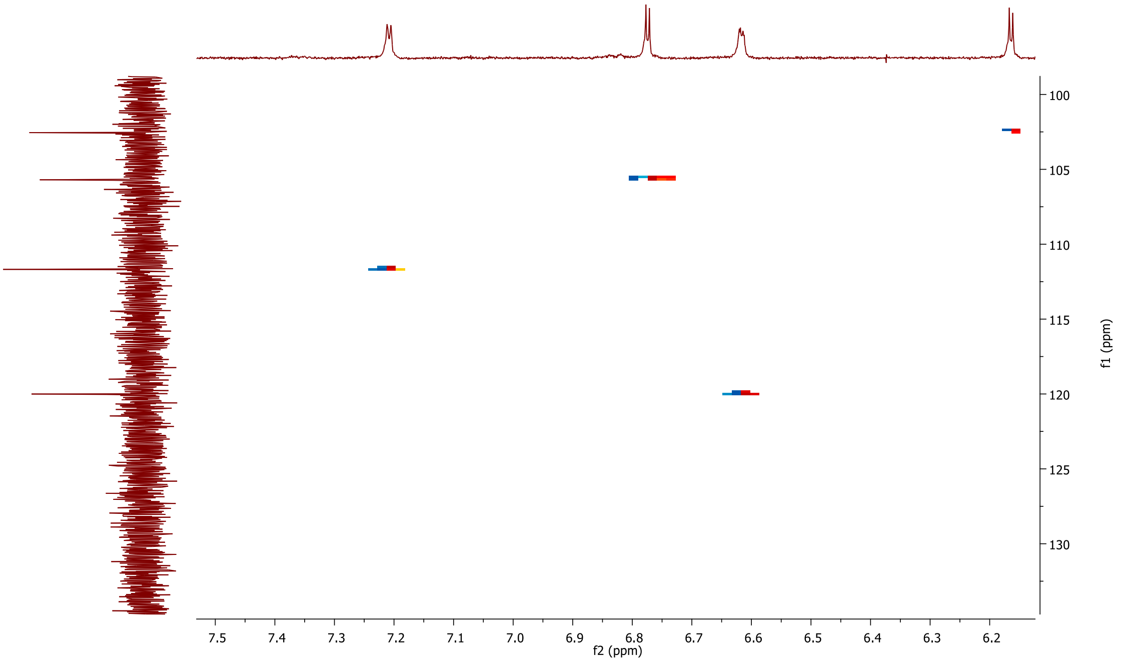

Figure S50a: gHMBC (400 MHz and 100MHz, MeOH-d_4) spectrum of compound **20**

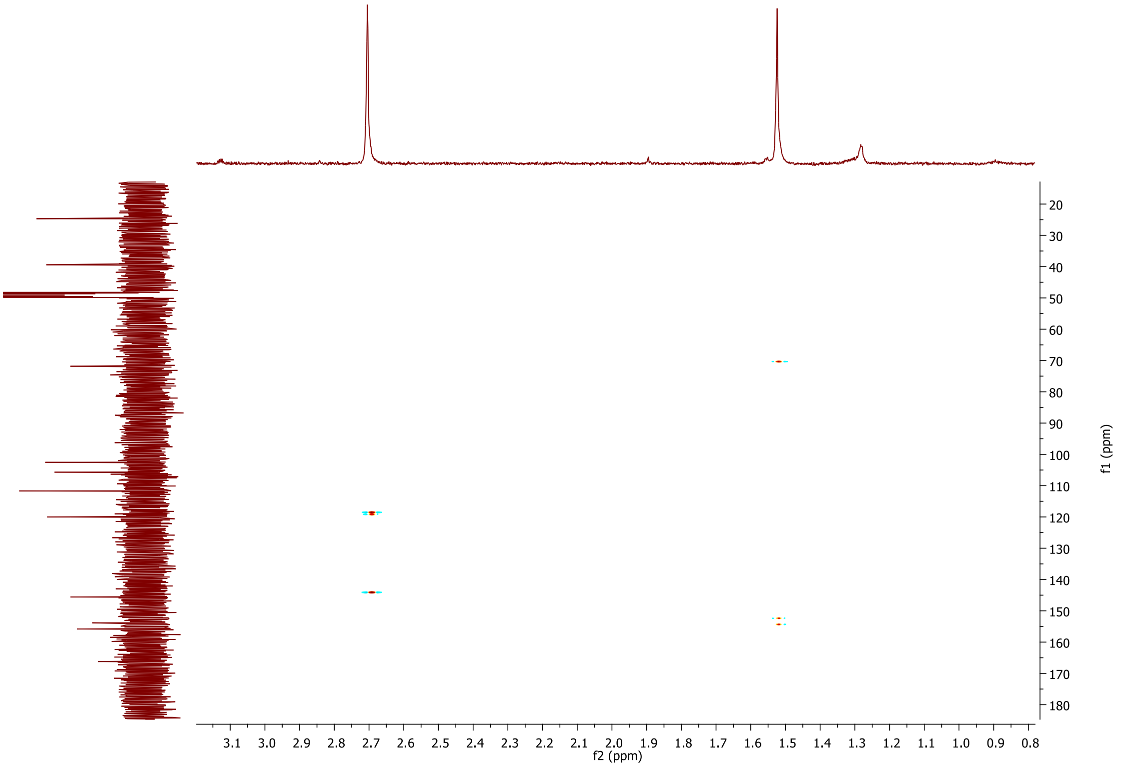

Figure S50b: gHMBC (400 MHz and 100MHz, MeOH-d_4) spectrum of compound **20**

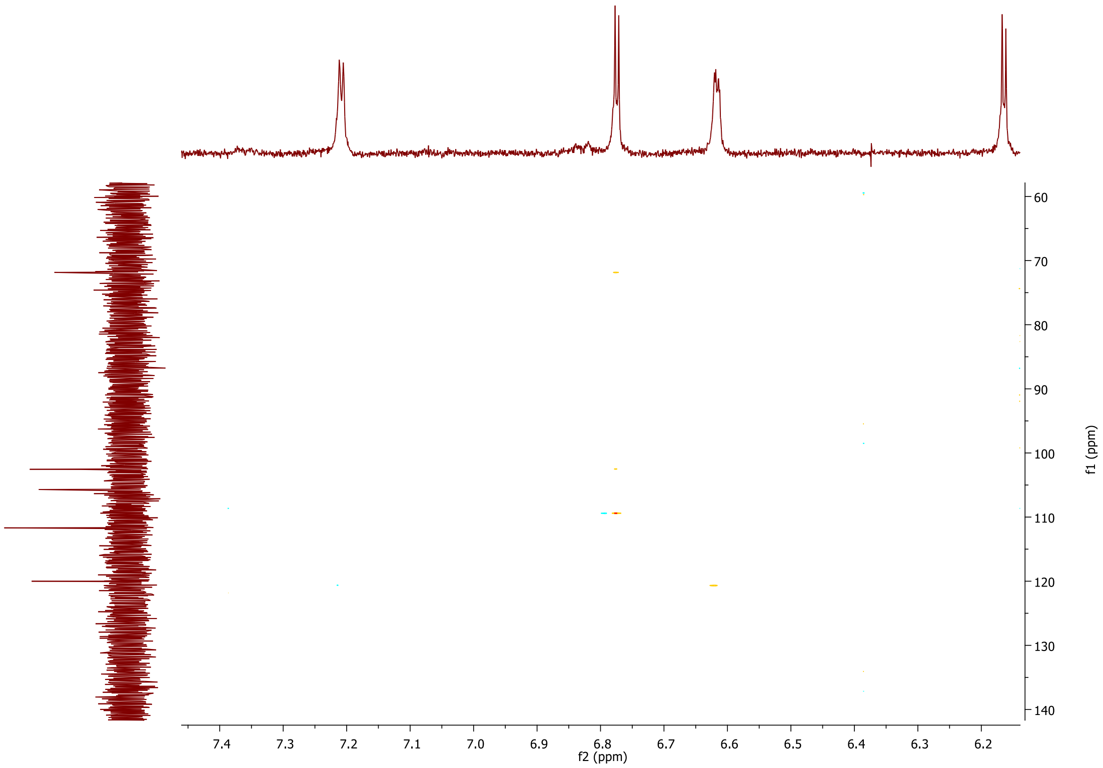

Figure S51: HRESIMS spectrum of compound **21**

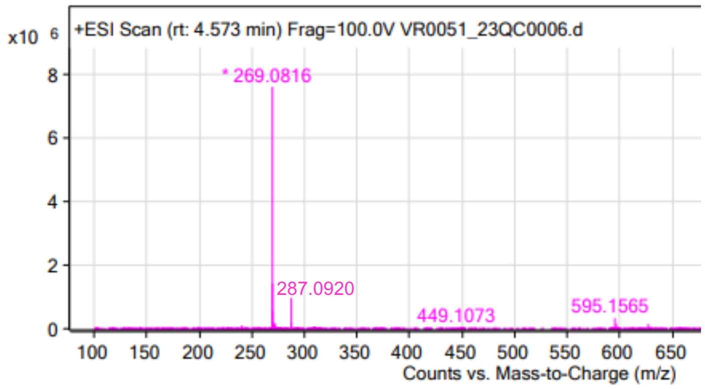

Figure S52: UV absorption spectrum of compound **21**

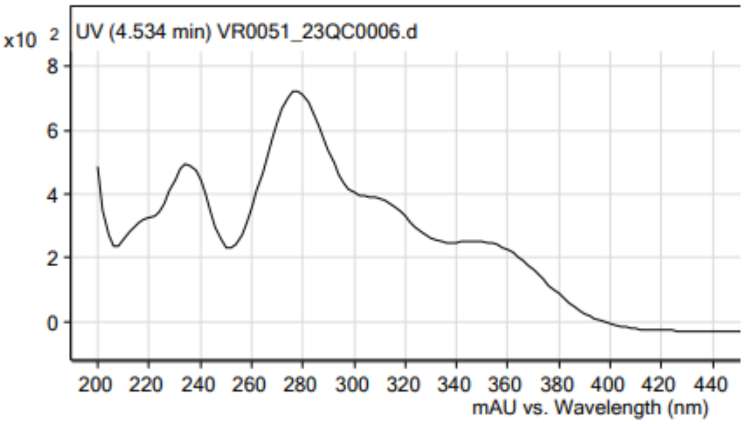

Figure S53: IR spectrum of compound **21**

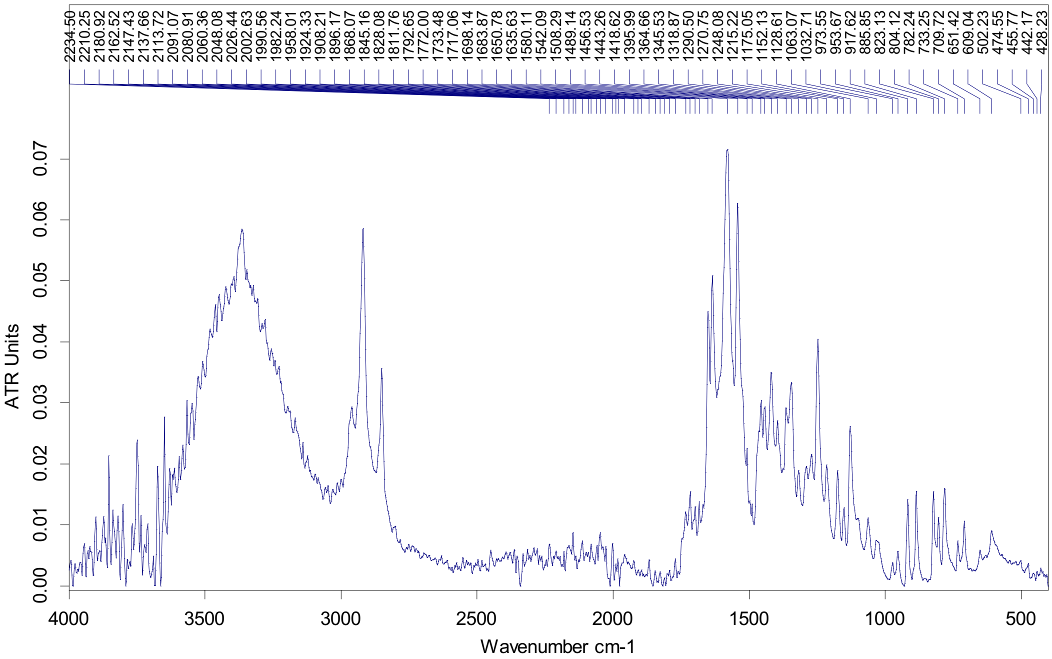

Figure S54: ^{1}H NMR (400 MHz, $CDCl_3$) spectrum of compound **21**

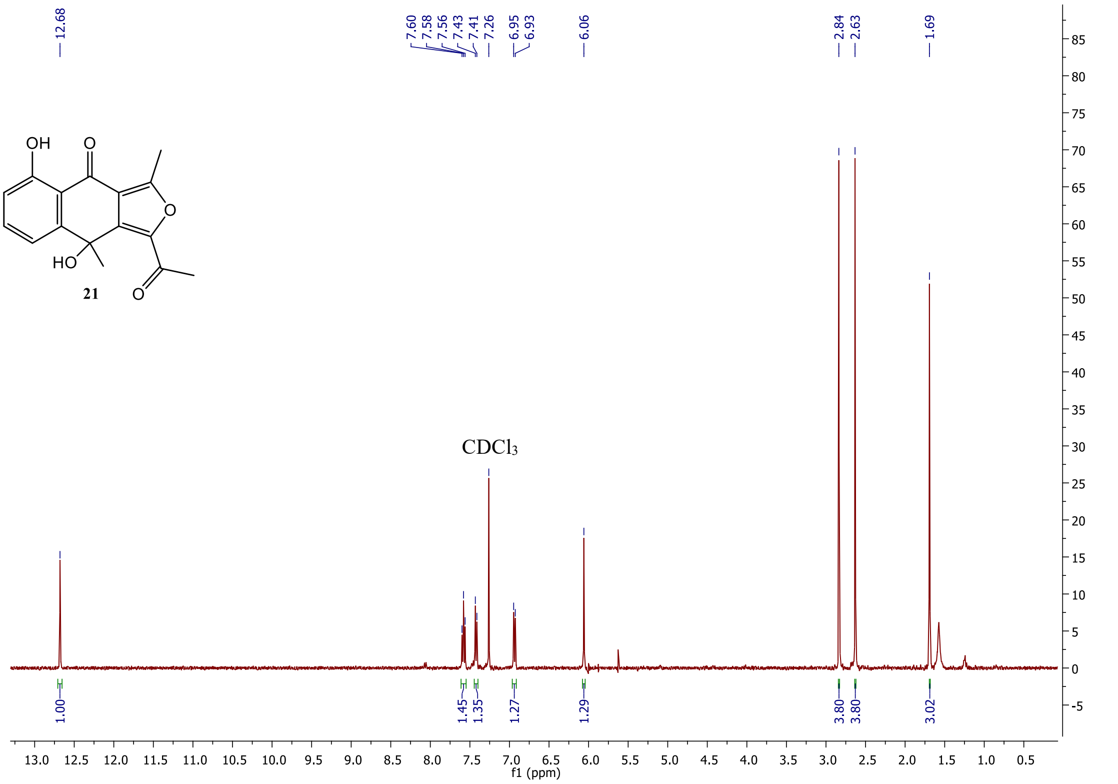

Figure S55: ^{13}C NMR (100 MHz, $CDCl_3$) spectrum of compound **21**

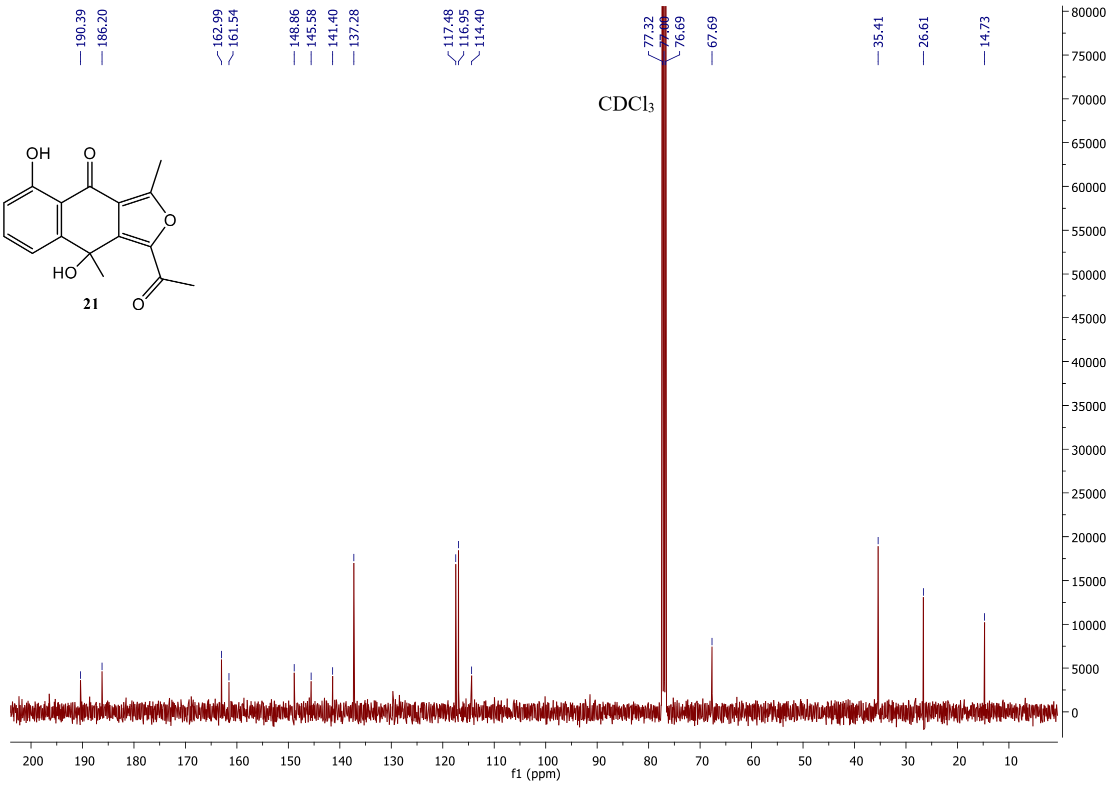

Figure S56: ^1H-^1H COSY (400 MHz, $CDCl_3$) spectrum of compound **21**

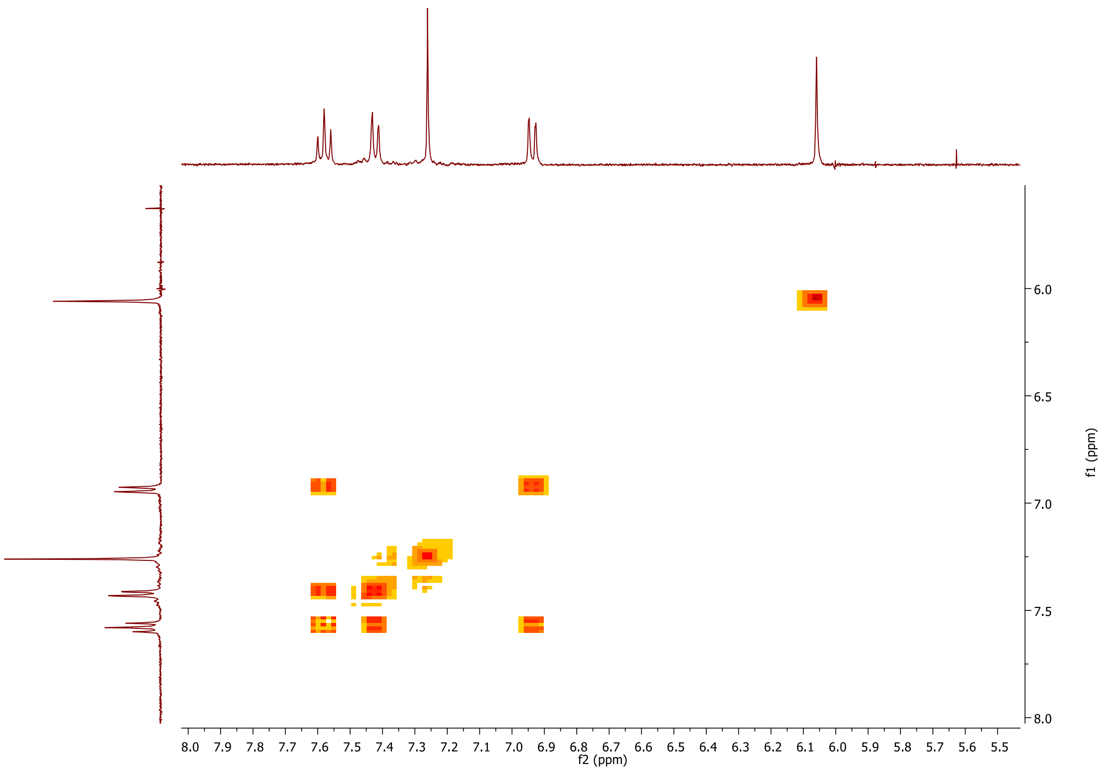

Figure S57a: gHSQC (400 MHz and 100MHz, $CDCl_3$) spectrum of compound **21**

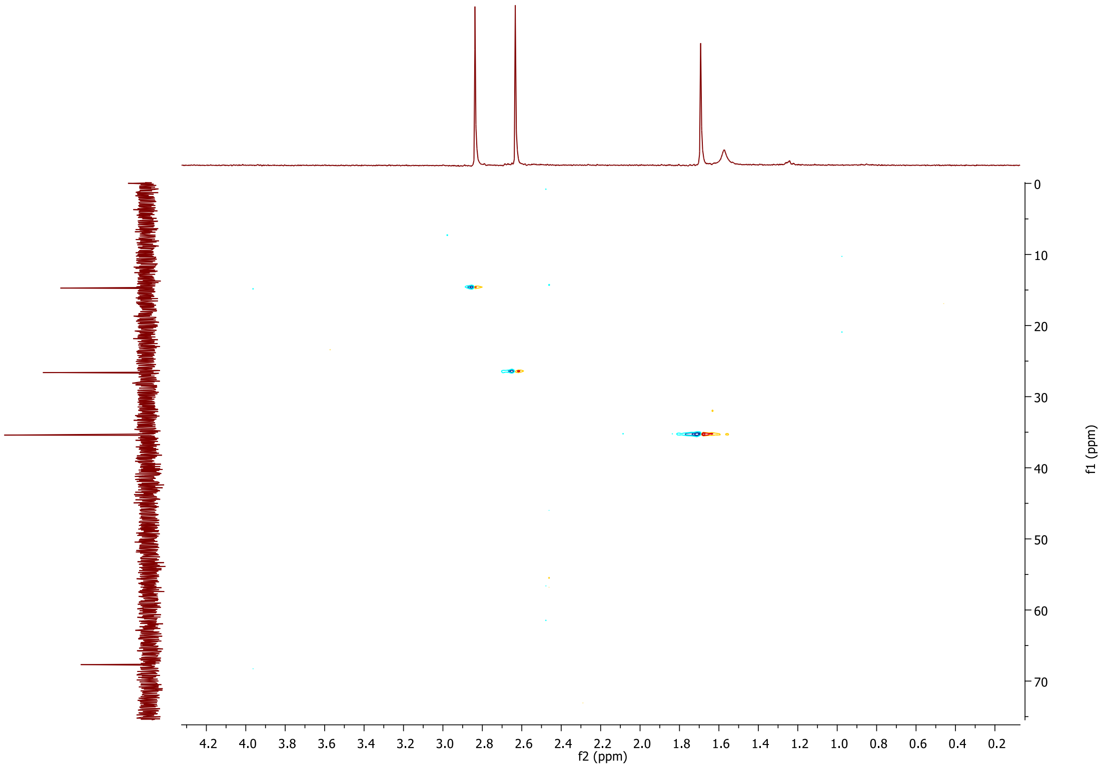

Figure S57b: gHSQC (400 MHz and 100MHz, $CDCl_3$) spectrum of compound **21**

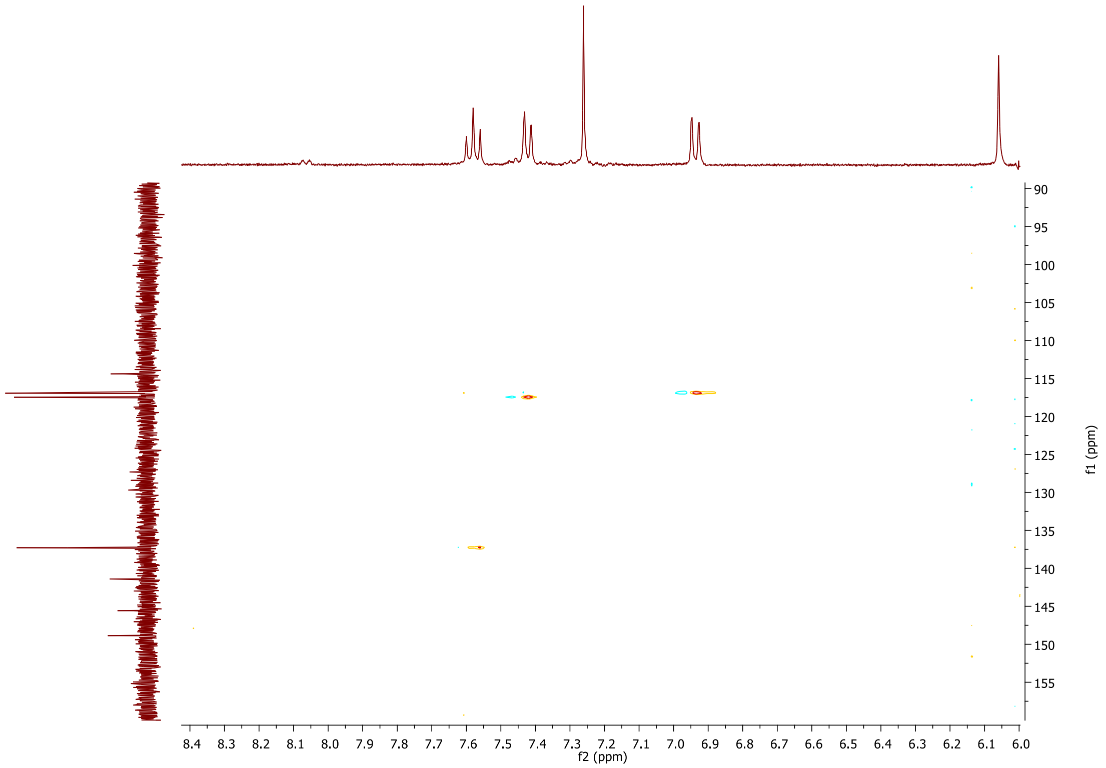

Figure S58a: gHMBC (400 MHz and 100MHz, $CDCl_3$) spectrum of compound **21**

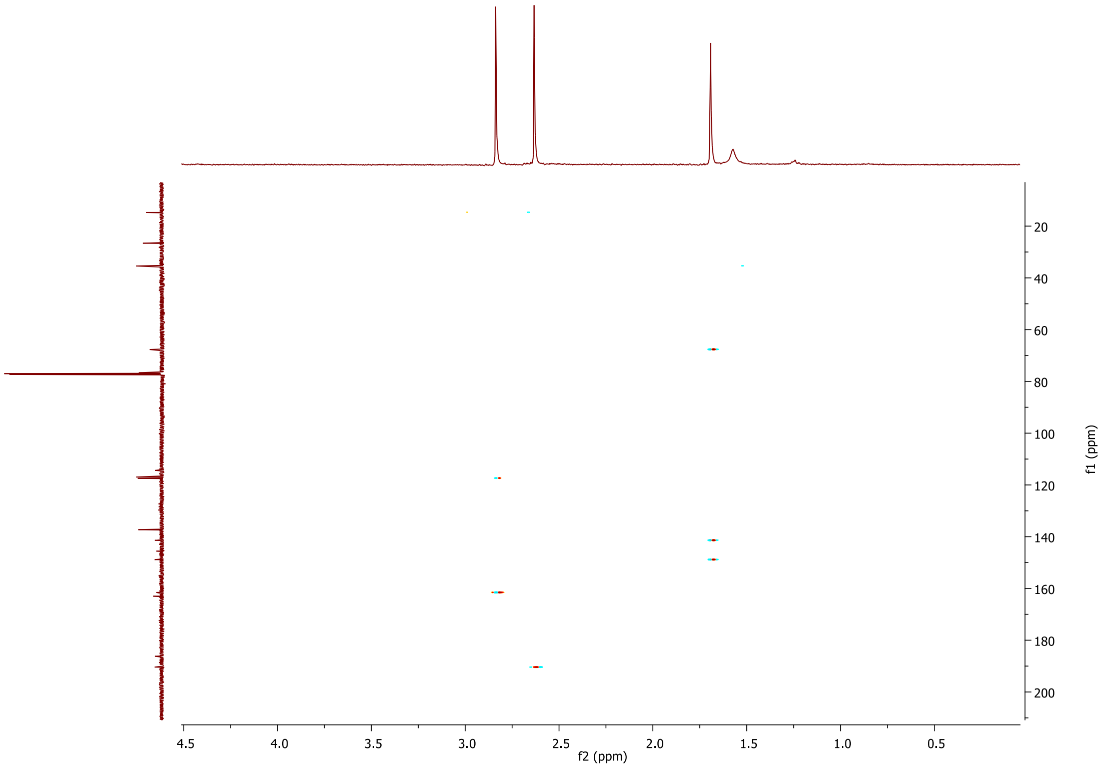

Figure S58b: gHMBC (400 MHz and 100MHz, $CDCl_3$) spectrum of compound **21**

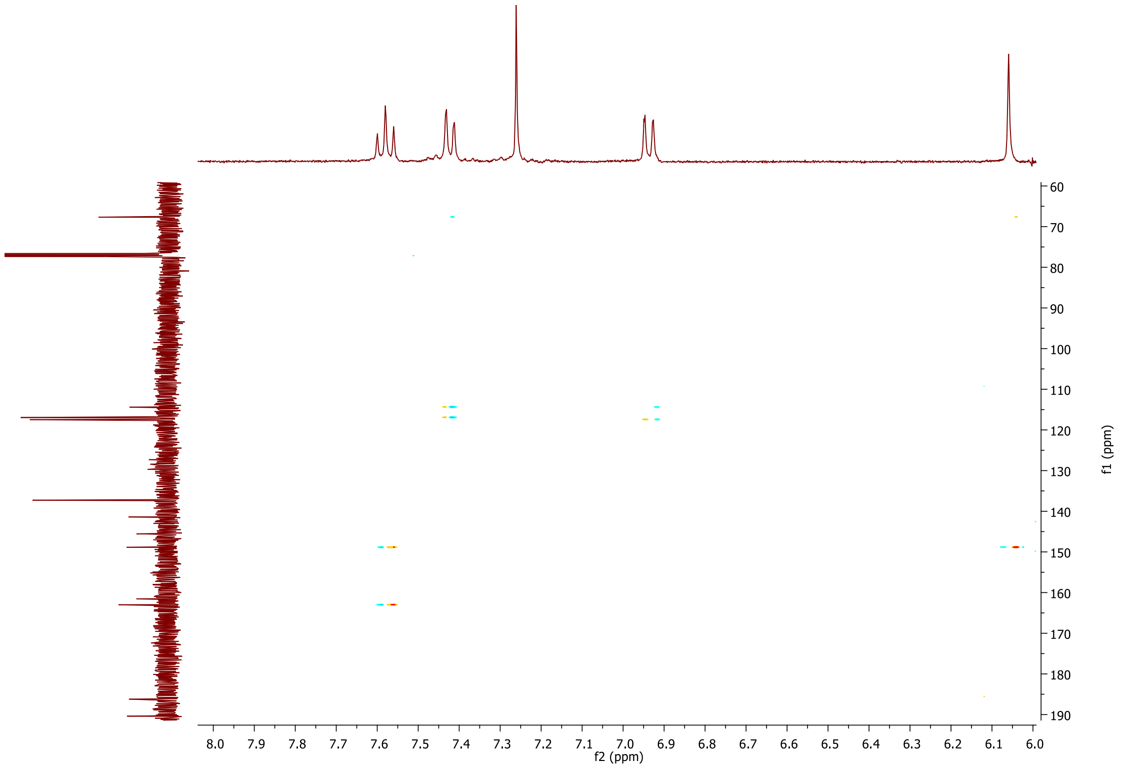

Figure S58c: gHMBC (400 MHz and 100MHz, $CDCl_3$) spectrum of compound **21**

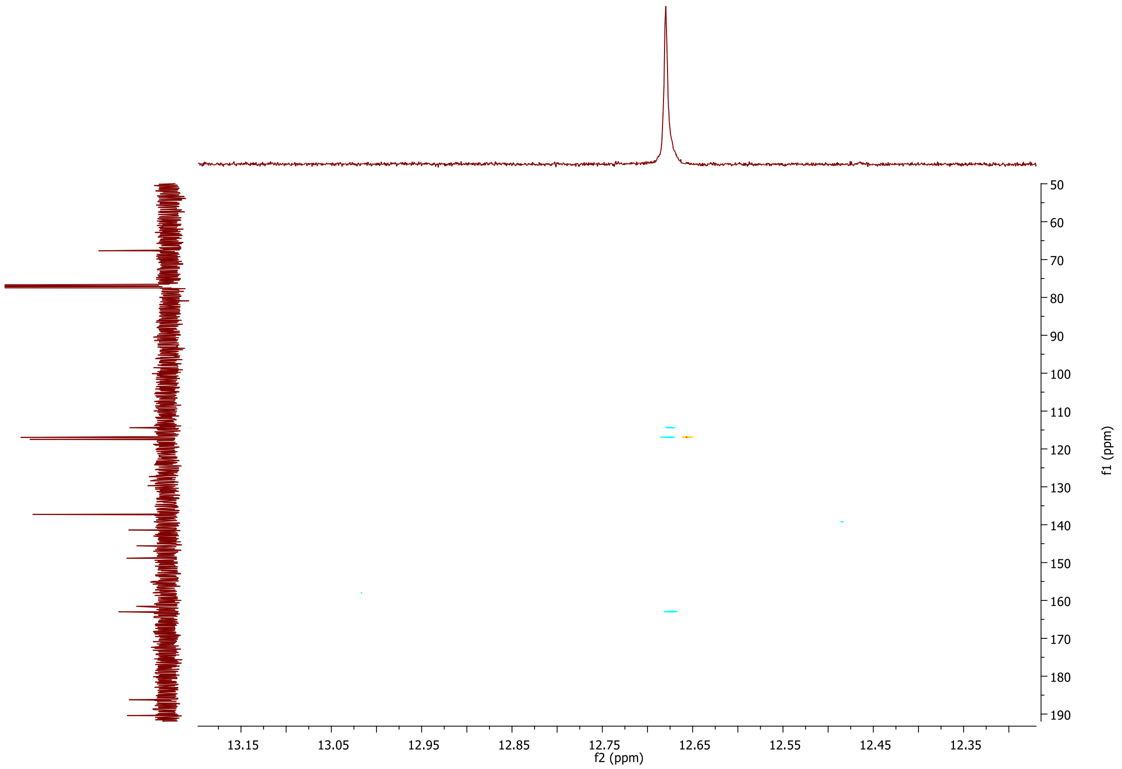

Figure S59: HRESIMS spectrum of compound **22**

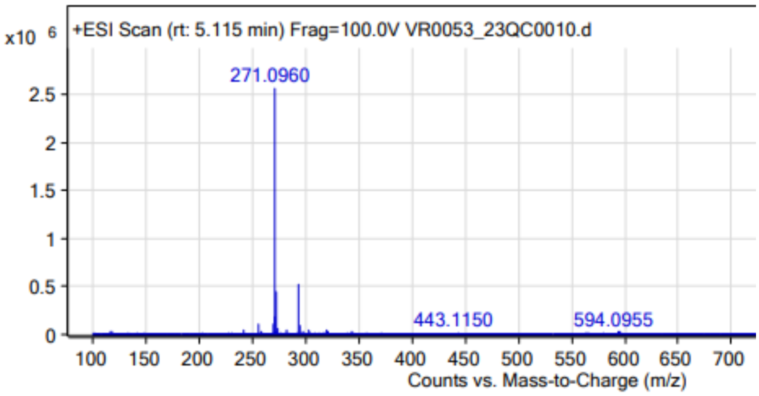

Figure S60: UV absorption spectrum of compound **22**

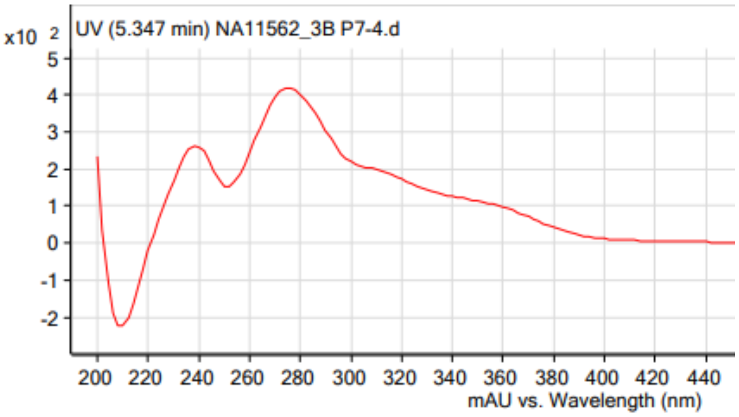

Figure S61: IR spectrum of compound **22**

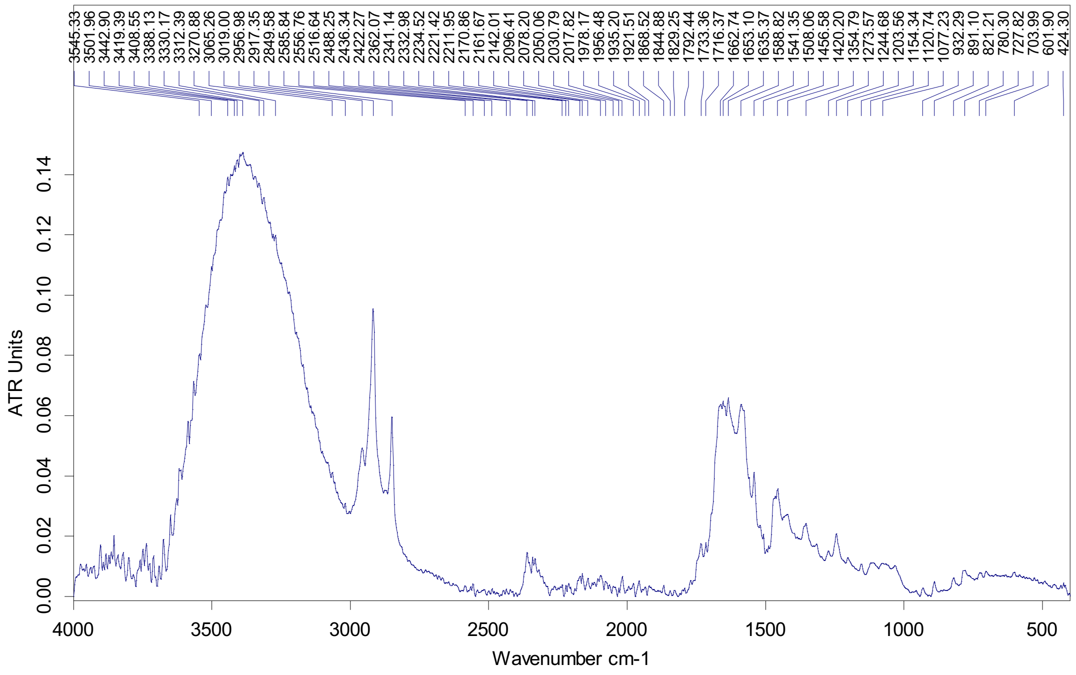

Figure S62: ^{1}H NMR (400 MHz, $CDCl_3$) spectrum of compound **22**

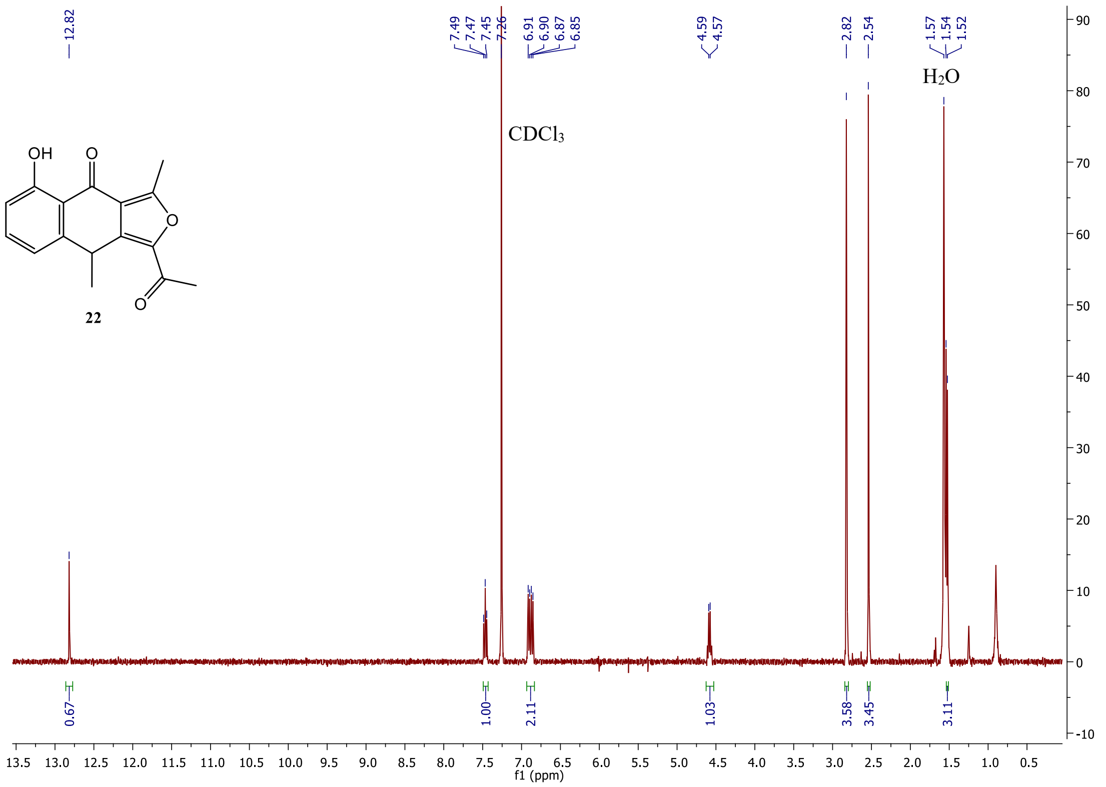

Figure S63: ^{1}H-^{1}H COSY (400 MHz, $CDCl_3$) spectrum of compound **22**

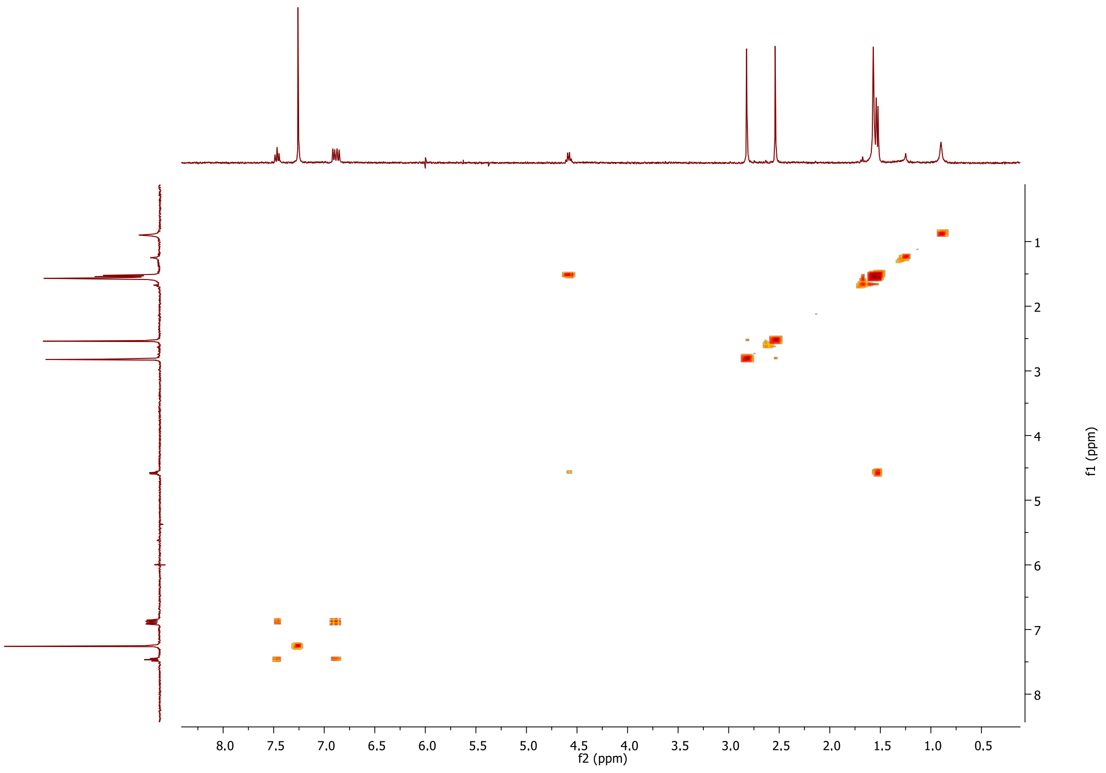

Figure S64a: gHMBC (400 MHz and 100MHz, $CDCl_3$) spectrum of compound **22**

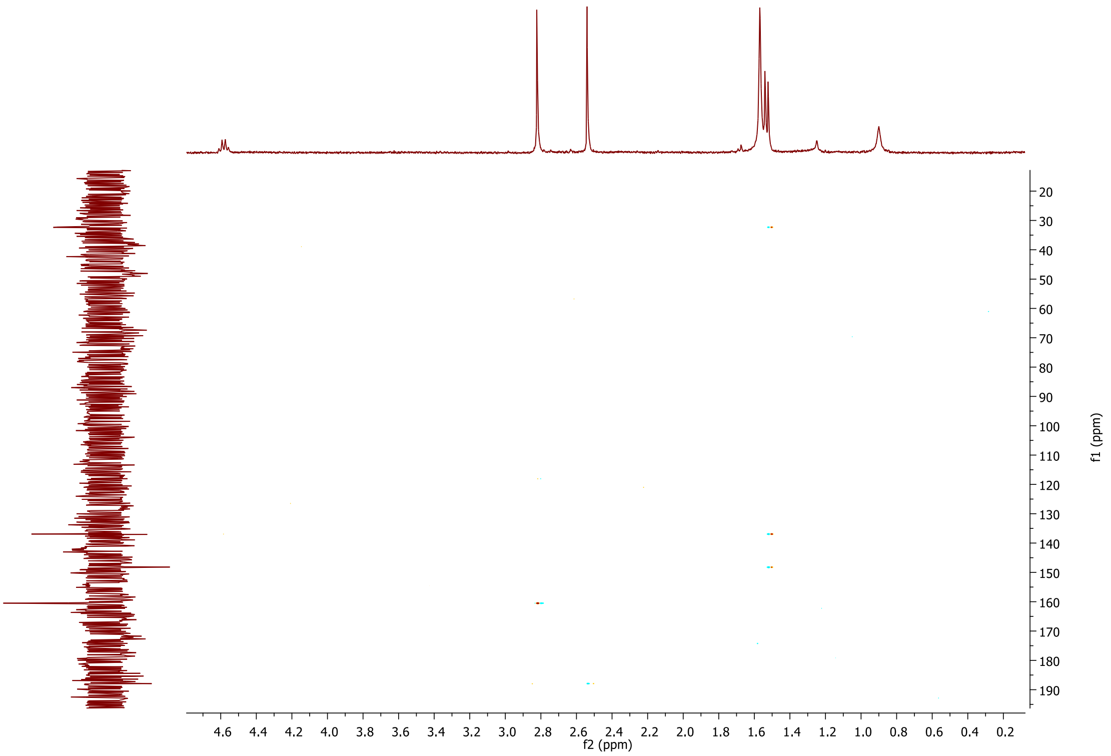

Figure S64b: gHMBC (400 MHz and 100MHz, $CDCl_3$) spectrum of compound **22**

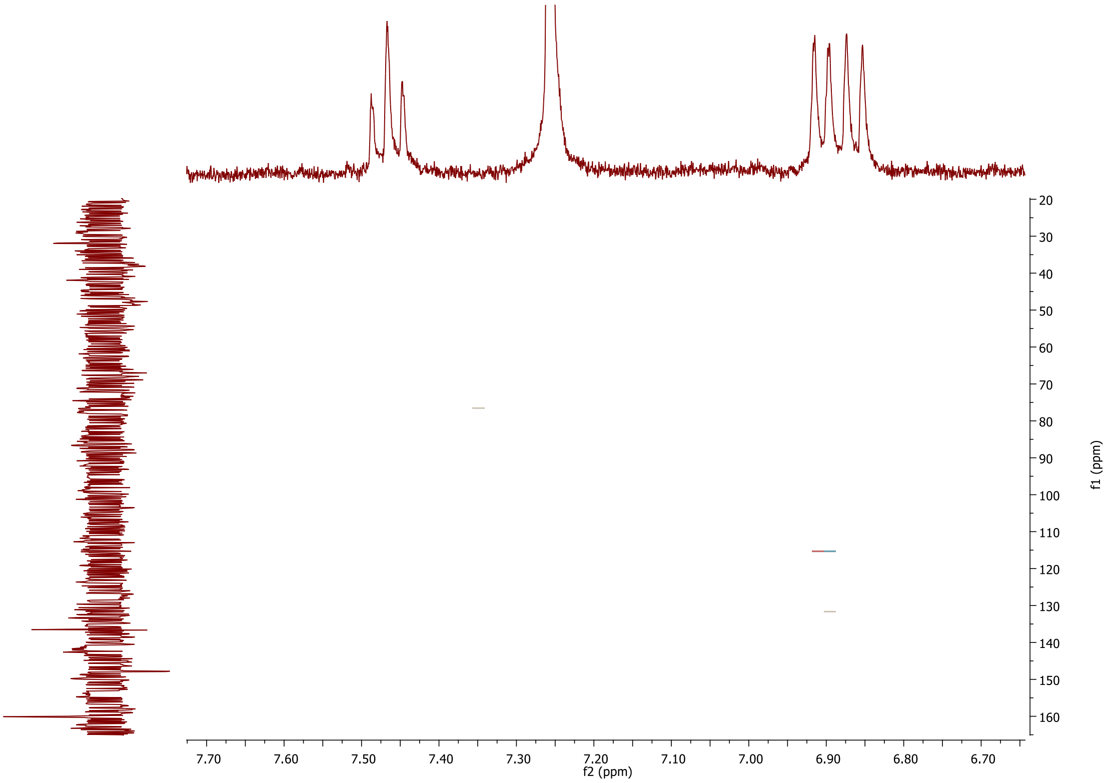

Figure S65: HRESIMS spectrum of compound **23**

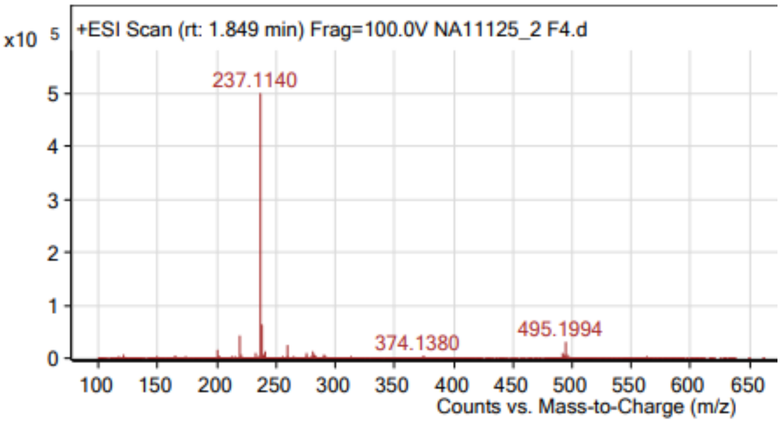

Figure S66: UV absorption spectrum of compound **23**

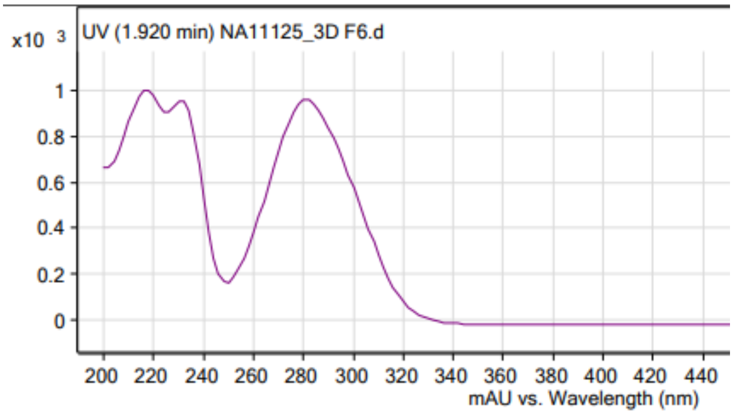

Figure S67: IR spectrum of compound **23**

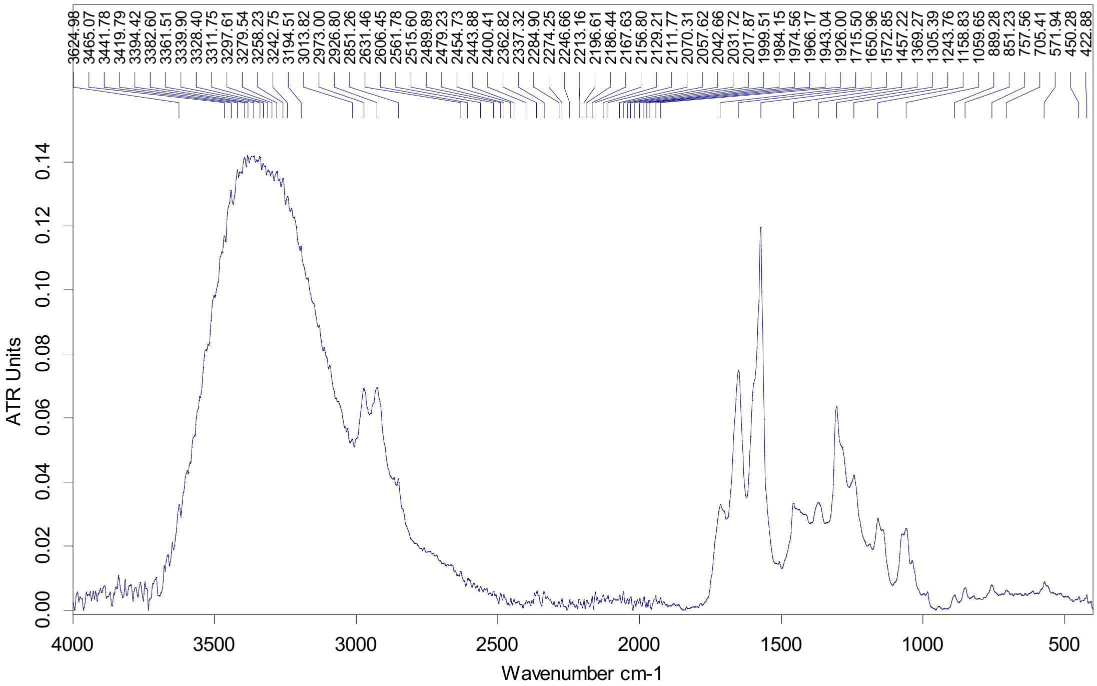

Figure S68: ^{1}H NMR (400 MHz, $CDCl_3$ + MeOH-d_4) spectrum of compound **23**

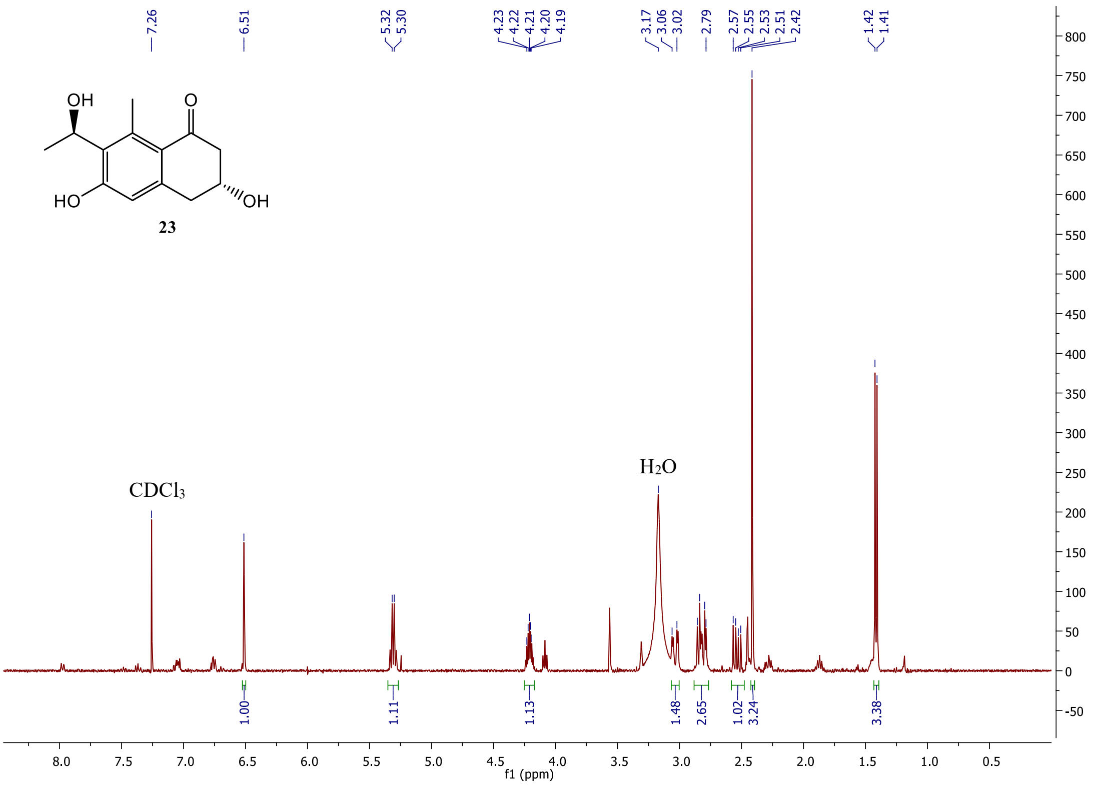

Figure S69: ^{13}C NMR (100 MHz, $CDCl_3$ + MeOH-d_4) spectrum of compound **23**

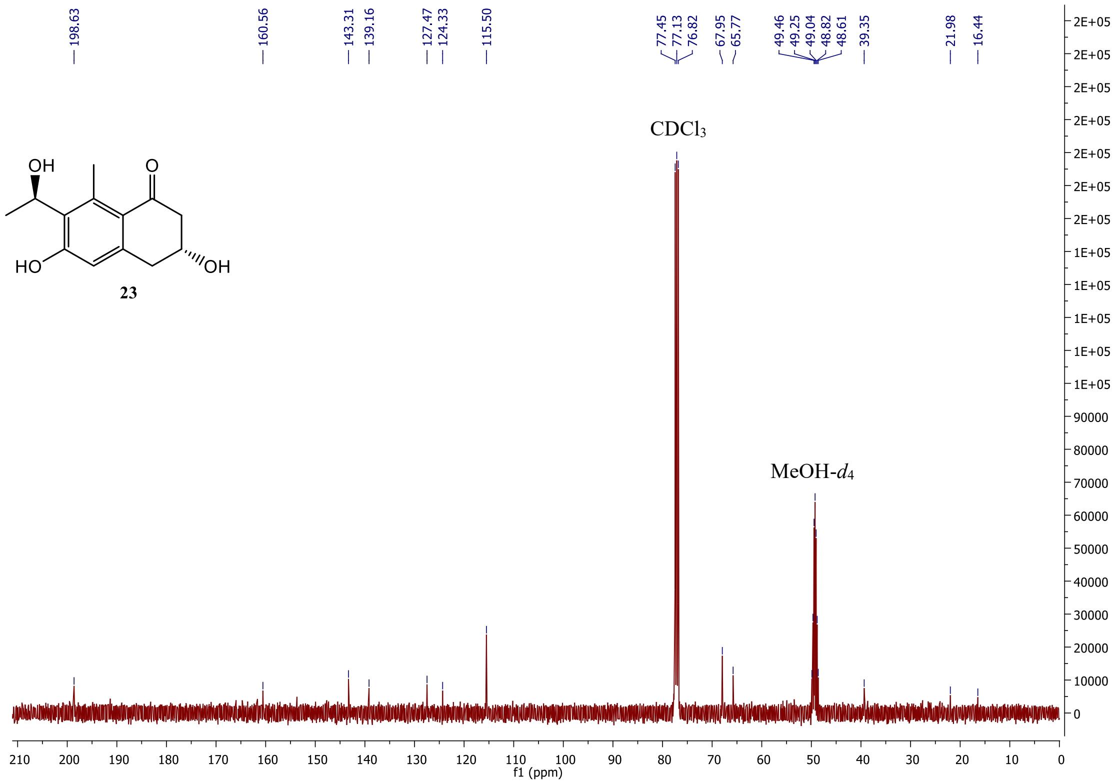

Figure S70: ^1H-^1H COSY (400 MHz, $CDCl_3$ + MeOH-d_4) spectrum of compound **23**

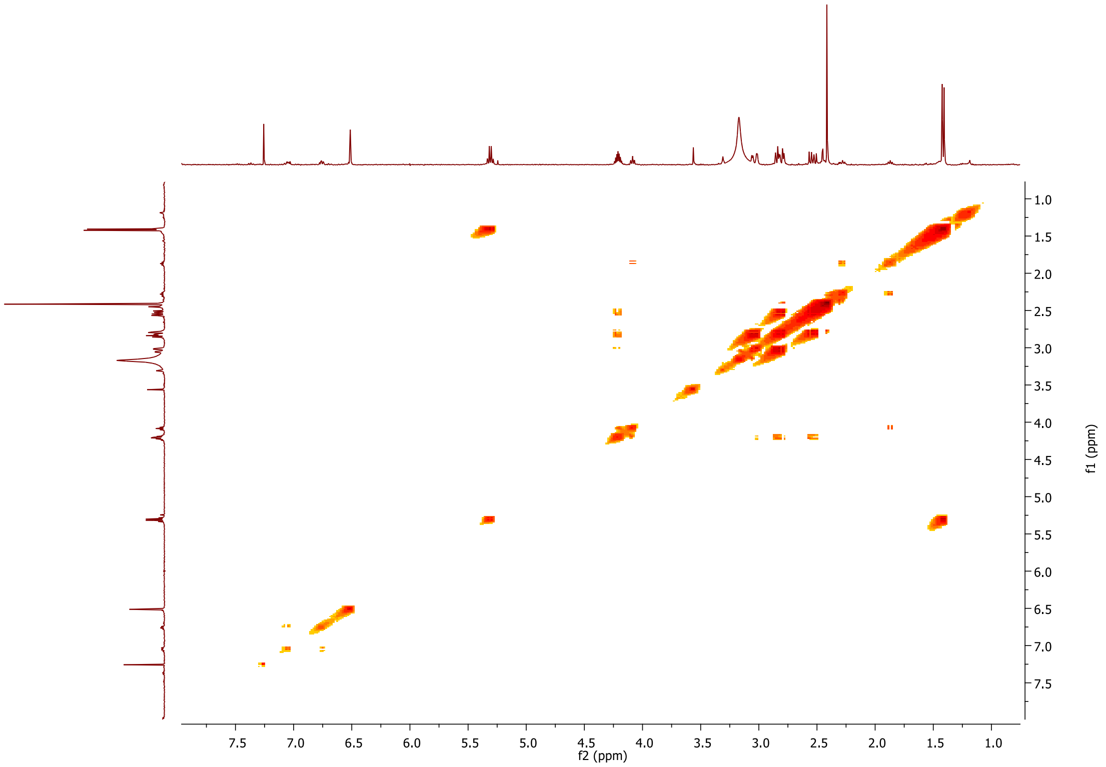

Figure S71: gHSQC (400 MHz and 100MHz, $CDCl_3$ + MeOH-d_4) spectrum of compound **23**

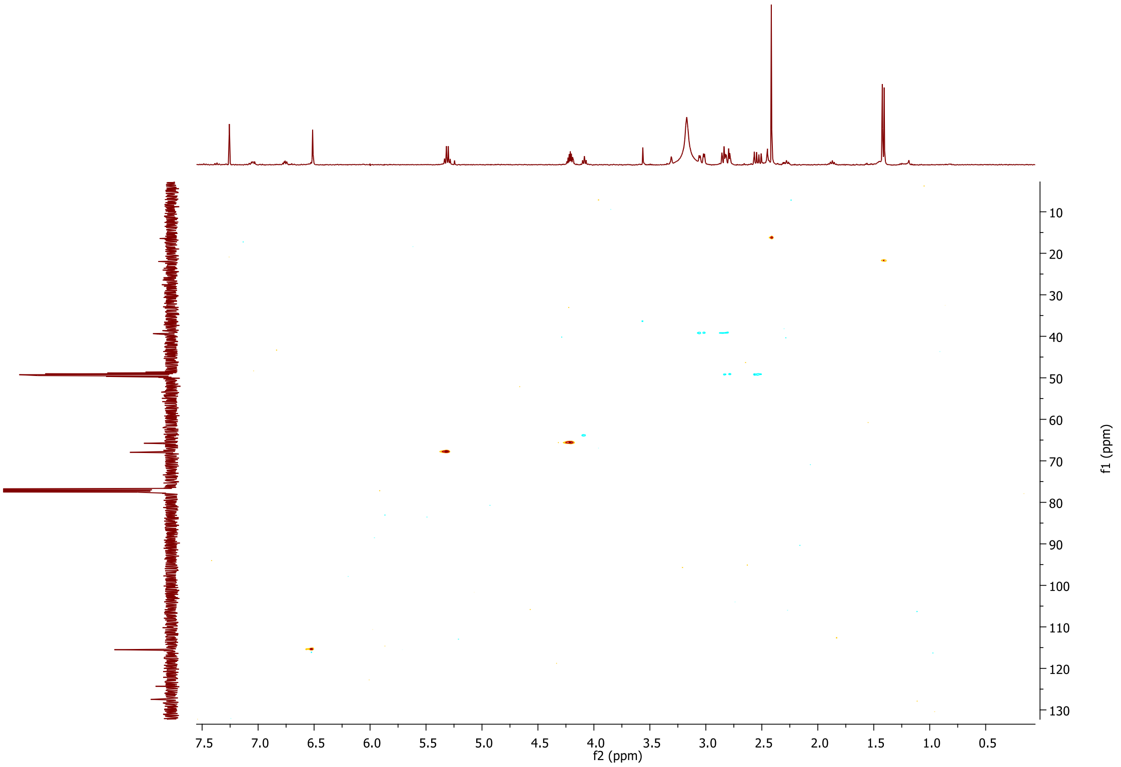

Figure S72: gHMBC (400 MHz and 100MHz, $CDCl_3$ + MeOH-d_4) spectrum of compound **23**

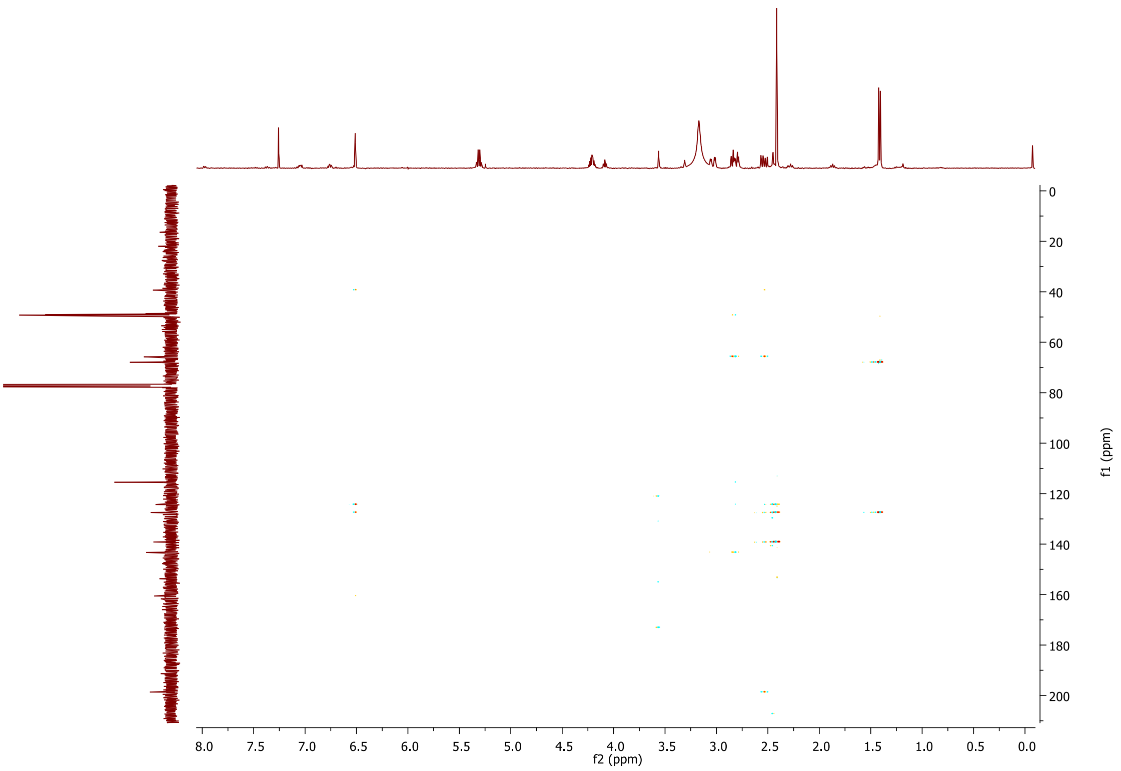

3. Computational section

3.1. Computational details

All calculations were done following the general protocols previously described for *J*-DP4.[1] Conformational searches were done using the Mixed Torsional/Low Mode Sampling protocol in gas phase using the MMFF (as implemented in Macromodel) [2] force field and reoptimizing the conformers with AMBER and MM3 (as recommended in mix-*J*-DP4).[3] All conformers within 12 kJ/mol of the lowest energy conformer were subjected to NMR calculations. DFT calculations were performed using Gaussian 16.[4] Structure optimizations were done at the B3LYP/6-31G* level of theory. Magnetic shielding constants (σ) were calculated by means of the gauge including atomic orbitals (GIAO) method,[5] currently the most widely used to solve the gauge origin problem, at B3LYP/6-31G** level as used in *J*-DP4. Unscaled chemical shifts (δ_u) were calculated using TMS as reference standard according to the following expression $\delta_u = \sigma_0 - \sigma_x$, where σx is the Boltzmann averaged shielding tensor (over all significantly populated conformations) and σ_0 is the shielding tensor of TMS computed at the same level of theory used to calculate σ_x. Boltzmann averaging was done according to equation S1:

$$\sigma^x = \frac{\sum_i \sigma_i^x e^{(-E_i/RT)}}{\sum_i e^{(-E_i/RT)}}$$

(Equation S1)

where σ^x_i is the shielding constant for nucleus x in conformer i, R is the molar gas constant (8.3145 J /(K mol)), T is the temperature used for the calculation (298 K), and E_i is the relative energy of conformer i (to the lowest energy conformer) obtained from a single-point NMR calculation at the corresponding level of theory. The scaled chemical shifts (δs) were computed as δs = (δu − b)/m, where m and b are the slope and intercept, respectively, resulting from a linear regression calculation on a plot of δu against δexp.

(1) Grimblat, N.; Gavín, J. A.; Hernández Daranas, A.; Sarotti, A. M. Combining the Power of J Coupling and DP4 Analysis on Stereochemical Assignments: The J-DP4 Methods. Org. Lett. 2019, 21 (11), 4003–4007.

(2) Cuadrado C, Daranas AH, Sarotti AM. May the Force (Field) Be with You: On the Importance of Conformational Searches in the Prediction of NMR Chemical Shifts. Mar Drugs 2022; 20(11). e-pub ahead of print 2022/11/11; doi: 10.3390/md20110699

(3) MacroModel Schrodinger release 2018-3; Schrodinger LLC: New York, 2018.

(4) Gaussian 16, Revision C.01, M. J. Frisch, G. W. Trucks, H. B. Schlegel, G. E. Scuseria, M. A. Robb, J. R. Cheeseman, G. Scalmani, V. Barone, G. A. Petersson, H. Nakatsuji, X. Li, M. Caricato, A. V. Marenich, J. Bloino, B. G. Janesko, R. Gomperts, B. Mennucci, H. P. Hratchian, J. V. Ortiz, A. F. Izmaylov, J. L. Sonnenberg, D. Williams-Young, F. Ding, F. Lipparini, F. Egidi, J. Goings, B. Peng, A. Petrone, T. Henderson, D. Ranasinghe, V. G. Zakrzewski, J. Gao, N. Rega, G. Zheng, W. Liang, M. Hada, M. Ehara, K. Toyota, R. Fukuda, J. Hasegawa, M. Ishida, T. Nakajima, Y. Honda, O. Kitao, H. Nakai, T. Vreven, K. Throssell, J. A. Montgomery, Jr., J. E. Peralta, F. Ogliaro, M. J. Bearpark, J. J. Heyd, E. N. Brothers, K. N. Kudin, V. N. Staroverov, T. A. Keith, R. Kobayashi, J. Normand, K. Raghavachari, A. P. Rendell, J. C. Burant, S. S. Iyengar, J. Tomasi, M. Cossi, J. M. Millam, M. Klene, C. Adamo, R. Cammi, J. W. Ochterski, R. L. Martin, K. Morokuma, O. Farkas, J. B. Foresman, and D. J. Fox, Gaussian, Inc., Wallingford CT, 2016.

(5) (a) Ditchfield, R. J. Chem. Phys. 1972, 56, 5688-5691. b) Ditchfield, R. Mol. Phys.1974, 27, 789-807. c) Rohlfing, C. M.; Allen, L. C.; Ditchfield, R. Chem. Phys. 1984, 87, 9-15. d) Wolinski, K.; Hinton, J. F.; Pulay, P. J. Am. Chem. Soc. 1990, 112, 8251-8260.

3.2. Isomer studied for compound 23.

Table S1. Configuration of isomers studied for compound **23**.

Isomer	Configuration
**Isomer 1**	3R*, 9S*
**Isomer 2**	3R*, 9R*

3.3. Experimental chemical shifts and isotropic magnetic shielding constants of studied isomers.

Table S2. Boltzmann averaged GIAO isotropic magnetic shielding constants (σ) of isomers 1-2 calculated at the B3LYP/6-31G** level of theory for compound **23**.

Isotropic shielding constants (AMBER)			
Nuclei	Experimental	Isomer 1	Isomer 2
C 1	198.7	2.95	2.03
C 2	49.4	142.84	141.15
C 3	65.8	124.62	125.32
C 4	39.4	151.27	152.97
C 4a	143.3	50.48	51.73
C 5	115.5	81.40	81.35
C 6	160.6	36.49	36.55
C 7	127.5	67.59	67.56
C 8	139.2	51.65	52.86
C 8a	124.3	68.54	68.27
C 9	68.0	122.24	122.39
C 10	22.0	169.77	169.84
C 11	16.4	172.51	172.86
H 2a	2.53	29.29	29.29
H 2b	2.81	29.62	29.59
H 3	4.21	27.73	27.73
H 4a	3.03	29.03	28.90
H 4b	2.83	29.13	29.07
H 5	6.51	25.31	25.29
H 9	5.31	26.55	26.56
H 10a-c	1.42	30.37	30.36
H 11a-c	2.42	29.64	29.71
J 2a,3	8	3.71	7.88
J 2b,3	4	8.11	3.61
J 4a,3	8	3.38	7.45
J 4b,3	4	7.67	3.92

		Isotropic shielding constants (MM3)	
Nuclei	Experimental	Isomer 1	Isomer 2
C 1	198.7	6.19	6.36
C 2	49.4	141.27	139.86
C 3	65.8	121.81	122.63
C 4	39.4	147.97	147.92
C 4a	143.3	52.37	52.75
C 5	115.5	79.7	79.31
C 6	160.6	39.26	39.26
C 7	127.5	70.24	70.09
C 8	139.2	53.92	53.89
C 8a	124.3	70.33	70.68
C 9	68.0	120.9	120.9
C 10	22.0	166.56	166.6
C 11	16.4	169.37	169.32
H 2a	2.53	28.65	28.8
H 2b	2.81	29.03	28.87
H 3	4.21	27.33	27.36
H 4a	3.03	28.44	28.34
H 4b	2.83	28.51	28.51
H 5	6.51	24.98	24.95
H 9	5.31	26.07	26.07
H 10a-c	1.42	29.81	29.8
H 11a-c	2.42	29.05	29.05
J 2a,3	8	3.9	9.13
J 2b,3	4	8.12	3.7
J 4a,3	8	3.41	8.39
J 4b,3	4	7.79	3.9

		Isotropic shielding constants (MMFF)	
Nuclei	Experimental	Isomer 1	Isomer 2
C 1	198.7	0.76	0.65
C 2	49.4	144.36	142.86
C 3	65.8	124.51	125.1
C 4	39.4	151.44	152.93
C 4a	143.3	51.91	52.77
C 5	115.5	79.14	78.96
C 6	160.6	37.33	37.35
C 7	127.5	70.24	70.2
C 8	139.2	51.47	52.17
C 8a	124.3	68.5	68.47
C 9	68.0	120.19	120.23
C 10	22.0	169.36	169.35
C 11	16.4	171.98	172.16
H 2a	2.53	29.14	29.1
H 2b	2.81	29.36	29.43
H 3	4.21	27.55	27.56
H 4a	3.03	28.85	28.7
H 4b	2.83	28.95	28.91
H 5	6.51	25.08	25.06
H 9	5.31	26.38	26.39
H 10a-c	1.42	30.25	30.25
H 11a-c	2.42	29.46	29.49
J 2a,3	8	4.48	7.95
J 2b,3	4	9.34	4.07
J 4a,3	8	3.84	7.2
J 4b,3	4	8.68	4.03

3.4. Mix-*J*-DP4 results

Table S3. Mix-*J*-DP4 (AMBER) results obtained using experimental data of compound **23** *versus* isomers 1-2.

Settings	Type of data (shifts)
Default	Shielding tensors

Default settings	TMS	σ	ν
H	31.830573	0.185	14.18
C	192.29325	2.306	11.38
J	-	0.992	3.06
Slope scaling J		0.9509	
Intercept scaling J		-0.1405	

Custom settings	TMS	σ	ν
H	31.830573	0.185	14.18
C	192.29325	2.306	11.38
J	-	0.992	3.06
Slope scaling J		0.9509	
Intercept scaling J		-0.1405	

Most Likely Isomers		
Rank	Isomer	Probability
1º	2	100.00
2º	1	0.00
3º	-	-
4º	-	-

Isomer Nº		1	2	3	4	5	6	7	8	9	10	11	12	13	14	15
DP4 (%)	H	52.20	47.80	-	-	-	-	-	-	-	-	-	-	-	-	-
	C	11.36	88.64	-	-	-	-	-	-	-	-	-	-	-	-	-
	H+C	*12.28*	*87.72*	-	-	-	-	-	-	-	-	-	-	-	-	-
	J	0.00	100.00	-	-	-	-	-	-	-	-	-	-	-	-	-
	**all data**	**0.00**	**100.00**	-	-	-	-	-	-	-	-	-	-	-	-	-
**Type**	**Exp**	**1**	**2**	**3**	**4**	**5**	**6**	**7**	**8**	**9**	**10**	**11**	**12**	**13**	**14**	**15**

Table S4. Mix-*J*-DP4 (MM3) results obtained using experimental data of compound **23** *versus* isomers 1-2.

Settings	Type of data (shifts)
Default	Shielding tensors

Default settings	TMS	σ	ν
H	31.830573	0.185	14.18
C	192.29325	2.306	11.38
J	-	0.992	3.06
Slope scaling J		0.9509	
Intercept scaling J		-0.1405	

Custom settings	TMS	σ	ν
H	31.830573	0.185	14.18
C	192.29325	2.306	11.38
J	-	0.992	3.06
Slope scaling J		0.9509	
Intercept scaling J		-0.1405	

Most Likely Isomers		
Rank	Isomer	Probability
1º	2	100.00
2º	1	0.00
3º	-	-
4º	-	-

Isomer Nº		1	2	3	4	5	6	7	8	9	10	11	12	13	14	15
DP4 (%)	H	8.15	91.85	-	-	-	-	-	-	-	-	-	-	-	-	-
	C	14.60	85.40	-	-	-	-	-	-	-	-	-	-	-	-	-
	H+C	*1.49*	*98.51*	-	-	-	-	-	-	-	-	-	-	-	-	-
	J	0.00	100.00	-	-	-	-	-	-	-	-	-	-	-	-	-
	**all data**	**0.00**	**100.00**	-	-	-	-	-	-	-	-	-	-	-	-	-
**Type**	**Exp**	**1**	**2**	**3**	**4**	**5**	**6**	**7**	**8**	**9**	**10**	**11**	**12**	**13**	**14**	**15**

Table S5. Mix-*J*-DP4 (MMFF) results obtained using experimental data of compound **23** *versus* isomers 1-2.

Settings	Type of data (shifts)
Default	Shielding tensors

Default settings	TMS	σ	ν
H	31.830573	0.185	14.18
C	192.29325	2.306	11.38
J	-	0.992	3.06
Slope scaling J		0.9509	
Intercept scaling J		-0.1405	

Custom settings	TMS	σ	ν
H	31.830573	0.185	14.18
C	192.29325	2.306	11.38
J	-	0.992	3.06
Slope scaling J		0.9509	
Intercept scaling J		-0.1405	

Most Likely Isomers		
Rank	Isomer	Probability
1º	2	100.00
2º	1	0.00
3º	-	-
4º	-	-

Isomer Nº		1	2	3	4	5	6	7	8	9	10	11	12	13	14	15
DP4 (%)	H	77.12	22.88	-	-	-	-	-	-	-	-	-	-	-	-	-
	C	22.17	77.83	-	-	-	-	-	-	-	-	-	-	-	-	-
	H+C	*48.99*	*51.01*	-	-	-	-	-	-	-	-	-	-	-	-	-
	J	0.00	100.00	-	-	-	-	-	-	-	-	-	-	-	-	-
	**all data**	**0.00**	**100.00**	-	-	-	-	-	-	-	-	-	-	-	-	-
**Type**	**Exp**	**1**	**2**	**3**	**4**	**5**	**6**	**7**	**8**	**9**	**10**	**11**	**12**	**13**	**14**	**15**

3.5. CMAE for ^{1}H and ^{13}C for the isomers of compounds 23.

Table S6. CMAE values for isomers of compound **23** computed at the B3LYP/6-31G** level of theory, using AMBER as force field.

	Absoluted Error Scaled	
	**Isomer1**	**Isomer2**
C 1	3.57	2.58
C 2	1.43	0.64
C 3	1.34	0.86
C 4	0.30	1.77
C 4a	1.83	0.63
C 5	2.90	2.67
C 6	0.76	0.73
C 7	0.37	0.19
C 8	4.70	3.55
C 8a	1.83	2.27
C 9	1.64	1.74
C 10	2.36	2.09
C 11	0.36	0.34
**Average**	**1.8**	**1.5**
H 2a	0.17	0.15
H 2b	0.43	0.42
H 3	0.01	0.01
H 4a	0.07	0.03
H 4b	0.03	0.07
H 5	0.06	0.06
H 9	0.05	0.03
H 10a-c	0.23	0.22
H 11a-c	0.06	0.14
**Average**	**0.1**	**0.1**

Table S7. CMAE values for isomers of compound **23** computed at the B3LYP/6-31G** level of theory, using MM3 as force field.

	Absoluted Error Scaled	
	**Isomer1**	**Isomer2**
C 1	2.84	2.85
C 2	2.21	0.79
C 3	2.81	1.81
C 4	0.42	0.32
C 4a	1.73	1.39
C 5	0.55	0.11
C 6	1.14	1.03
C 7	2.14	1.94
C 8	4.13	4.23
C 8a	0.97	0.61
C 9	1.61	1.52
C 10	2.64	2.88
C 11	0.14	0.28
**Average**	**1.8**	**1.5**
H 2a	0.24	0.07
H 2b	0.43	0.28
H 3	0.08	0.13
H 4a	0.04	0.05
H 4b	0.08	0.07
H 5	0.03	0.04
H 9	0.11	0.09
H 10a-c	0.16	0.16
H 11a-c	0.06	0.07
**Average**	**0.1**	**0.1**

Table S8. CMAE values for isomers of compound **23** computed at the B3LYP/6-31G** level of theory, using MMFF as force field.

	Absoluted Error Scaled	
	**Isomer1**	**Isomer2**
C 1	1.41	1.12
C 2	3.10	1.37
C 3	1.37	0.91
C 4	0.54	1.95
C 4a	0.21	0.53
C 5	0.62	0.27
C 6	1.76	1.61
C 7	3.27	3.06
C 8	4.77	4.20
C 8a	1.76	1.96
C 9	3.71	3.83
C 10	1.98	1.82
C 11	0.86	0.82
**Average**	**2.0**	**1.8**
H 2a	0.15	0.17
H 2b	0.34	0.42
H 3	0.00	0.03
H 4a	0.07	0.06
H 4b	0.03	0.06
H 5	0.07	0.08
H 9	0.02	0.00
H 10a-c	0.19	0.18
H 11a-c	0.05	0.09
**Average**	**0.1**	**0.1**

3.6. Correlation plots of compound 23.

Figure S73. Compound **23** correlation plots between isotropic magnetic shielding and experimental chemical shift for ^{13}C and ^{1}H for the most probable isomer computed at B3LYP/6-31G** level of theory, using AMBER.

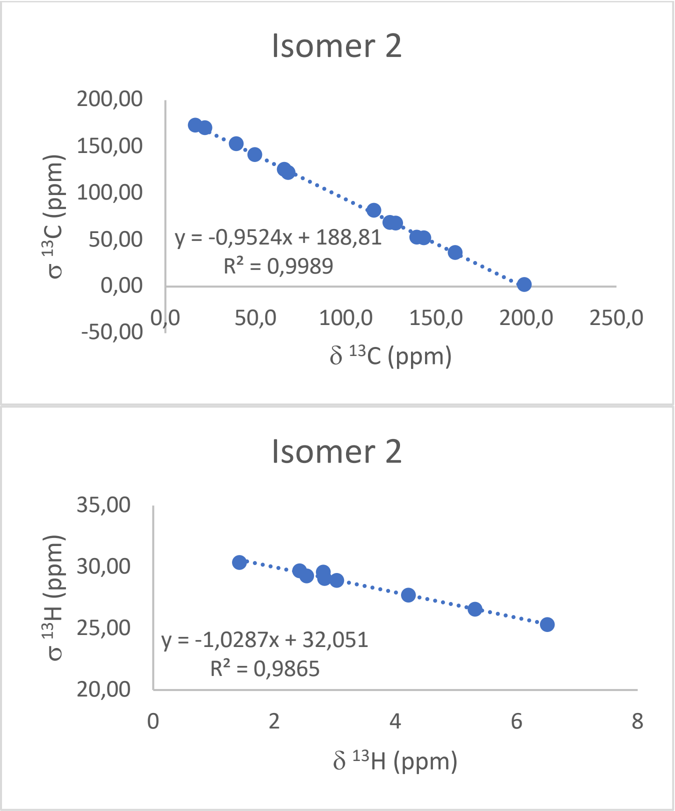

Figure S74. Compound **23** correlation plots between isotropic magnetic shielding and experimental chemical shift for ^{13}C and ^{1}H for the most probable isomer computed at B3LYP/6-31G** level of theory, using MM3.

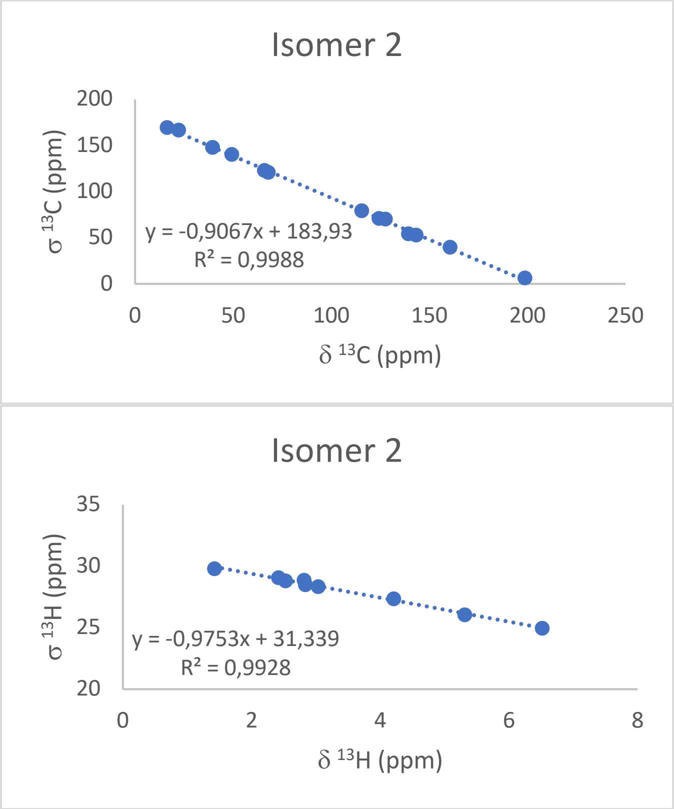

Figure S75. Compound **23** correlation plots between isotropic magnetic shielding and experimental chemical shift for ^{13}C and ^{1}H for the most probable isomer computed at B3LYP/6-31G** level of theory, using MMFF.

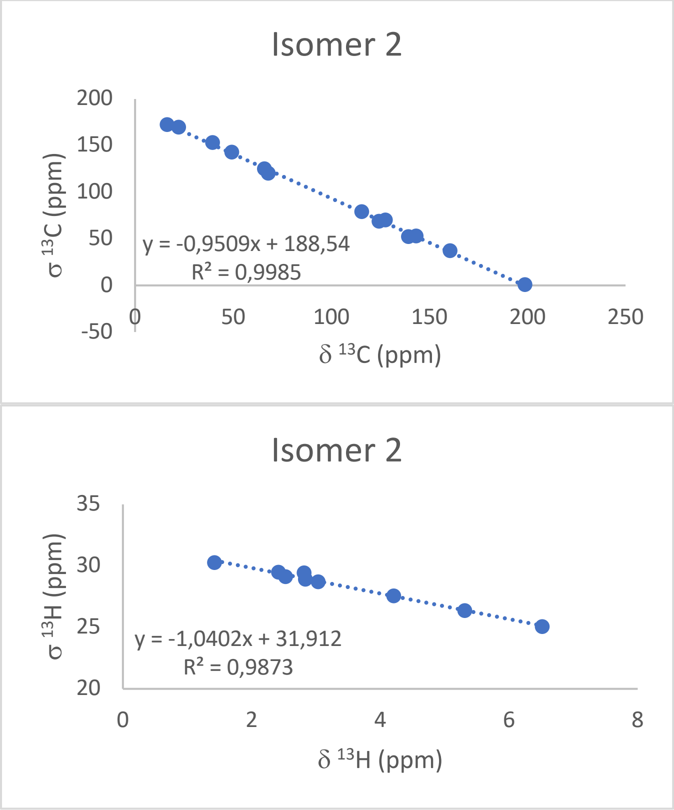

3.7. SCF energies of compound 23.

Table S9. SCF energies (Hartree) computed at B3LYP/6-31G** level of theory, using AMBER as force field, for the coordinate files of compound **23**.

Name	Energy (Hartree)
**01-Compound23-RS_miniAMBER_1**	-805.9311170
**01-Compound23-RS_miniAMBER_10**	-805.9368753
**01-Compound23-RS_miniAMBER_2**	-805.9329078
**01-Compound23-RS_miniAMBER_3**	-805.9328140
**01-Compound23-RS_miniAMBER_4**	-805.9338721
**01-Compound23-RS_miniAMBER_5**	-805.9336763
**01-Compound23-RS_miniAMBER_6**	-805.9367073
**01-Compound23-RS_miniAMBER_7**	-805.9330901
**01-Compound23-RS_miniAMBER_8**	-805.9358657
**01-Compound23-RS_miniAMBER_9**	-805.9371970
**02-Compound23-RR_miniAMBER_11**	-805.9311850
**02-Compound23-RR_miniAMBER_12**	-805.9311981
**02-Compound23-RR_miniAMBER_13**	-805.9330584
**02-Compound23-RR_miniAMBER_14**	-805.9343149
**02-Compound23-RR_miniAMBER_15**	-805.9337675
**02-Compound23-RR_miniAMBER_16**	-805.9344043
**02-Compound23-RR_miniAMBER_17**	-805.9332122
**02-Compound23-RR_miniAMBER_18**	-805.9360618
**02-Compound23-RR_miniAMBER_19**	-805.9369033
**02-Compound23-RR_miniAMBER_20**	-805.9328128

Table S10. SCF energies (Hartree) computed at B3LYP/6-31G** level of theory, using MM3 as force field, for the coordinate files of compound **23**.

Name	Energy (Hartree)
**01-Compound23-RS_miniMM3_1**	-805.92981769
**01-Compound23-RS_miniMM3_10**	-805.93409913
**01-Compound23-RS_miniMM3_2**	-805.93422714
**01-Compound23-RS_miniMM3_3**	-805.93107974
**01-Compound23-RS_miniMM3_4**	-805.93392135
**01-Compound23-RS_miniMM3_5**	-805.93410759
**01-Compound23-RS_miniMM3_6**	-805.93421556
**01-Compound23-RS_miniMM3_7**	-805.93320025
**01-Compound23-RS_miniMM3_8**	-805.93323595
**01-Compound23-RS_miniMM3_9**	-805.93392428
**02-Compound23-RR_miniMM3_11**	-805.93292794
**02-Compound23-RR_miniMM3_12**	-805.93215421
**02-Compound23-RR_miniMM3_13**	-805.93349179
**02-Compound23-RR_miniMM3_14**	-805.93291770
**02-Compound23-RR_miniMM3_15**	-805.93433168
**02-Compound23-RR_miniMM3_16**	-805.93220547
**02-Compound23-RR_miniMM3_17**	-805.93398134
**02-Compound23-RR_miniMM3_18**	-805.93348933
**02-Compound23-RR_miniMM3_19**	-805.93434654
**02-Compound23-RR_miniMM3_20**	-805.93361437

Table S11. SCF energies (Hartree) computed at B3LYP/6-31G** level of theory, using MMFF as force field, for the coordinate files of compound **23**.

Name	Energy (Hartree)
**01-Compound23-RS_miniMMFF_1**	-805.93186860
**01-Compound23-RS_miniMMFF_10**	-805.93819093
**01-Compound23-RS_miniMMFF_2**	-805.93170282
**01-Compound23-RS_miniMMFF_3**	-805.93354553
**01-Compound23-RS_miniMMFF_4**	-805.93467483
**01-Compound23-RS_miniMMFF_5**	-805.93473107
**01-Compound23-RS_miniMMFF_6**	-805.93722148
**01-Compound23-RS_miniMMFF_7**	-805.93433104
**01-Compound23-RS_miniMMFF_8**	-805.93737579
**01-Compound23-RS_miniMMFF_9**	-805.93834344
**02-Compound23-RR_miniMMFF_11**	-805.93295769
**02-Compound23-RR_miniMMFF_12**	-805.93241529
**02-Compound23-RR_miniMMFF_13**	-805.93439175
**02-Compound23-RR_miniMMFF_14**	-805.93631215
**02-Compound23-RR_miniMMFF_15**	-805.93471171
**02-Compound23-RR_miniMMFF_16**	-805.93574788
**02-Compound23-RR_miniMMFF_17**	-805.93458595
**02-Compound23-RR_miniMMFF_18**	-805.93755607
**02-Compound23-RR_miniMMFF_19**	-805.93813336
**02-Compound23-RR_miniMMFF_20**	-805.93369430

3.8. Cartesian coordinates of conformers for compound 23.

Table S12. Cartesian coordinates of the conformations found for isomers of compound **23** for AMBER, MM3 and MMFF as required for mix-J-DP4 analysis.

01-Compound23-RS_miniAMBER_1			
O	-3.51790	-0.78630	1.35970
C	-3.52380	-0.72580	-0.05150
C	-3.35710	0.71200	-0.54150
C	-2.37110	-1.55410	-0.60930
C	-0.99440	-0.96540	-0.36160
C	-0.80980	0.42260	-0.16730
C	0.52100	0.93020	-0.01380
C	0.77140	2.43580	0.04480
C	1.62210	0.03060	0.06230
C	3.04770	0.53540	0.31430
C	3.83050	0.82030	-0.97030
O	3.81660	-0.36520	1.09370
C	1.40130	-1.35370	-0.11730
O	2.42650	-2.25400	-0.07900
C	0.10740	-1.83000	-0.34430
C	-2.01390	1.30190	-0.12080
O	-1.97790	2.45910	0.26850
H	3.01180	1.44050	0.91930
H	-3.67020	-1.69340	1.63740
H	-4.47260	-1.12270	-0.41580
H	-3.41690	0.72890	-1.62990
H	-4.16450	1.32510	-0.13890
H	-2.42570	-2.55560	-0.17990
H	-2.50250	-1.64300	-1.68870
H	0.15100	2.94670	-0.69060
H	0.54060	2.80920	1.04310
H	1.78980	2.71230	-0.21250
H	4.81380	1.22210	-0.72430
H	3.95340	-0.10170	-1.53980
H	3.29590	1.54460	-1.58490
H	4.50260	0.13240	1.54990
H	3.23200	-1.84260	0.26280
H	-0.03970	-2.89040	-0.49230
33			
01-Compound23-RS_miniAMBER_2			
O	-3.53910	-0.78360	1.34310
C	-3.51850	-0.75710	-0.06980
C	-3.33830	0.66320	-0.60360
C	-2.35040	-1.60150	-0.57150
C	-0.97800	-0.99590	-0.33510
C	-0.79970	0.39690	-0.16750
C	0.51870	0.92140	-0.02580
C	0.75090	2.43060	0.00600
C	1.62930	0.03540	0.06360
C	3.04970	0.56160	0.29950
C	3.82740	0.82070	-0.99340
O	3.82830	-0.31150	1.10040
C	1.42250	-1.35450	-0.08750
O	2.45800	-2.24190	-0.03300
C	0.13260	-1.84870	-0.30170
C	-2.01690	1.26270	-0.13530
O	-2.00830	2.40670	0.29280
H	3.00510	1.48260	0.87930
H	-4.27460	-0.25260	1.66130
H	-4.45470	-1.17470	-0.44350
H	-3.34480	0.64190	-1.69370
H	-4.16440	1.28850	-0.26270
H	-2.40580	-2.57960	-0.09090
H	-2.46670	-1.74660	-1.64630
H	0.10130	2.92660	-0.71430
H	0.54770	2.81330	1.00670
H	1.75830	2.71200	-0.28750
H	3.28490	1.52420	-1.62520
H	4.80740	1.23800	-0.75970
H	3.95830	-0.11420	-1.53950
H	4.50850	0.20420	1.54500
H	3.25790	-1.81550	0.30340
H	-0.00640	-2.91330	-0.42590
33			
01-Compound23-RS_miniAMBER_3			
O	-3.70530	-0.76150	1.28490
C	-3.54020	-0.73450	-0.12000
C	-3.32030	0.68460	-0.64370
C	-2.35470	-1.60580	-0.53200
C	-0.98130	-0.99820	-0.30580
C	-0.79960	0.39520	-0.15400
C	0.51900	0.92160	-0.03010
C	0.75060	2.43060	-0.01720
C	1.62990	0.03720	0.05980
C	3.05190	0.56680	0.27370
C	3.82340	0.78870	-1.02980
O	3.82910	-0.28950	1.09360
C	1.42090	-1.35450	-0.06910
O	2.45690	-2.24090	-0.00760
C	0.12910	-1.85120	-0.26830
C	-2.01250	1.26140	-0.11490
O	-2.00970	2.37290	0.39000
H	3.01320	1.50460	0.82670
H	-2.94940	-0.35120	1.71340
H	-4.44780	-1.14330	-0.56700
H	-3.27810	0.66410	-1.73300
H	-4.15390	1.31820	-0.33770
H	-2.42720	-2.55780	-0.00320
H	-2.44360	-1.80920	-1.60020
H	0.06280	2.92750	-0.70010
H	0.60540	2.81690	0.99220
H	1.74200	2.70440	-0.36850
H	4.80640	1.20790	-0.81260
H	3.94720	-0.16030	-1.55270
H	3.27990	1.47830	-1.67600
H	4.52910	0.22880	1.50300
H	3.25950	-1.80990	0.31670
H	-0.01030	-2.91750	-0.38040
33			
01-Compound23-RS_miniAMBER_4			
O	4.65210	-1.11840	0.43110
C	3.35800	-0.55490	0.50750
C	3.30720	0.79380	-0.20650
C	2.36620	-1.47890	-0.18830
C	0.95440	-0.93520	-0.16040
C	0.71500	0.45690	-0.15610
C	-0.62720	0.93130	-0.05110
C	-0.90980	2.42360	0.11200
C	-1.71120	0.00780	-0.07600
C	-3.16750	0.48740	-0.05270
C	-3.75320	0.59100	1.35800
O	-4.02850	-0.34140	-0.81490
C	-1.44140	-1.37960	-0.10450
O	-2.44420	-2.30540	-0.10630
C	-0.11970	-1.83280	-0.12830
C	1.89410	1.37590	-0.27250
O	1.77730	2.57410	-0.47290
H	-3.24010	1.45760	-0.54240
H	5.26850	-0.56360	0.91660
H	3.07430	-0.43470	1.55480
H	3.95940	1.50030	0.30810
H	3.66870	0.66860	-1.22780
H	2.66720	-1.62150	-1.22730
H	2.39510	-2.44720	0.31390
H	-1.88770	2.63290	0.53570
H	-0.20410	2.86200	0.81660
H	-0.82460	2.92330	-0.85350
H	-4.76980	0.98270	1.31030
H	-3.77170	-0.39510	1.82350
H	-3.14590	1.25760	1.97050
H	-4.80140	0.17160	-1.07130
H	-3.29990	-1.88710	-0.27200
H	0.06920	-2.89720	-0.13960
33			
01-Compound23-RS_miniAMBER_5			
O	4.65990	-1.05940	0.45540
C	3.35300	-0.52690	0.52070
C	3.30570	0.84370	-0.15230
C	2.38490	-1.44340	-0.21770
C	0.96730	-0.91610	-0.18230
C	0.71000	0.47350	-0.15990
C	-0.62740	0.93660	-0.04160
C	-0.92050	2.42370	0.14450
C	-1.70440	0.00560	-0.07400
C	-3.16340	0.47460	-0.03600
C	-3.74670	0.54080	1.37790
O	-4.01840	-0.34440	-0.81540
C	-1.42470	-1.37910	-0.12420
O	-2.42150	-2.31120	-0.13630
C	-0.09980	-1.82260	-0.15910
C	1.88660	1.40340	-0.27420
O	1.75460	2.59070	-0.52470
H	-3.24360	1.45630	-0.50120
H	4.67320	-1.91400	0.89410
H	3.05140	-0.43150	1.56550
H	3.91890	1.54390	0.41580
H	3.72050	0.75810	-1.15730
H	2.69730	-1.54260	-1.25850
H	2.41910	-2.42870	0.24940
H	-0.19470	2.86720	0.82520
H	-0.87790	2.93200	-0.81940
H	-1.88520	2.61360	0.60640
H	-3.75650	-0.45590	1.82070
H	-3.14310	1.19800	2.00420
H	-4.76640	0.92540	1.34130
H	-4.79100	0.17100	-1.06810
H	-3.28000	-1.89560	-0.29500
H	0.09520	-2.88550	-0.18610
33			
01-Compound23-RS_miniAMBER_6			
O	-3.52440	-0.79530	1.34150
C	-3.50570	-0.77130	-0.07150
C	-3.33510	0.64950	-0.60760
C	-2.33260	-1.60980	-0.57170
C	-0.96360	-0.99440	-0.34020
C	-0.79500	0.40020	-0.17680
C	0.51980	0.93480	-0.04290
C	0.74720	2.44440	-0.02450
C	1.63490	0.05650	0.05100
C	3.05200	0.58340	0.29350
C	3.86280	0.77000	-0.99120
O	3.78790	-0.26980	1.15300
C	1.44000	-1.33450	-0.09860
O	2.48730	-2.20950	-0.04880
C	0.15350	-1.83950	-0.30810
C	-2.01770	1.25680	-0.13810
O	-2.01630	2.39650	0.30090
H	3.01780	1.53350	0.82480
H	-4.26550	-0.27160	1.65920
H	-4.43950	-1.19590	-0.44350
H	-3.34040	0.62670	-1.69780
H	-4.16530	1.26990	-0.26800
H	-2.38080	-2.58670	-0.08770
H	-2.44970	-1.75960	-1.64580
H	1.74400	2.72040	-0.35900
H	0.07310	2.93870	-0.72270
H	0.58150	2.83160	0.98130
H	4.84800	1.17000	-0.74890
H	3.98170	-0.18660	-1.50090
H	3.35190	1.46540	-1.65750
H	3.48030	-0.13470	2.05540
H	3.30210	-1.76800	0.22940
H	0.02210	-2.90530	-0.43150
33			
01-Compound23-RS_miniAMBER_7			
O	4.63090	-1.09800	0.55900
C	3.32630	-0.55410	0.56970
C	3.30620	0.83890	-0.06030
C	2.38060	-1.45190	-0.21920
C	0.96270	-0.92680	-0.18470
C	0.71920	0.46310	-0.16070
C	-0.62100	0.93420	-0.04150
C	-0.90370	2.42310	0.14410
C	-1.70300	0.00970	-0.07280
C	-3.15850	0.48800	-0.02950
C	-3.74190	0.54080	1.38490
O	-4.01670	-0.31760	-0.81930
C	-1.43140	-1.37670	-0.12680
O	-2.43500	-2.30150	-0.14190
C	-0.10880	-1.82820	-0.16260
C	1.89230	1.38570	-0.27630
O	1.76900	2.55300	-0.61160
H	-3.23170	1.47680	-0.48060
H	4.92290	-1.20900	-0.34940
H	2.98290	-0.48590	1.60370
H	3.86170	1.52800	0.57650
H	3.79610	0.79560	-1.03340
H	2.70890	-1.51640	-1.25750
H	2.41210	-2.45080	0.21890
H	-0.15140	2.87400	0.79010
H	-0.90040	2.92260	-0.82530
H	-1.85050	2.61250	0.64290
H	-4.75940	0.93140	1.35270
H	-3.75720	-0.46080	1.81650
H	-3.13490	1.18760	2.01850
H	-4.79040	0.20200	-1.05990
H	-3.29020	-1.87850	-0.29980
H	0.08120	-2.89230	-0.18910
33			
01-Compound23-RS_miniAMBER_8			
O	-3.69780	-0.77060	1.28110
C	-3.52880	-0.74780	-0.12350
C	-3.31570	0.67120	-0.65090
C	-2.33730	-1.61480	-0.52830
C	-0.96720	-0.99790	-0.30740
C	-0.79440	0.39740	-0.16150
C	0.52080	0.93320	-0.04490
C	0.74730	2.44250	-0.04340
C	1.63610	0.05610	0.04850
C	3.05570	0.58700	0.26480
C	3.85320	0.74290	-1.03210
O	3.79810	-0.24950	1.13510
C	1.43810	-1.33690	-0.07720
O	2.48490	-2.21180	-0.02080
C	0.14920	-1.84360	-0.27000
C	-2.01250	1.25500	-0.11900
O	-2.01690	2.36170	0.39620
H	3.02790	1.55040	0.77230
H	-2.95460	-0.33790	1.70970
H	-4.43250	-1.16390	-0.57140
H	-3.27030	0.64810	-1.74010
H	-4.15350	1.30120	-0.34870
H	-2.40420	-2.56330	0.00740
H	-2.42510	-1.82660	-1.59500
H	0.03840	2.93820	-0.70530
H	0.63570	2.83260	0.96880
H	1.72740	2.71170	-0.43030
H	4.84160	1.14610	-0.80930
H	3.96530	-0.22510	-1.52150
H	3.33670	1.42430	-1.70840
H	3.53480	-0.06120	2.04180
H	3.30240	-1.76750	0.24500
H	0.01670	-2.91110	-0.37990
33			
01-Compound23-RS_miniAMBER_9			
O	4.63080	-1.14120	0.44210
C	3.33810	-0.57430	0.51340
C	3.30530	0.79520	-0.16320
C	2.35260	-1.47690	-0.21930
C	0.94230	-0.93200	-0.17600
C	0.71150	0.46100	-0.15280
C	-0.62520	0.94270	-0.02530
C	-0.90060	2.43150	0.16980
C	-1.71390	0.02650	-0.05620
C	-3.16760	0.50720	-0.02190
C	-3.77560	0.51250	1.38260
O	-4.00080	-0.28130	-0.85390
C	-1.45540	-1.36160	-0.10360
O	-2.46960	-2.27600	-0.10350
C	-0.13700	-1.82410	-0.14570
C	1.89310	1.37370	-0.27690
O	1.77700	2.56190	-0.53060
H	-3.24660	1.50980	-0.43970
H	5.24190	-0.60280	0.95200
H	3.03920	-0.48120	1.55920
H	3.93050	1.49070	0.39750
H	3.71040	0.70050	-1.17150
H	2.65860	-1.58360	-1.26120
H	2.37940	-2.46160	0.25010
H	-0.15300	2.87180	0.82840
H	-0.88690	2.94110	-0.79410
H	-1.84930	2.62130	0.66520
H	-4.79860	0.88780	1.33900
H	-3.78390	-0.49890	1.79020
H	-3.19020	1.15480	2.04090
H	-3.86980	-0.00830	-1.76780
H	-3.33180	-1.84770	-0.20060
H	0.04510	-2.88970	-0.16960
33			
01-Compound23-RS_miniAMBER_10			
O	4.64300	-1.07980	0.45780
C	3.33750	-0.54380	0.52280
C	3.30220	0.84170	-0.12140
C	2.37130	-1.44310	-0.23970
C	0.95530	-0.91310	-0.19140
C	0.71310	0.47730	-0.15380
C	-0.62640	0.94720	-0.01800
C	-0.91300	2.43160	0.19290
C	-1.70790	0.02310	-0.05540
C	-3.16480	0.49240	-0.01050
C	-3.77000	0.47010	1.39510
O	-3.99270	-0.29000	-0.85360
C	-1.43870	-1.36240	-0.11970
O	-2.44610	-2.28420	-0.12850
C	-0.11680	-1.81430	-0.16940
C	1.88550	1.40090	-0.27350
O	1.75610	2.58060	-0.55930
H	-3.25180	1.50160	-0.41060
H	4.65080	-1.94130	0.88330
H	3.02820	-0.46830	1.56700
H	3.89700	1.53170	0.47810
H	3.74510	0.77890	-1.11600
H	2.68520	-1.51720	-1.28210
H	2.40360	-2.43930	0.20380
H	-0.15570	2.87740	0.83640
H	-0.92740	2.94700	-0.76790
H	-1.85290	2.60530	0.71090
H	-4.79590	0.83820	1.35930
H	-3.76990	-0.54760	1.78650
H	-3.18840	1.10650	2.06240
H	-3.87480	0.00740	-1.76170
H	-3.31110	-1.86060	-0.22190
H	0.07180	-2.87830	-0.20600
33			
02-Compound23-RR_miniAMBER_11			
O	3.04280	-0.89910	1.67100
C	3.41200	-0.77560	0.31190
C	3.42750	0.69880	-0.10790
C	2.41460	-1.53600	-0.55790
C	1.01800	-0.97310	-0.41630
C	0.83820	0.41790	-0.28710
C	-0.46790	0.93970	-0.07120
C	-0.66860	2.42630	0.21110
C	-1.59080	0.06670	-0.09900
C	-3.01620	0.60480	0.06120
C	-3.52120	0.57230	1.50600
O	-3.95170	-0.09880	-0.73870
C	-1.38750	-1.32370	-0.25910
O	-2.43460	-2.19900	-0.28040
C	-0.09050	-1.82940	-0.39770
C	2.04430	1.28840	-0.40050
O	1.96940	2.43950	-0.80370
H	-3.06230	1.62990	-0.30470
H	3.09410	-1.82410	1.92670
H	4.41250	-1.19200	0.18440
H	3.91410	1.29150	0.66740
H	4.01460	0.78580	-1.02270
H	2.71880	-1.46360	-1.60310
H	2.42580	-2.58740	-0.26770
H	-0.76270	2.96930	-0.72980
H	-1.54470	2.61330	0.82730
H	0.17090	2.82670	0.77790
H	-4.51900	1.00850	1.56070
H	-3.56150	-0.45800	1.86130
H	-2.85070	1.14100	2.15070
H	-4.70340	0.47710	-0.90940
H	-3.27390	-1.72550	-0.36170
H	0.04860	-2.89570	-0.50430
33			
02-Compound23-RR_miniAMBER_12			
O	3.05930	-0.87550	1.66740
C	3.41210	-0.80300	0.29970
C	3.43360	0.64800	-0.19120
C	2.39250	-1.58380	-0.52410
C	1.00300	-1.00140	-0.39180
C	0.83450	0.39430	-0.29310
C	-0.46880	0.92900	-0.08550
C	-0.65930	2.42160	0.17200
C	-1.59850	0.06380	-0.09760
C	-3.02070	0.61500	0.04800
C	-3.52760	0.62070	1.49250
O	-3.96100	-0.09990	-0.73600
C	-1.40550	-1.33090	-0.22790
O	-2.45870	-2.19900	-0.23220
C	-0.11200	-1.84820	-0.35360
C	2.04750	1.25530	-0.42670
O	1.97730	2.41790	-0.79530
H	-3.06020	1.63070	-0.34380
H	3.75300	-0.47100	2.19570
H	4.40020	-1.24720	0.17000
H	3.98100	1.26710	0.52010
H	3.96300	0.67540	-1.14430
H	2.68430	-1.56550	-1.57510
H	2.39510	-2.62010	-0.18290
H	-0.72200	2.95380	-0.77760
H	-1.54740	2.63030	0.76330
H	0.17030	2.81730	0.75660
H	-3.57560	-0.40070	1.87190
H	-2.85370	1.19950	2.12450
H	-4.52230	1.06520	1.53540
H	-4.71220	0.47460	-0.91370
H	-3.29470	-1.72180	-0.32310
H	0.02130	-2.91760	-0.43360
33			
02-Compound23-RR_miniAMBER_13			
O	3.12400	-0.93760	1.68200
C	3.42360	-0.79760	0.30560
C	3.43590	0.67430	-0.12340
C	2.40640	-1.56570	-0.53350
C	1.01490	-0.99090	-0.39770
C	0.84230	0.40300	-0.28830
C	-0.46280	0.93340	-0.08210

C -0.65700 2.42390 0.18330
C -1.59010 0.06570 -0.10130
C -3.01380 0.61260 0.04410
C -3.52540 0.60660 1.48700
O -3.94790 -0.10140 -0.74760
C -1.39260 -1.32780 -0.23820
O -2.44290 -2.19910 -0.24730
C -0.09750 -1.84120 -0.36560
C 2.05290 1.26800 -0.40930
O 1.98040 2.42240 -0.80030
H -3.05490 1.63110 -0.34050
H 2.23470 -0.62180 1.85870
H 4.41750 -1.21650 0.14040
H 3.93590 1.27130 0.64010
H 4.01260 0.75140 -1.04580
H 2.70150 -1.52240 -1.58300
H 2.41410 -2.60980 -0.21680
H -0.73040 2.95910 -0.76390
H -1.54220 2.62520 0.78180
H 0.17530 2.82300 0.76180
H -4.52180 1.04760 1.52930
H -3.57190 -0.41750 1.85920
H -2.85610 1.18350 2.12540
H -4.70030 0.47140 -0.92670
H -3.28100 -1.72610 -0.34300
H 0.03760 -2.91010 -0.45570
33
02-Compound23-RR_miniAMBER_14
O 3.03660 -0.93040 1.65770
C 3.40230 -0.79010 0.29950
C 3.42360 0.69060 -0.09880
C 2.39800 -1.53490 -0.57650
C 1.00480 -0.96780 -0.42080
C 0.83370 0.42290 -0.27640
C -0.46760 0.94990 -0.04630
C -0.66220 2.43280 0.25670
C -1.59450 0.08350 -0.07880
C -3.01820 0.62060 0.08550
C -3.55030 0.51050 1.51620
O -3.92770 -0.04710 -0.77220
C -1.40150 -1.30620 -0.25090
O -2.45720 -2.17230 -0.26760
C -0.10850 -1.81840 -0.40330
C 2.04330 1.28700 -0.39140
O 1.97220 2.43680 -0.79890
H -3.06740 1.66500 -0.22020
H 3.09030 -1.85820 1.90270
H 4.40050 -1.20900 0.16330
H 3.90670 1.27100 0.68770
H 4.01640 0.78950 -1.00870
H 2.69830 -1.44980 -1.62190
H 2.40590 -2.59020 -0.30040
H -0.79370 2.98530 -0.67400
H -1.51700 2.60370 0.90750
H 0.19390 2.83360 0.79780
H -4.55510 0.93110 1.56800
H -3.58490 -0.53510 1.82400
H -2.90050 1.05930 2.19820
H -3.82610 0.30690 -1.66160
H -3.30010 -1.69720 -0.28300
H 0.02420 -2.88470 -0.51900
33
02-Compound23-RR_miniAMBER_15
O 4.72020 -1.06640 0.10330
C 3.46690 -0.53350 -0.27560
C 3.22660 0.82220 0.38370
C 2.36170 -1.48810 0.17490
C 0.95060 -0.93440 0.07520
C 0.70360 0.45510 0.00380
C -0.63890 0.93110 -0.01070
C -0.92770 2.42840 0.05890
C -1.71770 0.00540 -0.06820
C -3.17030 0.48390 -0.16220
C -3.85930 0.61360 1.19880
O -3.96750 -0.36590 -0.96940
C -1.44610 -1.38100 -0.01860
O -2.44890 -2.30610 -0.05390
C -0.12520 -1.83140 0.07140
C 1.87800 1.36920 -0.06620
O 1.81210 2.49470 -0.53540
H -3.20540 1.44720 -0.66980
H 5.41930 -0.51180 -0.25330
H 3.43210 -0.42370 -1.36120
H 3.22780 0.71330 1.46880
H 4.01650 1.51680 0.09520
H 2.43660 -2.39870 -0.42150
H 2.53640 -1.75200 1.21920
H -0.20890 2.92640 0.70840
H -0.87510 2.86000 -0.94110
H -1.89960 2.64550 0.49490
H -4.87020 1.00120 1.06780
H -3.91080 -0.36260 1.68210
H -3.29970 1.29480 1.84010
H -4.71740 0.13890 -1.29990
H -3.28770 -1.89440 -0.30260
H 0.06450 -2.89450 0.12470
33
02-Compound23-RR_miniAMBER_16
O 3.04620 -0.91150 1.65590
C 3.40020 -0.81780 0.28990
C 3.42930 0.64190 -0.17570
C 2.37650 -1.58130 -0.54550
C 0.99010 -0.99530 -0.39900
C 0.83030 0.40000 -0.28370
C -0.46780 0.94010 -0.06090
C -0.65170 2.42910 0.21850
C -1.60140 0.08160 -0.07780
C -3.02190 0.63200 0.07280
C -3.55550 0.55930 1.50540
O -3.93630 -0.04840 -0.76950
C -1.41900 -1.31260 -0.22140
O -2.48090 -2.17110 -0.22080
C -0.12970 -1.83670 -0.36250
C 2.04650 1.25470 -0.41810
O 1.98030 2.41440 -0.79630
H -3.06410 1.66870 -0.25880
H 3.74330 -0.52320 2.19180
H 4.38630 -1.26460 0.15360
H 3.96840 1.24820 0.55250
H 3.97000 0.68500 -1.12170
H 2.66630 -1.54590 -1.59660
H 2.37570 -2.62330 -0.22200
H -0.75460 2.97150 -0.72180
H -1.51810 2.62160 0.84740
H 0.19650 2.82470 0.77540
H -4.55750 0.98770 1.54650
H -3.59740 -0.47850 1.83740
H -2.90250 1.11940 2.17500
H -3.81840 0.26990 -1.67020
H -3.32060 -1.69100 -0.24160
H -0.00260 -2.90610 -0.45260
33
02-Compound23-RR_miniAMBER_17
O 4.73650 -0.99920 0.12230
C 3.47710 -0.49260 -0.27060
C 3.22230 0.86320 0.38260
C 2.37890 -1.45920 0.17220
C 0.96320 -0.91620 0.07360
C 0.70400 0.47110 0.00100
C -0.64270 0.93530 -0.01190
C -0.94520 2.43000 0.05720
C -1.71350 0.00020 -0.06770
C -3.17040 0.46590 -0.16060
C -3.85940 0.58980 1.20080
O -3.96030 -0.39170 -0.96690
C -1.42980 -1.38350 -0.01730
O -2.42420 -2.31760 -0.05080
C -0.10500 -1.82250 0.07190
C 1.86950 1.39610 -0.07170
O 1.79080 2.51860 -0.54600
H -3.21440 1.42860 -0.66850
H 4.89420 -1.83120 -0.33170
H 3.45480 -0.37530 -1.35570
H 3.22400 0.75900 1.46810
H 4.00810 1.56160 0.09190
H 2.45960 -2.36690 -0.42770
H 2.55250 -1.72440 1.21640
H -0.89910 2.86140 -0.94320
H -1.91780 2.63840 0.49550
H -0.22920 2.93510 0.70450
H -4.87380 0.96820 1.07050
H -3.90170 -0.38670 1.68460
H -3.30560 1.27630 1.84130
H -4.71310 0.10700 -1.29970
H -3.26720 -1.91310 -0.29780
H 0.09230 -2.88400 0.12660
33
02-Compound23-RR_miniAMBER_18
O 3.11720 -0.97260 1.66870
C 3.41340 -0.81180 0.29390
C 3.43130 0.66750 -0.11060
C 2.39040 -1.56370 -0.55340
C 1.00190 -0.98520 -0.40360
C 0.83760 0.40830 -0.27870
C -0.46270 0.94400 -0.05860
C -0.65100 2.43090 0.22690
C -1.59370 0.08250 -0.08250
C -3.01580 0.62830 0.06780
C -3.55190 0.54800 1.49910
O -3.92600 -0.05290 -0.77810
C -1.40600 -1.31050 -0.23080
O -2.46470 -2.17290 -0.23490
C -0.11490 -1.83030 -0.37260
C 2.05150 1.26700 -0.40040
O 1.98320 2.41940 -0.79820
H -3.06050 1.66630 -0.25940
H 2.23170 -0.65150 1.85460
H 4.40500 -1.23230 0.11960
H 3.92430 1.25140 0.66740
H 4.01710 0.75920 -1.02590
H 2.68230 -1.50540 -1.60310
H 2.39540 -2.61260 -0.25270
H -0.76060 2.97560 -0.71140
H -1.51610 2.61710 0.85950
H 0.19790 2.82990 0.78050
H -4.55550 0.97280 1.53970
H -3.59130 -0.49120 1.82690
H -2.90260 1.10780 2.17250
H -3.83180 0.29170 -1.67210
H -3.30690 -1.69710 -0.26360
H 0.01410 -2.89930 -0.47170
33
02-Compound23-RR_miniAMBER_19
O 4.70760 -1.08270 0.09900
C 3.45690 -0.54200 -0.27700
C 3.22370 0.81260 0.38740
C 2.34720 -1.49370 0.16940
C 0.93840 -0.93240 0.07740
C 0.69890 0.45900 0.01270
C -0.64090 0.94240 0.00680
C -0.92620 2.43940 0.08910
C -1.72330 0.02250 -0.05380
C -3.17450 0.50050 -0.15060
C -3.88890 0.56100 1.20160
O -3.93890 -0.32650 -1.01100
C -1.46120 -1.36500 -0.00750
O -2.47370 -2.28060 -0.03640
C -0.14250 -1.82390 0.07460
C 1.87740 1.36640 -0.06100
O 1.81580 2.48960 -0.53590
H -3.21960 1.48690 -0.61020
H 5.40960 -0.52880 -0.25310
H 3.42260 -0.42750 -1.36210
H 3.22480 0.70000 1.47200
H 4.01640 1.50460 0.10070
H 2.41610 -2.40090 -0.43280
H 2.52300 -1.76500 1.21160
H -0.18940 2.93780 0.71770
H -0.90670 2.87490 -0.91040
H -1.88460 2.64900 0.55860
H -4.90570 0.93030 1.06360
H -3.92800 -0.43240 1.64930
H -3.35630 1.23270 1.87530
H -3.73560 -0.09310 -1.92250
H -3.32470 -1.85990 -0.22370
H 0.04160 -2.88820 0.12580
33
02-Compound23-RR_miniAMBER_20
O 4.73660 -1.04120 0.02480
C 3.46720 -0.51770 -0.31280
C 3.23790 0.84190 0.34200
C 2.37330 -1.47470 0.15730
C 0.96020 -0.92500 0.06370
C 0.70750 0.46410 -0.00380
C -0.63800 0.93330 -0.01060
C -0.93480 2.42890 0.06650
C -1.71280 0.00280 -0.06700
C -3.16830 0.47420 -0.15560
C -3.85280 0.60070 1.20790
O -3.96450 -0.37950 -0.95950
C -1.43470 -1.38230 -0.02140
O -2.43300 -2.31250 -0.05370
C -0.11160 -1.82690 0.06220
C 1.87740 1.38590 -0.07780
O 1.79580 2.52110 -0.51970
H -3.21000 1.43680 -0.66380
H 4.78320 -1.18150 0.97370
H 3.41020 -0.40260 -1.39700
H 3.26560 0.74400 1.42750
H 4.02020 1.53380 0.02710
H 2.44580 -2.38800 -0.43550
H 2.55480 -1.73160 1.20190
H -0.87560 2.86780 -0.93010
H -1.91070 2.64030 0.49570
H -0.22350 2.92490 0.72580
H -4.86590 0.98320 1.08060
H -3.89780 -0.37590 1.69140
H -3.29440 1.28450 1.84740
H -4.71240 0.12430 -1.29580
H -3.27480 -1.90500 -0.29900
H 0.08170 -2.88940 0.11110
33
01-Compound23-RS_miniMM3_1
O -3.16080 -0.91830 1.49130
C -3.43110 -0.79220 0.09780
C -3.40780 0.69010 -0.26150
C -2.36140 -1.51530 -0.71540
C -0.99340 -0.94040 -0.45410
C -0.81890 0.42790 -0.18920
C 0.48740 0.91730 0.07910
C 0.67310 2.39900 0.33810
C 1.60010 0.03230 0.09810
C 3.00140 0.54110 0.39590
C 3.69850 1.03480 -0.87440
O 3.79740 -0.51010 0.93540
C 1.38410 -1.32830 -0.18300
O 2.37280 -2.26320 -0.21690
C 0.10460 -1.79750 -0.45060
C -2.01940 1.32020 -0.21550
O -1.92220 2.52720 -0.23060
H 2.95350 1.35740 1.14830
H -3.25200 -1.82710 1.74980
H -4.43670 -1.21620 -0.12770
H -3.81580 0.82260 -1.29050
H -4.09300 1.24710 0.41850
H -2.38240 -2.60210 -0.47220
H -2.58930 -1.42960 -1.80250
H 0.37420 3.00260 -0.54680
H 0.07770 2.73480 1.21590
H 1.71660 2.70030 0.54980
H 4.72210 1.41530 -0.65800
H 3.79950 0.22520 -1.63170
H 3.13730 1.86580 -1.35650
H 4.38620 -0.13350 1.57640
H 3.23470 -1.91090 0.06390
H -0.03390 -2.87100 -0.66080
33
01-Compound23-RS_miniMM3_2
O -3.46890 -0.74500 1.34960
C -3.47140 -0.79290 -0.07480
C -3.30480 0.61220 -0.64390
C -2.29310 -1.65120 -0.52960
C -0.94670 -0.99810 -0.32460
C -0.79360 0.39070 -0.16050
C 0.50770 0.92940 0.01020
C 0.68960 2.43100 0.09550
C 1.63690 0.07300 0.07300
C 3.03470 0.62040 0.30470
C 3.79140 0.83580 -1.00770
O 3.77260 -0.31340 1.08070
C 1.44830 -1.30970 -0.08890
O 2.46650 -2.21400 -0.06610
C 0.17500 -1.82610 -0.29050
C -2.01350 1.25130 -0.16930
O -2.00380 2.40420 0.20100
H 2.99460 1.57780 0.86820
H -4.24600 -0.29150 1.65320
H -4.42490 -1.24530 -0.43210
H -3.30880 0.57540 -1.75720
H -4.17430 1.24280 -0.34730
H -2.32900 -2.63110 -0.00030
H -2.40530 -1.87740 -1.61540
H 0.07270 2.96490 -0.65900
H 0.42460 2.81540 1.10550
H 1.72580 2.76190 -0.11710
H 3.27000 1.56580 -1.66600
H 4.81560 1.23290 -0.82660
H 3.90100 -0.10700 -1.58830
H 3.40660 -0.29860 1.95720
H 3.32730 -1.81870 0.15860
H 0.05660 -2.91540 -0.41710
33
01-Compound23-RS_miniMM3_3
O -3.58560 -0.76570 1.33910
C -3.49300 -0.77000 -0.08430
C -3.32220 0.65150 -0.61020
C -2.31830 -1.63200 -0.54100
C -0.96890 -0.99130 -0.32260
C -0.80160 0.39510 -0.15470
C 0.50510 0.91930 0.02190
C 0.69910 2.41910 0.11730
C 1.62710 0.05150 0.08340
C 3.02930 0.59450 0.30260
C 3.73940 0.87760 -1.02390
O 3.80430 -0.37210 1.00550
C 1.42170 -1.32910 -0.07580
O 2.42520 -2.24800 -0.04680
C 0.14340 -1.83100 -0.28360
C -2.01270 1.26900 -0.15270
O -1.98260 2.41790 0.22670
H 2.98360 1.51980 0.91750
H -2.76990 -0.46630 1.72400
H -4.43730 -1.19940 -0.49140
H -3.35160 0.64930 -1.72400
H -4.17920 1.27870 -0.27290
H -2.36560 -2.61870 -0.02530
H -2.42500 -1.84260 -1.63050
H 0.10340 2.96050 -0.64910
H 0.41460 2.80160 1.12270
H 1.74220 2.74410 -0.06740
H 4.76220 1.28560 -0.86130
H 3.84490 -0.04150 -1.64260
H 3.18550 1.62220 -1.63780
H 4.42240 0.09010 1.55660
H 3.27300 -1.86650 0.24050
H 0.01550 -2.91900 -0.41270
33
01-Compound23-RS_miniMM3_4
O 4.57580 -1.15010 0.47430
C 3.25970 -0.60280 0.54000
C 3.27530 0.81240 -0.03180
C 2.33270 -1.46900 -0.30700
C 0.93040 -0.91890 -0.24640
C 0.70620 0.46650 -0.19030
C -0.62240 0.94820 -0.07720
C -0.86920 2.43550 0.07060
C -1.71460 0.04290 -0.07160
C -3.14940 0.53600 0.00280
C -3.68350 0.53390 1.43670
O -3.97320 -0.32960 -0.76580
C -1.45330 -1.33690 -0.12820
O -2.42910 -2.28640 -0.10670
C -0.14650 -1.80220 -0.20300
C 1.88180 1.38410 -0.27260
O 1.75990 2.55310 -0.56400
H -3.23640 1.56000 -0.42110
H 5.05180 -0.90870 1.26030
H 2.90030 -0.59030 1.59500
H 3.83670 1.48450 0.65760
H 3.82770 0.81810 -1.00000
H 2.67680 -1.49370 -1.36590
H 2.35710 -2.51710 0.06920
H -1.85030 2.67240 0.52950
H -0.12820 2.91310 0.74700
H -0.83290 2.95270 -0.91390
H -4.73650 0.89240 1.47950
H -3.66210 -0.48160 1.89110
H -3.08480 1.19950 2.09770
H -3.77140 -0.16970 -1.68010
H -3.32850 -1.91490 -0.14140
H 0.03130 -2.89010 -0.23880
33
01-Compound23-RS_miniMM3_5
O 4.58730 -1.10350 0.48890
C 3.25920 -0.58540 0.54530
C 3.27270 0.83640 -0.01040
C 2.34330 -1.44770 -0.31830
C 0.93730 -0.90810 -0.25470
C 0.70680 0.47570 -0.19000
C -0.62360 0.95080 -0.07280
C -0.87610 2.43610 0.08510
C -1.71160 0.04060 -0.07170
C -3.14820 0.52740 0.00800
C -3.68100 0.51150 1.44230
O -3.96880 -0.33570 -0.76700
C -1.44420 -1.33750 -0.13800
O -2.41610 -2.29120 -0.12220
C -0.13530 -1.79670 -0.21640
C 1.87790 1.39900 -0.26870
O 1.75090 2.56450 -0.57230
H -3.23970 1.55460 -0.40730
H 4.68690 -1.77340 1.15500
H 2.89570 -0.57380 1.59910
H 3.81770 1.50500 0.69520
H 3.84010 0.85700 -0.96960
H 2.69230 -1.45470 -1.37600
H 2.37240 -2.50090 0.04270
H -0.13080 2.91460 0.75600
H -0.85240 2.95830 -0.89720
H -1.85400 2.66500 0.55510
H -3.65520 -0.50760 1.88840
H -3.08460 1.17420 2.10820
H -4.73550 0.86530 1.48880
H -3.77300 -0.16280 -1.68030
H -3.31670 -1.92260 -0.15610
H 0.04660 -2.88360 -0.25970
33
01-Compound23-RS_miniMM3_6
O -3.46820 -0.74510 1.35000
C -3.47130 -0.79290 -0.07430
C -3.30490 0.61200 -0.64360
C -2.29320 -1.65120 -0.52950
C -0.94680 -0.99810 -0.32480
C -0.79360 0.39060 -0.16090
C 0.50760 0.92950 0.00980
C 0.68940 2.43110 0.09540

C 1.63690 0.07300 0.07270
C 3.03460 0.62030 0.30500
C 3.79200 0.83640 -1.00680
O 3.77220 -0.31390 1.08090
C 1.44830 -1.30970 -0.08940
O 2.46640 -2.21410 -0.06670
C 0.17490 -1.82620 -0.29080
C -2.01360 1.25130 -0.16950
O -2.00390 2.40410 0.20060
H 2.99430 1.57730 0.86910
H -4.24410 -0.28970 1.65400
H -4.42500 -1.24530 -0.43130
H -3.30920 0.57510 -1.75700
H -4.17450 1.24260 -0.34690
H -2.32900 -2.63110 -0.00010
H -2.40580 -1.87750 -1.61530
H 1.72580 2.76210 -0.11600
H 0.07310 2.96490 -0.65980
H 0.42320 2.81530 1.10520
H 4.81610 1.23330 -0.82510
H 3.90180 -0.10610 -1.58800
H 3.27090 1.56690 -1.66490
H 3.40620 -0.29920 1.95740
H 3.32730 -1.81880 0.15790
H 0.05650 -2.91540 -0.41730
33
01-Compound23-RS_miniMM3_7
O 4.57210 -1.13400 0.59080
C 3.24830 -0.60280 0.58080
C 3.26650 0.83700 0.06870
C 2.33980 -1.45790 -0.29950
C 0.93400 -0.91770 -0.24260
C 0.70770 0.46660 -0.18420
C -0.62010 0.94820 -0.07430
C -0.86560 2.43520 0.07660
C -1.71170 0.04280 -0.07260
C -3.14580 0.53690 0.00270
C -3.68350 0.52360 1.43520
O -3.96790 -0.32210 -0.77530
C -1.44970 -1.33680 -0.13290
O -2.42620 -2.28580 -0.11730
C -0.14230 -1.80210 -0.20460
C 1.88160 1.38470 -0.26460
O 1.76580 2.53270 -0.63270
H -3.23010 1.56500 -0.41230
H 4.90670 -1.20060 -0.29520
H 2.85620 -0.61360 1.62400
H 3.75400 1.49450 0.82480
H 3.88990 0.89860 -0.85330
H 2.69410 -1.45380 -1.35540
H 2.36680 -2.51460 0.05180
H -0.10350 2.91910 0.72430
H -0.86420 2.94860 -0.91060
H -1.83200 2.66840 0.56820
H -4.73620 0.88300 1.47830
H -3.66460 -0.49540 1.88140
H -3.08590 1.18320 2.10320
H -3.76750 -0.15090 -1.68770
H -3.32480 -1.91290 -0.15570
H 0.03570 -2.88990 -0.24120
33
01-Compound23-RS_miniMM3_8
O -3.60060 -0.76620 1.32250
C -3.48540 -0.77940 -0.09920
C -3.31060 0.63890 -0.63330
C -2.30090 -1.64280 -0.52870
C -0.95480 -0.99230 -0.31640
C -0.79580 0.39610 -0.15810
C 0.50730 0.93120 0.00720
C 0.69640 2.43270 0.08270
C 1.63360 0.07140 0.07130
C 3.03470 0.61620 0.29070
C 3.78350 0.81930 -1.02820
O 3.77460 -0.31580 1.06730
C 1.43930 -1.31140 -0.08080
O 2.45410 -2.21930 -0.05450
C 0.16390 -1.82430 -0.27880
C -2.01240 1.26090 -0.15190
O -1.99480 2.40090 0.25430
H 3.00090 1.57780 0.84730
H -2.80580 -0.42670 1.71730
H -4.42170 -1.21520 -0.51810
H -3.31710 0.62840 -1.74740
H -4.17620 1.26570 -0.31760
H -2.34590 -2.61920 0.00610
H -2.39900 -1.87620 -1.61460
H 0.06220 2.96700 -0.65700
H 0.45920 2.82120 1.09800
H 1.72840 2.75770 -0.15850
H 4.81010 1.21390 -0.85680
H 3.88600 -0.12810 -1.60270
H 3.26040 1.54630 -1.68870
H 3.44120 -0.26940 1.95530
H 3.31710 -1.82600 0.16580
H 0.04280 -2.91380 -0.40340
33
01-Compound23-RS_miniMM3_9
O 4.57610 -1.15000 0.47400
C 3.25990 -0.60270 0.54000
C 3.27540 0.81240 -0.03230
C 2.33270 -1.46930 -0.30640
C 0.93040 -0.91890 -0.24610
C 0.70620 0.46640 -0.19030
C -0.62250 0.94800 -0.07750
C -0.86950 2.43540 0.07010
C -1.71470 0.04270 -0.07180
C -3.14940 0.53590 0.00230
C -3.68310 0.53520 1.43640
O -3.97350 -0.33040 -0.76530
C -1.45340 -1.33710 -0.12810
O -2.42930 -2.28660 -0.10620
C -0.14640 -1.80230 -0.20270
C 1.88170 1.38400 -0.27250
O 1.75980 2.55320 -0.56340
H -3.23650 1.55960 -0.42250
H 5.05320 -0.90560 1.25830
H 2.90100 -0.58990 1.59500
H 3.83720 1.48460 0.65650
H 3.82710 0.81770 -1.00090
H 2.67670 -1.49460 -1.36540
H 2.35700 -2.51710 0.07050
H -0.12860 2.91340 0.74630
H -0.83350 2.95240 -0.91450
H -1.85070 2.67220 0.52900
H -4.73600 0.89400 1.47910
H -3.66200 -0.48000 1.89160
H -3.08430 1.20120 2.09670
H -3.77150 -0.17160 -1.67980
H -3.32850 -1.91490 -0.13970
H 0.03130 -2.89020 -0.23830
33
01-Compound23-RS_miniMM3_10
O 4.58740 -1.10330 0.48890
C 3.25930 -0.58510 0.54530
C 3.27270 0.83630 -0.01110
C 2.34320 -1.44780 -0.31790
C 0.93740 -0.90820 -0.25440
C 0.70680 0.47570 -0.18990
C -0.62360 0.95070 -0.07290
C -0.87610 2.43600 0.08470
C -1.71170 0.04060 -0.07180
C -3.14820 0.52750 0.00770
C -3.68070 0.51230 1.44220
O -3.96890 -0.33610 -0.76660
C -1.44420 -1.33770 -0.13790
O -2.41620 -2.29140 -0.12210
C -0.13540 -1.79680 -0.21630
C 1.87790 1.39900 -0.26870
O 1.75070 2.56460 -0.57170
H -3.23970 1.55440 -0.40810
H 4.68640 -1.77410 1.15430
H 2.89600 -0.57300 1.59910
H 3.81820 1.50530 0.69370
H 3.83960 0.85640 -0.97080
H 2.69220 -1.45550 -1.37560
H 2.37240 -2.50090 0.04380
H -0.13150 2.91420 0.75670
H -0.85100 2.95830 -0.89750
H -1.85450 2.66520 0.55340
H -4.73500 0.86650 1.48890
H -3.65490 -0.50650 1.88880
H -3.08390 1.17520 2.10760
H -3.77320 -0.16380 -1.67990
H -3.31680 -1.92280 -0.15550
H 0.04660 -2.88370 -0.25950
33
02-Compound23-RR_miniMM3_11
O 2.95330 -0.86990 1.67270
C 3.36310 -0.82440 0.30830
C 3.41490 0.64080 -0.12420
C 2.35970 -1.57600 -0.56580
C 0.98400 -0.97800 -0.42750
C 0.83220 0.41030 -0.29240
C -0.46300 0.95120 -0.10290
C -0.62240 2.43880 0.13240
C -1.59890 0.10230 -0.09910
C -3.00070 0.66350 0.06240
C -3.48260 0.59870 1.51320
O -3.89610 -0.10850 -0.72580
C -1.41160 -1.28380 -0.23900
O -2.43510 -2.18200 -0.22960
C -0.13370 -1.80930 -0.38830
C 2.04830 1.26980 -0.38270
O 1.97840 2.43490 -0.70840
H -3.04640 1.71590 -0.29340
H 3.01070 -1.76300 1.98990
H 4.37590 -1.27860 0.20780
H 3.96320 1.23920 0.63920
H 4.01070 0.71610 -1.06380
H 2.67450 -1.53130 -1.63330
H 2.34970 -2.65270 -0.28120
H -0.64420 3.00080 -0.82770
H -1.54950 2.69330 0.68550
H 0.19570 2.85110 0.76110
H -4.51260 1.00770 1.61850
H -3.49980 -0.44290 1.90410
H -2.82590 1.18990 2.18960
H -3.71580 0.09750 -1.63530
H -3.31320 -1.76150 -0.21320
H -0.01340 -2.90090 -0.48580
33
02-Compound23-RR_miniMM3_12
O 3.00110 -0.86040 1.65310
C 3.37690 -0.83600 0.27730
C 3.41480 0.60830 -0.21770
C 2.33920 -1.61790 -0.52450
C 0.97150 -0.99660 -0.39900
C 0.82480 0.39530 -0.29140
C -0.47060 0.94330 -0.11420
C -0.62900 2.43530 0.09340
C -1.60990 0.09840 -0.09750
C -3.01120 0.66540 0.04770
C -3.49590 0.63640 1.49870
O -3.90760 -0.12260 -0.72340
C -1.42650 -1.29050 -0.20880
O -2.45220 -2.18610 -0.18270
C -0.15020 -1.82210 -0.34470
C 2.04340 1.25120 -0.39600
O 1.97280 2.42920 -0.66940
H -3.05480 1.70860 -0.33390
H 3.75660 -0.65730 2.19110
H 4.37880 -1.30830 0.15250
H 4.02870 1.22690 0.47650
H 3.93830 0.63690 -1.20180
H 2.63010 -1.64410 -1.59960
H 2.32190 -2.67460 -0.17240
H -0.61550 2.98340 -0.87480
H -1.57150 2.70570 0.61120
H 0.17010 2.85100 0.74410
H -3.51620 -0.39560 1.91430
H -2.83910 1.24220 2.16210
H -4.52520 1.05010 1.59240
H -3.71730 0.05230 -1.63730
H -3.32970 -1.76440 -0.16910
H -0.03260 -2.91610 -0.41770
33
02-Compound23-RR_miniMM3_13
O 3.07180 -0.94160 1.68130
C 3.37790 -0.83600 0.29140
C 3.41750 0.63360 -0.13020
C 2.35360 -1.59780 -0.54730
C 0.98240 -0.98640 -0.41420
C 0.83170 0.40410 -0.29750
C -0.46390 0.94820 -0.11620
C -0.62570 2.43920 0.09730
C -1.60060 0.10020 -0.10250
C -3.00340 0.66320 0.04730
C -3.48320 0.62870 1.49990
O -3.89920 -0.12710 -0.72210
C -1.41360 -1.28770 -0.22140
O -2.43650 -2.18620 -0.19860
C -0.13620 -1.81560 -0.36320
C 2.04930 1.26200 -0.38190
O 1.97890 2.43170 -0.68770
H -3.05100 1.70730 -0.33160
H 2.17370 -0.67970 1.84660
H 4.38530 -1.28370 0.12670
H 3.96800 1.22890 0.63400
H 4.00780 0.71800 -1.07260
H 2.65460 -1.58240 -1.61990
H 2.34020 -2.66680 -0.23420
H -0.62200 2.99000 -0.86940
H -1.56510 2.70420 0.62360
H 0.17760 2.85610 0.74200
H -4.51340 1.03880 1.59810
H -3.49900 -0.40470 1.91220
H -2.82610 1.23430 2.16290
H -3.73300 0.06940 -1.63630
H -3.31590 -1.76790 -0.18780
H -0.01690 -2.90910 -0.44610
33
02-Compound23-RR_miniMM3_14
O 2.95490 -0.86890 1.67240
C 3.36370 -0.82420 0.30770
C 3.41510 0.64070 -0.12580
C 2.35960 -1.57640 -0.56510
C 0.98390 -0.97810 -0.42700
C 0.83210 0.41020 -0.29220
C -0.46330 0.95110 -0.10300
C -0.62290 2.43870 0.13170
C -1.59910 0.10200 -0.09910
C -3.00090 0.66340 0.06200
C -3.48270 0.59960 1.51290
O -3.89640 -0.10930 -0.72560
C -1.41160 -1.28410 -0.23860
O -2.43500 -2.18230 -0.22900
C -0.13380 -1.80940 -0.38780
C 2.04810 1.26990 -0.38260
O 1.97790 2.43520 -0.70710
H -3.04670 1.71560 -0.29450
H 3.01330 -1.76160 1.99030
H 4.37630 -1.27830 0.20670
H 3.96460 1.23950 0.63660
H 4.00940 0.71540 -1.06640
H 2.67380 -1.53280 -1.63290
H 2.34970 -2.65280 -0.27950
H -0.64430 3.00040 -0.82860
H -1.55020 2.69330 0.68430
H 0.19500 2.85130 0.76060
H -4.51240 1.00930 1.61830
H -3.50060 -0.44190 1.90410
H -2.82540 1.19050 2.18900
H -3.71670 0.09660 -1.63510
H -3.31320 -1.76200 -0.21250
H -0.01340 -2.90110 -0.48500
33
02-Compound23-RR_miniMM3_15
O 4.67210 -1.09430 0.04830
C 3.39780 -0.55930 -0.30630
C 3.21690 0.80190 0.35530
C 2.31680 -1.50290 0.21330
C 0.92840 -0.91690 0.11200
C 0.69520 0.46760 0.01970
C -0.63930 0.94330 -0.04640
C -0.90190 2.43550 -0.04930
C -1.72560 0.03160 -0.07880
C -3.16140 0.51410 -0.19430
C -3.83950 0.62900 1.17270
O -3.90130 -0.42230 -0.96550
C -1.45770 -1.34490 0.00590
O -2.42850 -2.29990 0.00310
C -0.14980 -1.80120 0.10720
C 1.86840 1.39030 -0.01500
O 1.77830 2.54790 -0.35720
H -3.20650 1.49690 -0.71160
H 5.32250 -0.77800 -0.56770
H 3.31340 -0.46230 -1.41350
H 3.29390 0.71330 1.46290
H 4.03150 1.48880 0.02950
H 2.36910 -2.46230 -0.35110
H 2.51450 -1.74930 1.28230
H -0.25410 2.97040 0.67790
H -0.73830 2.87440 -1.05860
H -1.93340 2.70050 0.25860
H -4.89180 0.97940 1.07700
H -3.86350 -0.34380 1.71230
H -3.31250 1.35470 1.83150
H -3.61130 -0.33510 -1.86570
H -3.32090 -1.94130 -0.14890
H 0.03070 -2.88710 0.18050
33
02-Compound23-RR_miniMM3_16
O 3.00660 -0.85410 1.65230
C 3.37840 -0.83590 0.27540
C 3.41520 0.60610 -0.22650
C 2.33810 -1.62070 -0.52010
C 0.97100 -0.99770 -0.39580
C 0.82480 0.39450 -0.29020
C -0.47080 0.94290 -0.11390
C -0.62920 2.43500 0.09270
C -1.61030 0.09840 -0.09720
C -3.01180 0.66560 0.04600
C -3.49780 0.63810 1.49660
O -3.90740 -0.12330 -0.72510
C -1.42730 -1.29070 -0.20670
O -2.45320 -2.18610 -0.18060
C -0.15100 -1.82270 -0.34110
C 2.04330 1.25020 -0.39610
O 1.97250 2.42940 -0.66470
H -3.05520 1.70840 -0.33680
H 3.76270 -0.64450 2.18690
H 4.37970 -1.30910 0.14970
H 4.03560 1.22650 0.46030
H 3.93080 0.62940 -1.21480
H 2.62690 -1.65310 -1.59560
H 2.32040 -2.67540 -0.16230
H -0.61290 2.98280 -0.87570
H -1.57310 2.70610 0.60770
H 0.16820 2.85050 0.74560
H -4.52720 1.05190 1.58900
H -3.51850 -0.39350 1.91310
H -2.84160 1.24430 2.16000
H -3.71600 0.05030 -1.63910
H -3.33050 -1.76420 -0.16880
H -0.03380 -2.91680 -0.41270
33
02-Compound23-RR_miniMM3_17
O 4.68710 -1.04480 0.04900
C 3.40140 -0.53830 -0.30680
C 3.21820 0.82600 0.34660
C 2.32650 -1.48370 0.22250
C 0.93540 -0.90600 0.11800
C 0.69530 0.47700 0.02070
C -0.64200 0.94520 -0.04870
C -0.91260 2.43600 -0.05720
C -1.72330 0.02750 -0.07930
C -3.16160 0.50170 -0.19800
C -3.84090 0.61960 1.16810
O -3.89600 -0.44270 -0.96480
C -1.44820 -1.34730 0.01100
O -2.41380 -2.30750 0.01080
C -0.13810 -1.79620 0.11550
C 1.86360 1.40640 -0.01620
O 1.76370 2.56460 -0.35350
H -3.21180 1.48170 -0.72020
H 4.99160 -1.62580 -0.63820
H 3.31760 -0.43630 -1.41360
H 3.30390 0.74540 1.45420
H 4.02770 1.51400 0.00980
H 2.38240 -2.44730 -0.33420
H 2.52750 -1.72010 1.29300
H -0.74330 2.87350 -1.06620
H -1.94770 2.69660 0.24150
H -0.27340 2.97590 0.67400
H -4.89510 0.96350 1.07030
H -3.85960 -0.35080 1.71250
H -3.31820 1.35130 1.82360
H -3.61190 -0.35290 -1.86660
H -3.30800 -1.95410 -0.14280
H 0.04730 -2.88080 0.19360
33
02-Compound23-RR_miniMM3_18
O 3.07400 -0.93880 1.68090
C 3.37860 -0.83570 0.29060
C 3.41780 0.63320 -0.13360
C 2.35330 -1.59880 -0.54580
C 0.98230 -0.98690 -0.41290
C 0.83170 0.40370 -0.29690
C -0.46400 0.94790 -0.11580
C -0.62580 2.43890 0.09760
C -1.60080 0.09990 -0.10220
C -3.00350 0.66320 0.04620
C -3.48480 0.63020 1.49830
O -3.89870 -0.12780 -0.72310
C -1.41370 -1.28810 -0.22030
O -2.43670 -2.18650 -0.19730
C -0.13630 -1.81600 -0.36150
C 2.04910 1.26180 -0.38210
O 1.97840 2.43180 -0.68630
H -3.05080 1.70690 -0.33370
H 2.17610 -0.67660 1.84670
H 4.38580 -1.28360 0.12550
H 3.97100 1.22940 0.62790
H 4.00510 0.71560 -1.07790
H 2.65350 -1.58550 -1.61860
H 2.34000 -2.66710 -0.23060
H -0.62130 2.98980 -0.86920
H -1.56560 2.70410 0.62310
H 0.17700 2.85560 0.74300
H -4.51500 1.04070 1.59520
H -3.50120 -0.40280 1.91170
H -2.82830 1.23640 2.16150
H -3.73150 0.06760 -1.63740
H -3.31600 -1.76830 -0.18720
H -0.01700 -2.90950 -0.44380
33
02-Compound23-RR_miniMM3_19
O 4.67200 -1.09430 0.04600
C 3.39720 -0.55950 -0.30690
C 3.21730 0.80190 0.35420
C 2.31700 -1.50270 0.21480
C 0.92850 -0.91680 0.11320
C 0.69520 0.46760 0.02040
C -0.63940 0.94330 -0.04650
C -0.90220 2.43540 -0.05020
C -1.72550 0.03140 -0.07920

```
C -3.16130 0.51390 -0.19510
C -3.83900 0.62990 1.17180
O -3.90120 -0.42300 -0.96570
C -1.45750 -1.34500 0.00640
O -2.42840 -2.30000 0.00360
C -0.14970 -1.80130 0.10840
C 1.86840 1.39040 -0.01420
O 1.77780 2.54850 -0.35480
H -3.20640 1.49640 -0.71330
H 5.32140 -0.77840 -0.57140
H 3.31120 -0.46320 -1.41410
H 3.29590 0.71370 1.46180
H 4.03150 1.48870 0.02700
H 2.36910 -2.46290 -0.34820
H 2.51520 -1.74740 1.28390
H -0.25580 2.97070 0.67800
H -0.73670 2.87420 -1.05930
H -1.93420 2.70040 0.25540
H -4.89140 0.97980 1.07610
H -3.86260 -0.34240 1.71230
H -3.31200 1.35620 1.82990
H -3.61150 -0.33620 -1.86600
H -3.32060 -1.94160 -0.14910
H 0.03070 -2.88710 0.18240
33
02-Compound23-RR_miniMM3_20
O 4.69330 -1.08260 -0.05920
C 3.39750 -0.55590 -0.33920
C 3.23150 0.81720 0.30190
C 2.32240 -1.49910 0.19330
C 0.93450 -0.91220 0.10110
C 0.69870 0.47220 0.01090
C -0.63820 0.94430 -0.04920
C -0.90610 2.43570 -0.05190
C -1.72260 0.02980 -0.07770
C -3.16040 0.50780 -0.18850
C -3.83170 0.62700 1.18140
O -3.90190 -0.43400 -0.95160
C -1.45110 -1.34580 0.00650
O -2.41910 -2.30380 0.00870
C -0.14180 -1.79900 0.10130
C 1.86880 1.40050 -0.02570
O 1.76410 2.56760 -0.32860
H -3.21110 1.48790 -0.71050
H 4.82820 -1.14560 0.87840
H 3.28480 -0.45730 -1.44330
H 3.34490 0.75090 1.40800
H 4.03310 1.49940 -0.06400
H 2.37020 -2.46010 -0.36870
H 2.52490 -1.74230 1.26190
H -0.72570 2.87880 -1.05650
H -1.94330 2.69790 0.23730
H -0.27340 2.96950 0.68940
H -4.88540 0.97370 1.08970
H -3.84990 -0.34350 1.72540
H -3.30340 1.35710 1.83420
H -3.62170 -0.34590 -1.85470
H -3.31340 -1.94820 -0.13910
H 0.04050 -2.88470 0.17190
33
01-Compound23-RS_miniMMFF_1
O -3.32680 -0.91140 1.46770
C -3.45250 -0.73260 0.05610
C -3.38970 0.74560 -0.27010
C -2.37860 -1.50720 -0.69850
C -1.00940 -0.95340 -0.40620
C -0.80260 0.41130 -0.13450
C 0.50290 0.89460 0.18280
C 0.69130 2.35670 0.52610
C 1.61510 -0.00170 0.15200
C 3.03760 0.49110 0.39780
C 3.60970 1.19390 -0.82940
O 3.92020 -0.59460 0.72770
C 1.36270 -1.34960 -0.16310
O 2.35100 -2.29470 -0.21950
C 0.08370 -1.82020 -0.43430
C -1.99130 1.32710 -0.24920
O -1.84740 2.53960 -0.40240
H 3.05760 1.14540 1.27360
H -3.30980 -1.86670 1.65320
H -4.44120 -1.11810 -0.21780
H -3.78650 0.91510 -1.27820
H -3.99220 1.31310 0.44830
H -2.41980 -2.56000 -0.39340
H -2.55680 -1.45800 -1.77920
H 0.63520 2.96680 -0.38080
H -0.06350 2.68150 1.25010
H 1.64660 2.57550 1.00490
H 4.63360 1.53080 -0.63410
H 3.66370 0.50790 -1.68290
H 3.01360 2.06110 -1.12740
H 4.76080 -0.18720 1.00780
H 3.15720 -1.87600 0.15560
H -0.04790 -2.87530 -0.66460
33
01-Compound23-RS_miniMMFF_2
O -3.44450 -0.86880 1.40870
C -3.46670 -0.76630 -0.01480
C -3.36880 0.67830 -0.45140
C -2.33950 -1.59300 -0.61630
C -0.98430 -0.98880 -0.34910
C -0.79420 0.38810 -0.12720
C 0.51470 0.89820 0.14620
C 0.69840 2.37670 0.41520
C 1.63620 0.01120 0.14760
C 3.05580 0.52280 0.37010
C 3.63140 1.15040 -0.89570
O 3.93980 -0.53810 0.76960
C 1.39710 -1.34940 -0.11320
O 2.39620 -2.28440 -0.14150
C 0.12190 -1.84190 -0.35680
C -2.00140 1.28670 -0.22960
O -1.90370 2.51280 -0.21290
H 3.06920 1.22850 1.20540
H -4.20270 -0.37030 1.76100
H -4.42610 -1.18420 -0.34110
H -3.59640 0.76990 -1.51970
H -4.09010 1.28090 0.11330
H -2.37640 -2.60290 -0.18900
H -2.47100 -1.67560 -1.70200
H 0.50040 2.95190 -0.49490
H 0.03820 2.69950 1.22700
H 1.70290 2.65040 0.73870
H 3.02990 1.99100 -1.25360
H 4.65100 1.50840 -0.71490
H 3.69830 0.41150 -1.70280
H 4.77380 -0.11130 1.04020
H 3.19290 -1.84990 0.23540
H -0.00130 -2.90680 -0.54350
33
01-Compound23-RS_miniMMFF_3
O -3.40180 -1.00590 1.44700
C -3.45810 -0.75400 0.04140
C -3.39450 0.73370 -0.24140
C -2.37410 -1.51740 -0.70750
C -1.00880 -0.95520 -0.40730
C -0.80480 0.41160 -0.14450
C 0.50120 0.90020 0.16610
C 0.69150 2.36740 0.48760
C 1.61380 0.00380 0.14840
C 3.03720 0.49860 0.38930
C 3.61440 1.17440 -0.85040
O 3.91430 -0.58270 0.74530
C 1.36290 -1.34740 -0.15150
O 2.35090 -2.29300 -0.19300
C 0.08520 -1.82080 -0.42300
C -1.99920 1.32120 -0.24030
O -1.86430 2.53660 -0.37040
H 3.05600 1.17030 1.25180
H -2.48320 -0.91250 1.75530
H -4.43710 -1.12740 -0.28020
H -3.81200 0.93070 -1.23630
H -3.98320 1.28110 0.50370
H -2.41190 -2.57150 -0.40630
H -2.54430 -1.46290 -1.78930
H 0.60110 2.96700 -0.42370
H -0.04110 2.69360 1.23350
H 1.66120 2.60060 0.92860
H 4.63790 1.51490 -0.65850
H 3.67120 0.47030 -1.68880
H 3.01970 2.03540 -1.16890
H 4.75960 -0.17410 1.00960
H 3.15680 -1.87280 0.18110
H -0.04500 -2.87850 -0.64490
33
01-Compound23-RS_miniMMFF_4
O 4.64110 -1.10970 0.40890
C 3.33240 -0.55190 0.50260
C 3.28340 0.77370 -0.22630
C 2.33880 -1.51350 -0.13160
C 0.94730 -0.93990 -0.11820
C 0.70070 0.44800 -0.14660
C -0.64050 0.94010 -0.10530
C -0.89320 2.43200 -0.06540
C -1.73120 0.01790 -0.11500
C -3.17870 0.49360 -0.07160
C -3.64290 0.74490 1.35940
O -4.05750 -0.48440 -0.65150
C -1.43340 -1.35630 -0.12300
O -2.40570 -2.31920 -0.14270
C -0.12850 -1.82960 -0.11510
C 1.89430 1.36870 -0.24120
O 1.77160 2.58240 -0.38840
H -3.29750 1.38780 -0.69080
H 5.25850 -0.49700 0.84620
H 3.09680 -0.41550 1.56560
H 3.96060 1.47970 0.26930
H 3.63160 0.67110 -1.26100
H 2.62740 -1.74420 -1.16530
H 2.37070 -2.46350 0.41710
H -1.92840 2.69970 0.15090
H -0.31410 2.89450 0.74070
H -0.64000 2.88350 -1.02980
H -4.68130 1.09370 1.36760
H -3.61980 -0.18010 1.94760
H -3.02440 1.48800 1.87150
H -4.92460 -0.04910 -0.74950
H -3.24100 -1.86370 -0.38950
H 0.04020 -2.90490 -0.11720
33
01-Compound23-RS_miniMMFF_5
O 4.65030 -1.04430 0.44420
C 3.32420 -0.52640 0.51660
C 3.28500 0.82130 -0.17320
C 2.35670 -1.47650 -0.17380
C 0.95950 -0.92130 -0.14650
C 0.70280 0.46500 -0.15250
C -0.64050 0.94650 -0.08890
C -0.90390 2.43530 -0.01690
C -1.72420 0.01710 -0.10930
C -3.17440 0.48210 -0.05000
C -3.63940 0.68520 1.38830
O -4.04530 -0.48520 -0.65920
C -1.41710 -1.35460 -0.14400
O -2.38350 -2.32310 -0.17660
C -0.10900 -1.81940 -0.15130
C 1.88810 1.39490 -0.25410
O 1.74730 2.59780 -0.46370
H -3.29960 1.39460 -0.64060
H 4.67720 -1.86950 0.95970
H 3.06250 -0.41120 1.57620
H 3.92020 1.52450 0.37860
H 3.69080 0.75410 -1.18990
H 2.66090 -1.65610 -1.21290
H 2.39670 -2.44940 0.33250
H -0.28590 2.89600 0.76090
H -0.70900 2.90050 -0.98800
H -1.92780 2.68670 0.26410
H -3.61100 -0.25770 1.94700
H -3.02490 1.41520 1.92340
H -4.67990 1.02750 1.40750
H -4.91300 -0.05020 -0.75320
H -3.22120 -1.86880 -0.41780
H 0.06580 -2.89320 -0.17330
33
01-Compound23-RS_miniMMFF_6
O -3.56430 -0.81430 1.33000
C -3.46830 -0.78050 -0.09390
C -3.32250 0.64090 -0.59220
C -2.29850 -1.64110 -0.55190
C -0.95320 -0.99680 -0.31770
C -0.78200 0.39160 -0.14900
C 0.52650 0.94130 0.01550
C 0.71120 2.44030 0.11040
C 1.65190 0.06870 0.08890
C 3.05940 0.59520 0.32130
C 3.77920 0.87780 -0.99190
O 3.83010 -0.37620 1.04440
C 1.43690 -1.30780 -0.10040
O 2.45600 -2.22230 -0.08830
C 0.16910 -1.83060 -0.31420
C -2.01650 1.25360 -0.14530
O -1.98770 2.44050 0.17170
H 3.05410 1.48940 0.95130
H -4.30660 -0.24460 1.59940
H -4.39890 -1.21330 -0.47810
H -3.36720 0.68930 -1.68570
H -4.13420 1.25570 -0.18550
H -2.33470 -2.60150 -0.02250
H -2.38990 -1.83960 -1.62710
H 1.74490 2.76100 -0.03140
H 0.16230 2.94640 -0.69040
H 0.38190 2.80120 1.08940
H 4.79310 1.24410 -0.79710
H 3.88730 -0.03350 -1.59140
H 3.25030 1.62210 -1.59520
H 3.49790 -0.34730 1.96240
H 3.26630 -1.76280 0.22030
H 0.06120 -2.90400 -0.45840
33
01-Compound23-RS_miniMMFF_7
O 4.63490 -1.08020 0.52860
C 3.31350 -0.54380 0.55760
C 3.28750 0.81020 -0.12110
C 2.34990 -1.49260 -0.14080
C 0.95470 -0.93170 -0.12690
C 0.70430 0.45540 -0.13950
C -0.63560 0.94470 -0.07990
C -0.89140 2.43450 -0.00670
C -1.72390 0.02150 -0.10680
C -3.17140 0.49440 -0.05500
C -3.64840 0.68200 1.38160
O -4.04070 -0.46080 -0.68470
C -1.42410 -1.35210 -0.13940
O -2.39640 -2.31410 -0.18120
C -0.11840 -1.82420 -0.13570
C 1.89210 1.37850 -0.25280
O 1.75780 2.57010 -0.52250
H -3.28640 1.41560 -0.63430
H 4.92190 -1.15430 -0.39820
H 3.03050 -0.43560 1.61200
H 3.89850 1.51300 0.45780
H 3.72870 0.75240 -1.12350
H 2.66180 -1.67300 -1.17740
H 2.38360 -2.46510 0.36650
H -0.24680 2.89870 0.74700
H -0.72730 2.89420 -0.98610
H -1.90460 2.68770 0.31010
H -4.68680 1.03080 1.39560
H -3.63150 -0.26820 1.92840
H -3.03410 1.40100 1.93150
H -4.90710 -0.02270 -0.77650
H -3.22870 -1.85480 -0.43110
H 0.05130 -2.89890 -0.15080
33
01-Compound23-RS_miniMMFF_8
O -3.64330 -0.85600 1.30690
C -3.48140 -0.76430 -0.10910
C -3.33730 0.67690 -0.55110
C -2.31330 -1.61620 -0.58330
C -0.96870 -0.98210 -0.32470
C -0.78870 0.40450 -0.15350
C 0.52410 0.94540 0.00830
C 0.72200 2.44370 0.09570
C 1.64370 0.06580 0.08570
C 3.05620 0.58500 0.30720
C 3.76820 0.85420 -1.01320
O 3.82580 -0.38950 1.02710
C 1.41810 -1.30980 -0.09380
O 2.42890 -2.23250 -0.06880
C 0.14760 -1.82380 -0.31280
C -2.01800 1.27360 -0.12280
O -1.96930 2.44990 0.22660
H 3.06040 1.48400 0.93090
H -2.81530 -0.59160 1.74460
H -4.40510 -1.17270 -0.53460
H -3.40940 0.76720 -1.64050
H -4.13590 1.27660 -0.09860
H -2.35700 -2.59110 -0.08190
H -2.39820 -1.78350 -1.66440
H 0.15580 2.95360 -0.69060
H 0.42200 2.80850 1.08280
H 1.75410 2.75510 -0.07580
H 4.78580 1.21550 -0.82820
H 3.86650 -0.06200 -1.60700
H 3.24010 1.59740 -1.61830
H 3.59000 -0.27550 1.96750
H 3.24030 -1.77700 0.24470
H 0.03490 -2.89670 -0.46010
33
01-Compound23-RS_miniMMFF_9
O 4.61890 -1.13250 0.41730
C 3.31080 -0.57260 0.50650
C 3.28290 0.78200 -0.17100
C 2.32590 -1.50680 -0.18210
C 0.93500 -0.93490 -0.13700
C 0.69810 0.45430 -0.13170
C -0.63430 0.95540 -0.03840
C -0.87950 2.44320 0.08500
C -1.72990 0.04380 -0.07310
C -3.17140 0.52680 -0.03490
C -3.70830 0.58090 1.39040
O -4.00430 -0.37040 -0.78370
C -1.44750 -1.33380 -0.10950
O -2.43030 -2.28680 -0.12500
C -0.14650 -1.81790 -0.13040
C 1.89130 1.36600 -0.26360
O 1.76200 2.56290 -0.51190
H -3.28130 1.50230 -0.51790
H 5.23060 -0.53940 0.88820
H 3.05560 -0.47520 1.56940
H 3.91610 1.47800 0.39230
H 3.69860 0.71920 -1.18390
H 2.61720 -1.68130 -1.22570
H 2.36010 -2.48410 0.31630
H -0.18520 2.89260 0.80250
H -0.78360 2.92580 -0.89230
H -1.86650 2.68230 0.48660
H -4.75010 0.92030 1.39020
H -3.70430 -0.41130 1.85610
H -3.12260 1.25620 2.02160
H -3.84020 -0.16410 -1.72390
H -3.28800 -1.83000 -0.26240
H 0.01480 -2.89400 -0.15220
33
01-Compound23-RS_miniMMFF_10
O 4.63470 -1.06240 0.43640
C 3.31000 -0.54180 0.51290
C 3.28120 0.82490 -0.14140
C 2.34360 -1.47250 -0.20720
C 0.94730 -0.91720 -0.15490
C 0.69930 0.47000 -0.13460
C -0.63620 0.95940 -0.03100
C -0.89220 2.44400 0.10940
C -1.72450 0.03990 -0.07180
C -3.16930 0.51150 -0.02230
C -3.70150 0.54130 1.40560
O -3.99720 -0.38300 -0.78010
C -1.43130 -1.33500 -0.12550
O -2.40670 -2.29530 -0.15010
C -0.12660 -1.80910 -0.15540
C 1.88440 1.39220 -0.26510
O 1.73950 2.58170 -0.53850
H -3.28830 1.49320 -0.49060
H 4.65720 -1.89750 0.93590
H 3.04190 -0.45260 1.57340
H 3.88920 1.51950 0.45010
H 3.72440 0.78410 -1.14390
H 2.64480 -1.61690 -1.25250
H 2.38590 -2.46130 0.26670
H -0.18800 2.89510 0.81590
H -0.82080 2.93510 -0.86560
H -1.87340 2.66890 0.53360
H -4.74570 0.87250 1.41390
H -3.68830 -0.45740 1.85720
H -3.11870 1.21210 2.04440
H -3.86230 -0.14170 -1.71640
H -3.26690 -1.84240 -0.28700
H 0.04150 -2.88360 -0.19080
33
02-Compound23-RR_miniMMFF_11
O 3.17480 -0.75700 1.68210
C 3.40850 -0.77170 0.27290
C 3.41660 0.65080 -0.24970
C 2.36830 -1.61800 -0.45190
C 0.99940 -1.00080 -0.34080
C 0.81920 0.39280 -0.26530
C -0.48620 0.94360 -0.10300
C -0.65630 2.43710 0.06920
C -1.62050 0.07930 -0.12330
C -3.03470 0.62090 0.03620
C -3.43330 0.72450 1.50480
O -3.98250 -0.24030 -0.61570
C -1.39520 -1.30290 -0.25170
O -2.41910 -2.21010 -0.29910
C -0.11720 -1.83850 -0.34370
C 2.03930 1.25850 -0.41500
O 1.94160 2.43960 -0.74660
H -3.12560 1.58850 -0.46720
H 3.11980 -1.67820 1.99080
H 4.40200 -1.21160 0.12860
H 3.98120 1.29700 0.43220
H 3.89250 0.67500 -1.23710
H 2.62600 -1.71880 -1.51290
H 2.35360 -2.62080 -0.00720
H -0.58890 2.93890 -0.90080
H -1.61050 2.71120 0.52330
H 0.09620 2.83720 0.75660
H -4.44880 1.12530 1.59620
H -3.44420 -0.26330 1.98000
H -2.75490 1.36720 2.07360
H -4.82430 0.25140 -0.64130
H -3.23490 -1.69290 -0.48200
H -0.00530 -2.91710 -0.42920
33
02-Compound23-RR_miniMMFF_12
O 3.26600 -0.72400 1.64560
C 3.42480 -0.79640 0.22860
C 3.40820 0.58740 -0.38370
C 2.33790 -1.67520 -0.37580
C 0.97940 -1.02820 -0.28990
C 0.80930 0.36860 -0.25970
C -0.49640 0.93130 -0.12270
C -0.66130 2.43000 0.00520
C -1.63730 0.07360 -0.12330
C -3.04990 0.62710 0.01160
```

C -3.45030 0.79060 1.47440
O -4.00230 -0.25270 -0.60840
C -1.42030 -1.31280 -0.20700
O -2.44870 -2.21560 -0.23350
C -0.14500 -1.85720 -0.27280
C 2.03650 1.22540 -0.42300
O 1.95470 2.42830 -0.66850
H -3.13570 1.57370 -0.53060
H 4.01170 -0.21420 2.00840
H 4.39960 -1.26460 0.04950
H 4.06200 1.25350 0.19130
H 3.77160 0.54380 -1.41700
H 2.55860 -1.87920 -1.43040
H 2.31830 -2.63150 0.16190
H -0.53320 2.90870 -0.97050
H -1.63640 2.72840 0.39420
H 0.05440 2.83620 0.72760
H -3.46620 -0.17780 1.98800
H -2.76980 1.45220 2.01860
H -4.46410 1.19910 1.54820
H -4.84460 0.23730 -0.64190
H -3.26160 -1.70000 -0.43080
H -0.03740 -2.93880 -0.31860
33
02-Compound23-RR_miniMMFF_13
O 3.25110 -0.84680 1.68130
C 3.41630 -0.78930 0.26270
C 3.41720 0.64790 -0.22030
C 2.36760 -1.62770 -0.45670
C 1.00090 -1.00430 -0.33860
C 0.82090 0.38980 -0.27430
C -0.48490 0.94250 -0.11730
C -0.65710 2.43810 0.03610
C -1.61940 0.07780 -0.12640
C -3.03510 0.62020 0.02360
C -3.43350 0.74940 1.49020
O -3.98120 -0.25370 -0.61280
C -1.39380 -1.30560 -0.23690
O -2.41640 -2.21420 -0.26750
C -0.11590 -1.84150 -0.32710
C 2.04300 1.25370 -0.41060
O 1.94910 2.43780 -0.72900
H -3.12750 1.57820 -0.49740
H 2.31510 -0.69960 1.90330
H 4.40500 -1.22130 0.06920
H 3.96140 1.28120 0.48960
H 3.91560 0.70030 -1.19570
H 2.61720 -1.72430 -1.52000
H 2.35260 -2.63130 -0.01420
H -0.56820 2.92990 -0.93730
H -1.62030 2.71990 0.46580
H 0.08130 2.84320 0.73590
H -4.44990 1.14960 1.57470
H -3.44230 -0.23000 1.98290
H -2.75630 1.40340 2.04730
H -4.82470 0.23450 -0.64640
H -3.23390 -1.70330 -0.46000
H -0.00370 -2.92150 -0.40190
33
02-Compound23-RR_miniMMFF_14
O 3.17840 -0.78690 1.65920
C 3.40160 -0.78070 0.24820
C 3.41050 0.65080 -0.25130
C 2.35230 -1.61350 -0.48080
C 0.98610 -0.99450 -0.34540
C 0.81420 0.39840 -0.24560
C -0.48330 0.95340 -0.05260
C -0.64580 2.43970 0.17560
C -1.62150 0.09810 -0.08730
C -3.03170 0.64510 0.06130
C -3.50070 0.60580 1.51090
O -3.94080 -0.14720 -0.71670
C -1.40890 -1.28540 -0.22900
O -2.43830 -2.18790 -0.26280
C -0.13490 -1.82700 -0.34330
C 2.03320 1.25970 -0.41120
O 1.93020 2.43690 -0.75500
H -3.11220 1.66260 -0.33300
H 3.13580 -1.71270 1.95630
H 4.39270 -1.22090 0.08950
H 3.97350 1.28740 0.44080
H 3.88770 0.68910 -1.23770
H 2.59920 -1.69720 -1.54570
H 2.33950 -2.62310 -0.05210
H -0.65860 2.97080 -0.78070
H -1.56190 2.68270 0.71940
H 0.15450 2.82970 0.81300
H -4.52200 0.99410 1.58930
H -3.52800 -0.42080 1.89430
H -2.85250 1.19700 2.16520
H -3.80600 0.12860 -1.64350
H -3.27570 -1.68030 -0.33030
H -0.02870 -2.90520 -0.43930
33
02-Compound23-RR_miniMMFF_15
O 4.68990 -1.08790 0.04490
C 3.40740 -0.56290 -0.28900
C 3.23110 0.80520 0.33490
C 2.33380 -1.50420 0.23970
C 0.94650 -0.93320 0.09530
C 0.69400 0.45070 -0.00650
C -0.64680 0.93660 -0.08830
C -0.90300 2.42720 -0.14410
C -1.73130 0.00890 -0.12100
C -3.17910 0.47680 -0.20700
C -3.74700 0.79920 1.17130
O -4.00910 -0.53740 -0.79670
C -1.43060 -1.36100 -0.01880
O -2.39850 -2.32850 -0.03950
C -0.12800 -1.82520 0.10610
C 1.88480 1.37480 -0.03550
O 1.78890 2.56240 -0.33090
H -3.25530 1.33680 -0.87920
H 5.36170 -0.50280 -0.34760
H 3.33980 -0.49870 -1.38250
H 3.32570 0.77610 1.42650
H 4.01580 1.47750 -0.03210
H 2.41230 -2.45910 -0.29610
H 2.51590 -1.73040 1.29860
H -0.37000 2.93650 0.66560
H -0.59620 2.82600 -1.11600
H -1.94930 2.70050 -0.00040
H -4.78420 1.14110 1.08560
H -3.76450 -0.09370 1.80740
H -3.16950 1.57190 1.68730
H -4.86870 -0.11490 -0.97980
H -3.21410 -1.89610 -0.37670
H 0.04150 -2.89660 0.19510
33
02-Compound23-RR_miniMMFF_16
O 3.24710 -0.77320 1.63160
C 3.41240 -0.80560 0.21380
C 3.40400 0.59700 -0.35700
C 2.32490 -1.66450 -0.41960
C 0.96770 -1.01940 -0.30380
C 0.80570 0.37680 -0.24210
C -0.49110 0.94380 -0.06640
C -0.64760 2.43560 0.12850
C -1.63550 0.09510 -0.08530
C -3.04350 0.65360 0.04120
C -3.51990 0.65830 1.48890
O -3.95460 -0.15320 -0.71940
C -1.43170 -1.29250 -0.18960
O -2.46620 -2.18950 -0.20500
C -0.16050 -1.84320 -0.28290
C 2.03050 1.22900 -0.42460
O 1.94050 2.42240 -0.71090
H -3.11650 1.65930 -0.38310
H 3.99980 -0.28840 2.01370
H 4.38680 -1.27120 0.02590
H 4.03580 1.25180 0.25410
H 3.79730 0.58170 -1.38020
H 2.54190 -1.83100 -1.48150
H 2.30730 -2.63910 0.08410
H -0.62250 2.94920 -0.83720
H -1.57880 2.70040 0.63480
H 0.13350 2.82830 0.78760
H -4.53940 1.05450 1.55100
H -3.55490 -0.35670 1.90120
H -2.87160 1.26430 2.12930
H -3.78440 0.06570 -1.65550
H -3.30190 -1.68040 -0.27650
H -0.05860 -2.92470 -0.34490
33
02-Compound23-RR_miniMMFF_17
O 4.71030 -1.01680 0.03920
C 3.41270 -0.52780 -0.29140
C 3.23310 0.84500 0.32130
C 2.34970 -1.47090 0.25450
C 0.95860 -0.91420 0.10420
C 0.69460 0.46710 -0.00510
C -0.65120 0.93930 -0.09350
C -0.92210 2.42720 -0.15870
C -1.72730 0.00130 -0.12260
C -3.17920 0.45530 -0.21340
C -3.75020 0.78700 1.16150
O -4.00050 -0.57250 -0.79210
C -1.41410 -1.36500 -0.01220
O -2.37190 -2.34230 -0.02920
C -0.10750 -1.81610 0.11780
C 1.87740 1.40360 -0.03360
O 1.76300 2.59350 -0.31360
H -3.26310 1.30720 -0.89500
H 4.84400 -1.85370 -0.43940
H 3.34130 -0.46140 -1.38450
H 3.34280 0.82680 1.41190
H 4.00840 1.51850 -0.06310
H 2.43530 -2.43270 -0.26750
H 2.53710 -1.68010 1.31590
H -0.60410 2.82600 -1.12710
H -1.97310 2.69110 -0.03420
H -0.40760 2.94430 0.65800
H -4.79050 1.11850 1.07230
H -3.75960 -0.09920 1.80690
H -3.17990 1.57050 1.66930
H -4.86330 -0.15870 -0.98010
H -3.19310 -1.91870 -0.36450
H 0.07010 -2.88550 0.21390
33
02-Compound23-RR_miniMMFF_18
O 3.25230 -0.88340 1.65830
C 3.40840 -0.79800 0.24010
C 3.41080 0.64950 -0.21320
C 2.35230 -1.62090 -0.48820
C 0.98790 -0.99720 -0.34520
C 0.81600 0.39620 -0.25600
C -0.48200 0.95300 -0.06800
C -0.64670 2.44230 0.14130
C -1.62020 0.09700 -0.09210
C -3.03160 0.64430 0.04940
C -3.49580 0.63130 1.50120
O -3.94170 -0.16510 -0.70910
C -1.40670 -1.28780 -0.21680
O -2.43460 -2.19150 -0.23450
C -0.13270 -1.82940 -0.32920
C 2.03710 1.25530 -0.40820
O 1.93880 2.43370 -0.74550
H -3.11480 1.65390 -0.36420
H 2.31930 -0.73370 1.89100
H 4.39450 -1.22850 0.03190
H 3.94620 1.27110 0.51330
H 3.91850 0.72060 -1.18290
H 2.59290 -1.69610 -1.55520
H 2.33910 -2.63320 -0.06620
H -0.64170 2.96300 -0.82090
H -1.57160 2.69440 0.66540
H 0.14290 2.83720 0.78900
H -4.51770 1.01910 1.57550
H -3.51980 -0.38800 1.90360
H -2.84690 1.23580 2.14230
H -3.84010 0.11800 -1.63790
H -3.27340 -1.68750 -0.31440
H -0.02620 -2.90920 -0.41520
33
02-Compound23-RR_miniMMFF_19
O 4.67840 -1.09740 0.05350
C 3.40130 -0.56520 -0.28910
C 3.22100 0.79710 0.34820
C 2.31880 -1.51250 0.21360
C 0.93290 -0.93170 0.09050
C 0.68870 0.45500 0.01030
C -0.64710 0.95340 -0.03420
C -0.89920 2.44510 -0.02780
C -1.73550 0.03520 -0.08690
C -3.17650 0.51000 -0.18760
C -3.81430 0.66750 1.18760
O -3.95270 -0.44660 -0.92390
C -1.44910 -1.33930 0.00090
O -2.42710 -2.29740 -0.01010
C -0.14880 -1.81550 0.10640
C 1.88370 1.36760 -0.04860
O 1.79570 2.53910 -0.40400
H -3.25230 1.44580 -0.74950
H 5.35660 -0.50780 -0.32160
H 3.34690 -0.48770 -1.38240
H 3.29200 0.75610 1.44110
H 4.01450 1.47170 0.00560
H 2.38980 -2.45290 -0.34840
H 2.50150 -1.76920 1.26560
H -0.27150 2.94110 0.71970
H -0.71240 2.86480 -1.02080
H -1.91840 2.70710 0.26400
H -4.85360 0.99890 1.08750
H -3.84260 -0.28700 1.72580
H -3.27630 1.39200 1.80660
H -3.72420 -0.30900 -1.86300
H -3.27410 -1.85760 -0.23940
H 0.01380 -2.88900 0.18530
33
02-Compound23-RR_miniMMFF_20
O 4.69890 -1.07530 -0.11230
C 3.39110 -0.56110 -0.35660
C 3.25920 0.82730 0.23130
C 2.34710 -1.48080 0.25820
C 0.95750 -0.92160 0.11030
C 0.69760 0.45920 -0.01310
C -0.64840 0.92930 -0.13230
C -0.91540 2.41540 -0.24600
C -1.72760 -0.00760 -0.13730
C -3.17970 0.44780 -0.23580
C -3.72700 0.87410 1.12260
O -4.01990 -0.60670 -0.73360
C -1.41630 -1.37130 0.00340
O -2.37340 -2.34920 0.01590
C -0.10980 -1.82170 0.13560
C 1.88110 1.39950 -0.00250
O 1.75390 2.61470 -0.12570
H -3.26880 1.25220 -0.97180
H 4.86170 -1.07280 0.84700
H 3.25480 -0.51950 -1.44440
H 3.46600 0.83240 1.30800
H 4.00000 1.48650 -0.23740
H 2.42110 -2.46430 -0.22320
H 2.55730 -1.64290 1.32340
H -0.47090 2.80890 -1.16580
H -1.97280 2.67900 -0.28290
H -0.51810 2.93800 0.63030
H -4.76830 1.20140 1.02870
H -3.72630 0.03360 1.82650
H -3.14770 1.68910 1.56650
H -4.88510 -0.19950 -0.92510
H -3.20220 -1.93060 -0.30640
H 0.06700 -2.89020 0.24490

4. Electronic Circular Dichroism

4.1. Computational details

Time-Dependent Density Functional Theory calculations were undertaken to predict ECD spectra for compound **21**. A conformational search was done with the Mixed Torsional/Low Mode Sampling protocol in gas phase using the MMFF force field, 21 kJ/mol and MAD 0.5 Å as energy cutoff and geometric creiteria respectively. Only one conformer was found under these conditions due to the rigidity of the molecule. Afterwards, structures of both enantiomers (9*R* and 9*S*) were optimized at the PCM/B3LYP/6-31G(d) level of theory and subsequently submitted to TDDFT/ECD calculations at B3LYP/6-31G(d) as descibed in the manuscript and at ωB97XD (figure S76). Calculations for the 9*S* enantiomer, at both levels of theory, matched better than the oposite enantiomer.

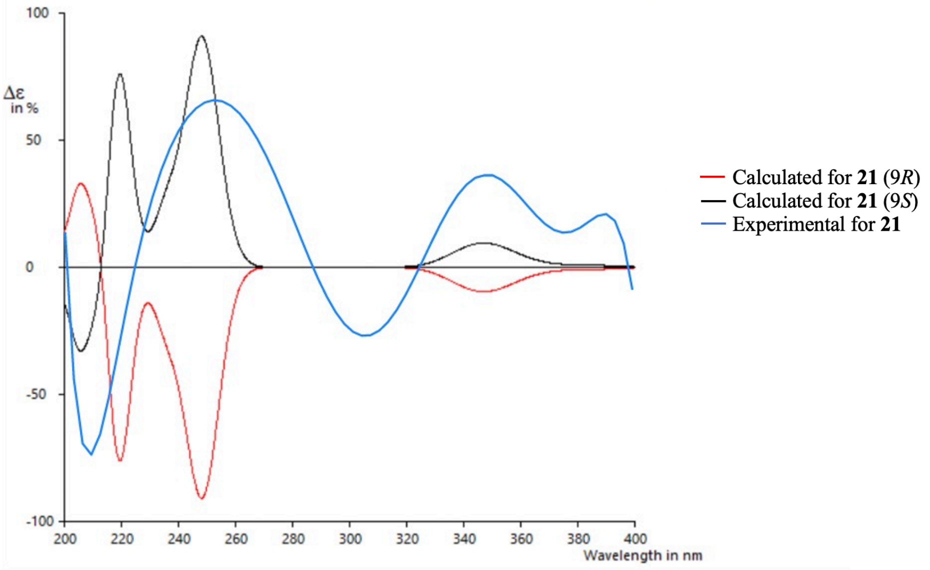

Figure S76. Experimental and calculated ECD spectra for compound **21** at ωB97XD.

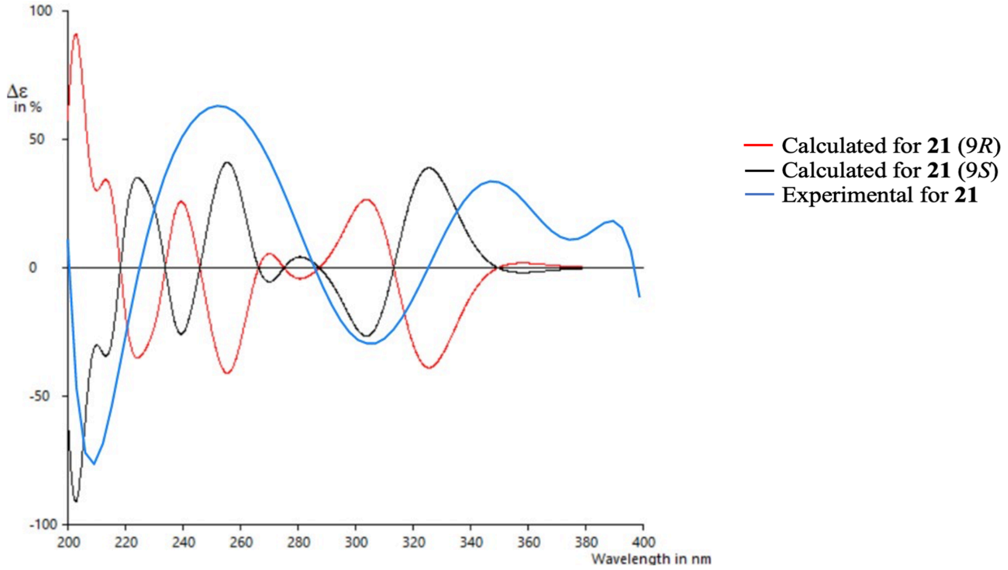

Figure S77. Experimental and calculated ECD spectra for compound **21** at B3LYP/6-31G(d).
